# Information-based autonomous reconfiguration in systems of interacting DNA nanostructures

**DOI:** 10.1038/s41467-018-07805-7

**Published:** 2018-12-18

**Authors:** Philip Petersen, Grigory Tikhomirov, Lulu Qian

**Affiliations:** 1 Biology, California Institute of Technology, Pasadena, CA 91125 USA; 2 Bioengineering, California Institute of Technology, Pasadena, CA 91125 USA; 3 Computer Science, California Institute of Technology, Pasadena, CA 91125 USA

## Abstract

The dynamic interactions between complex molecular structures underlie a wide range of sophisticated behaviors in biological systems. In building artificial molecular machines out of DNA, an outstanding challenge is to develop mechanisms that can control the kinetics of interacting DNA nanostructures and that can compose the interactions together to carry out system-level functions. Here we show a mechanism of DNA tile displacement that follows the principles of toehold binding and branch migration similar to DNA strand displacement, but occurs at a larger scale between interacting DNA origami structures. Utilizing this mechanism, we show controlled reaction kinetics over five orders of magnitude and programmed cascades of reactions in multi-structure systems. Furthermore, we demonstrate the generality of tile displacement for occurring at any location in an array in any order, illustrated as a tic-tac-toe game. Our results suggest that tile displacement is a simple-yet-powerful mechanism that opens up the possibility for complex structural components in artificial molecular machines to undergo information-based reconfiguration in response to their environments.

## Abstract (web summary)

Strand displacement is commonly used in DNA nanotechnology to program dynamic interactions between individual DNA strands. Here, the authors describe a tile displacement principle that is similar in concept but occurs on a larger structural level: the displacement reactions take place between DNA origami tiles, allowing reconfiguration of entire systems of interacting DNA structures.

## Introduction

Molecular structures, such as the cell membrane, provide compartmentalization and spatial organization key to the functionality of natural molecular machines in biological organisms. DNA, an information-bearing molecule, has been used to engineer the self-assembly of prescribed nanostructures^1^. Among various techniques, DNA origami^2^ is particularly robust for organizing other kinds of molecules into any desired patterns, which have been used for studying unknown aspects of biomolecular interactions^3,4^, fabricating devices with nanoscale features^5,6^, or functioning as a breadboard for biochemical circuits^7^ and a testing ground for molecular robots^8–10^. Individual DNA origami structures can be programmed to create larger structures hierarchically, recently approaching the size of a small bacterium^11,12^. However, unlike the sophisticated dynamic interactions between complex protein molecules, for example as seen in alternative sigma factors reconfiguring the function of RNA polymerase^13^, the designed interactions between DNA origami structures have so far been limited to just binding and unbinding.

The mechanism of DNA strand displacement^14^ has been used to program dynamic interactions between small DNA molecules that give rise to sophisticated system-level behaviors^15–18^, owing to the fact that a wide range of kinetics can be controlled by varying the strength of a toehold domain^19,20^ and that the toehold for initiating a downstream reaction can be hidden until the strand has been released from an upstream reaction (termed “toehold sequestering”)^21,22^. If a similar mechanism that has these two properties governed the interactions between complex DNA nanostructures rather than between individual DNA strands, it would give rise to sophisticated autonomous reconfiguration in systems of DNA nanostructures, much like the autonomous information processing in DNA circuits and devices.

While the fundamental principle of DNA base pairing relies on the complementarity of Watson–Crick binding, a similar principle can be exploited at a much larger scale to program the interactions between DNA origami structures^23^. In a DNA strand displacement process, there are two fundamental principles: fraying of double strands allows for an invading strand to initiate a competition with a previously bound strand for binding to its complementary strand; structural flexibility of single strands allows for the unbounded part of both the invading strand and the competing strand to be pushed out of the way in order for the branch migration to take place. If these two principles can be satisfied in individual DNA origami structures and their complexes, it will be possible to create similar displacement reactions and use them to program dynamic system-level functions at a much larger scale.

Here we show a mechanism of DNA tile displacement, in which an invader DNA origami tile displaces another tile from an array of tiles, enabled by a binding domain on the tile edge functioning as a toehold. We measure the kinetics of tile displacement reactions with varying toehold strengths, and show that the kinetics can be controlled over a range of five orders of magnitude, reaching a maximum effective rate of ~4.5 × 10^5^ M^−1^ s^−1^. Using this mechanism, we develop three example systems for general-purpose reconfiguration in DNA nanostructures: competitive, sequential, and cooperative tile displacement, each illustrating a basic type of information processing within structural reconfiguration. Finally, we demonstrate the generality of tile displacement reactions through a multi-step reconfiguration pathway shown as a tic-tac-toe game, where each player has nine unique DNA origami tiles that can be used to make nine possible moves in any order on a 264 by 264 nanometer game board.

## Results

### Concept of DNA tile displacement

In earlier work, we designed a square DNA origami tile to construct arrays with combinatorial patterns^24^. To form 2 by 2 arrays, we designed a single tile such that four rotated copies can bind to each other to form a larger square (Supplementary Fig. 1a). We explored several edge designs and expected to obtain a high yield of the arrays only when the edge interactions were weak enough to eliminate kinetic traps. However, a surprisingly high yield was observed with a relatively strong edge design (Supplementary Fig. 1b), for which we expected the formation of arrays to take place at a temperature where the binding between any two complementary tile edges was largely irreversible. If the only possible reactions were just binding, then a fraction of the final structures should be trimers (Supplementary Fig. 1c, solid arrows), conflicting with the observed high yield of 2 by 2 arrays. There were two possible explanations: first, the arrays actually formed at a temperature high enough for reversible binding. Second, a dimer and a trimer could undergo a displacement reaction to yield a 2 by 2 array while releasing a monomer, and two copies of trimers could also undergo a displacement reaction to yield a 2 by 2 array while releasing a dimer (Supplementary Fig. 1c, dotted arrows). With displacement reactions, the lack of spontaneous unbinding will not result in kinetic traps and all tiles will eventually self-assemble into the desired 2 by 2 arrays (Supplementary Fig. 1c, simulations). While the observation did not rule out either explanation, or a combination of the two, the second possibility provoked us to explore further. If it exists, displacement will affect the understanding of self-assembly not only in arrays with designed sizes but also in unbounded arrays. For example, dynamic rearrangement of DNA origami structures has been observed on a liquid bilayer^25,26^, hinting at the possibility that a well-formed structure with stronger edge interactions could displace a malformed one with weaker edge interactions in a periodic DNA origami array.

To investigate if one DNA origami structure can displace another from a complex of structures without any spontaneous unbinding within the complex, we performed two experiments: at room temperature, a complex of two square tiles were mixed together with a triangular tile^27^ that either has the same binding domain as one of the squares or has an additional binding domain that is complementary to the other square (Supplementary Fig. 1d, e). The two squares remained bound to each other in the former experiment but one square swapped with the triangle in the latter, suggesting that displacement indeed occurred. With this initial evidence, we set off to explore if the desired properties of DNA strand displacement reactions can be reproduced to program the dynamic interactions between DNA origami structures.

**Fig. 1:**
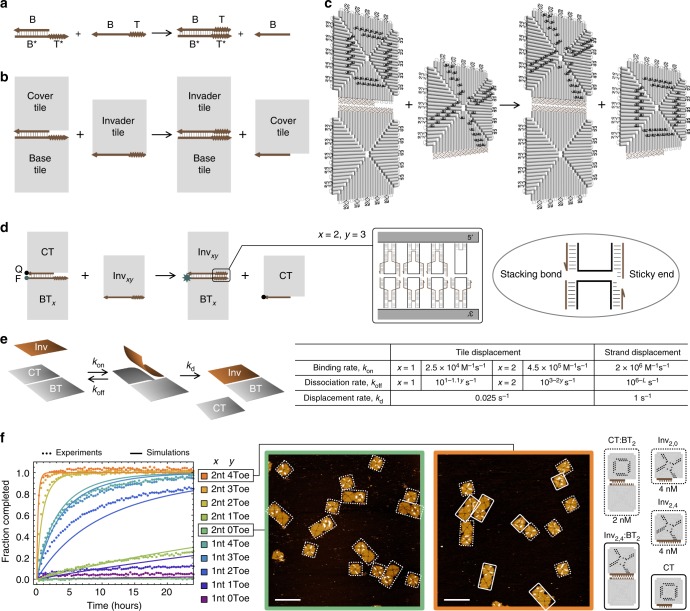
Concept and kinetics of DNA tile displacement. **a** Domain-level diagram of a DNA strand displacement reaction. T is a short toehold domain of typically 3 to 8 nucleotides. B is a long branch migration domain of typically 15 to 20 nucleotides. Asterisks in the domain names indicate sequence complementarity. **b** Domain-level and **c** origami-level diagram of a DNA tile displacement reaction. Toehold and branch migration domains are composed of 4 and 7 edge staples, respectively. Invader tile and cover tile are labeled with double-stranded staple extensions in an X and O pattern, respectively. **d** Tile displacement reactions with varying toeholds. CT is a cover tile. BT_*x*_ is a base tile with *x* = 1 or 2 indicating the number of nucleotides in the sticky end of each of the 4 edge staples in the toehold domain. Inv_*xy*_ is an invader tile with *y* = 0 through 4 indicating the number of edge staples in the toehold domain. F and Q indicate a fluorophore- and quencher-labeled edge staple, respectively. **e** Model and rate parameters of tile displacement in comparison with strand displacement. *L* is the number of nucleotides in the toehold domain of a strand displacement reaction with average DNA sequences. **f** Fluorescence kinetics experiments, simulations, and AFM images of the tile displacement reactions. Experiments were performed at 25 °C and AFM images were collected after 48 h. Dotted and solid boxes highlight reactants and products, respectively. Colons in the species names indicate multi-tile complexes. Scale bars are 200 nm

In a DNA strand displacement reaction, a single strand with a toehold domain binds to an uncovered complementary domain in a double-stranded complex, initiates a branch migration process, and eventually releases the previously bound strand while becoming part of a double strand itself (Fig. 1a). Similarly, in a DNA tile displacement reaction, an invader tile with a toehold domain binds to a complex consisting of a cover tile and a base tile, initiates a competition with the cover tile for binding to the base tile, and eventually releases the cover tile while itself becoming fully bound to the base tile (Fig. 1b and Supplementary Movie 1). Unlike a DNA strand displacement reaction, the toehold and branch migration domains in a tile displacement reaction consist of a set of edge staples rather than a string of nucleotides (Fig. 1c and Supplementary Fig. 1f).

Each DNA origami tile is composed of four isosceles triangles, with bridge staples zipping together the seams between the adjacent triangles (Fig. 1c). To visualize the reactants and products of the desired reaction by atomic force microscopy (AFM), we labeled the invader tile with an X and the cover tile with an O using patterns of double-stranded staple extensions. In prior work, this origami design was used to scale up the diversity^24^ and complexity^11^ of two-dimensional DNA nanostructures. Here, we chose this origami design for exploring the full potential of tile displacement because it has a good degree of structural flexibility along all four edges to possibly allow branch migration within complex multi-origami structures—it is known that bending between adjacent helices^10,28^ and near the internal seams^24^ is possible. Moreover, the tiles can self-assemble into larger structures with a set of edge staples that each has a blunt-end stacking bond and a very short sticky end—weak enough to allow fraying between staple pairs.

### Kinetics of DNA tile displacement

The capability of controlling reaction kinetics, making some reactions faster or slower than the others, gives rise to complex behaviors in diverse chemical and biological systems, for example as seen in the transient memory in bacterial chemotaxis^29^ and the oscillations in cell cycles^30^. It is desirable to achieve controlled reaction kinetics in engineered systems, even if the overall behaviors only share similarities at the abstract principle level or are a lot simpler than those seen in biology. For example, in strand displacement circuits, controlled reaction kinetics has enabled dynamic behaviors including consensus^17^ and oscillation^18^. To identify the range of kinetics that can be controlled in tile displacement reactions, we created toeholds with varying strengths and performed a set of fluorescence kinetics experiments to measure the reaction rates.

While keeping the number of staples and the length of sticky ends in the branch migration domain the same, we varied the toehold domain from 0 to 4 staples each with 1- to 2-nucleotide sticky ends (Fig. 1d). For the convenience of experiments, we kept all 4 toehold staples in the base tile and only varied those in the invader. At the end of the branch migration domain, two paired staples were modified with a fluorophore and a quencher, respectively. While the cover tile remains bound to the base tile, the fluorophore will be quenched and result in low fluorescence signal. If the cover tile with the quencher is released, the fluorescence signal will consequently increase.

Within 24 h, the fluorescence trajectories essentially did not change over time for invaders with 0 toehold staples (Fig. 1f). With 1 to 4 toehold staples, a range of reaction kinetics was observed. Naturally, with the same length of sticky ends, more staples resulted in faster kinetics; with the same number of staples, longer sticky ends resulted in faster kinetics. Interestingly, toeholds with staples that have 1-nt and 2-nt sticky ends saturated at noticeably different rates.

To gain a quantitative understanding of the kinetics, we utilized a simple model to analyze the tile displacement reactions (Fig. 1e), of a similar mathematical form as was used for strand displacement reactions^20^. Comparing the simulations with experimental data (Fig. 1f), including additional experiments with varying concentrations of the invader (Supplementary Fig. 2), we found a set of parameters that explained the data reasonably well (Fig. 1e and Supplementary Note 1, Supplementary Eqs. (1) and (2)). In summary, the binding rate is roughly 10 to 100 times slower than strand displacement, depending on the length of the sticky end. Similar to strand displacement, the dissociation rate decreases exponentially with increasing number of nucleotides in the toehold, which depends on both the number of toehold staples and the length of the sticky end. The displacement rate is roughly 40 times slower than strand displacement. The effective rate of the overall tile displacement reaction reached a maximum of 4.5 × 10^5^ M^−1^ s^−1^. At low concentration (e.g., <50 nM), the bimolecular binding rate limits the overall reaction rate and an increasing concentration will result in faster tile displacement; at high concentration (e.g., >50 nM), the unimolecular displacement rate limits the overall reaction rate and an increasing concentration will become less significant and eventually saturate.

To compare the reaction completion levels from fluorescence kinetics experiments with AFM experiments, we analyzed the number of products over the total number of products and reactants in 5 by 5 μm images that contained on average more than 40 tile complexes. All samples were taken directly from the kinetics experiments and imaged at 48 h. We found 3.8 ± 0.5% and 96.2 ± 3.7% reacted tile complexes for invader without and with a 2-nt 4-staple toehold, respectively (Supplementary Fig. 3a). Similarly, 5.1 ± 0.8% and 93.0 ± 3.3% of tile complexes reacted with an invader without and with a 1-nt 4-staple toehold, respectively. The observations from both types of experiments were in agreement with each other.

### Competitive reconfiguration

**Fig. 2:**
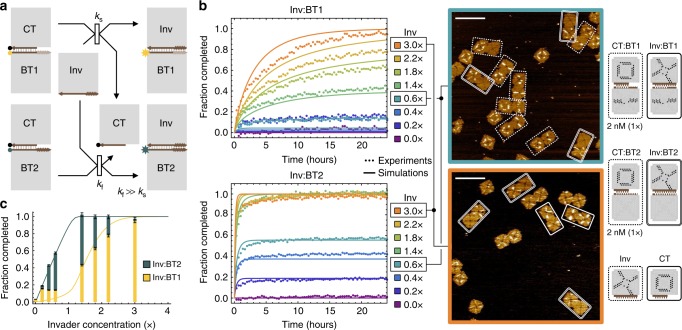
Competitive reconfiguration. **a** Domain-level diagram of two competing tile displacement reactions. BT1 and BT2 are base tiles with 1-nt and 2-nt sticky end in 4 toehold staples, respectively. They are labeled with two distinct fluorophores for the two reactions to be monitored simultaneously. *k*_s_ and *k*_f_ are effective rates of the two reactions, respectively. **b** Fluorescence kinetics experiments, simulations, and AFM images of competitive tile displacement. 1× corresponds to a standard concentration of 2 nM. Dotted and two types of solid boxes highlight reactants and two types of products, respectively. Scale bars are 200 nm. **c** Completion level of competitive tile displacement at 24 h (bars) overlaid with simulations (lines). It is clear from the kinetics trajectories shown in **b** that the completion levels for Inv:BT2 with invader concentration from 1.4× through 3.0× have reached maximum reaction completion, and that the difference in these completion levels reflects the noise in the experimental measurements. Thus, error bars correspond to the standard deviation of these four completion levels, scaled proportionally to each of the 16 completion levels

With the ability to control kinetics over five orders of magnitude, it is now possible to create system-level behaviors that exploit the rate differences among multiple tile displacement reactions. For example, competition can be created to allow a basic type of information-based structural reconfiguration: a sigmoidal function in response to a signal concentration. This function is one of the essential building blocks for digital logic computation^15^ in strand displacement and other synthetic circuits. To demonstrate this function, we designed two competing tile displacement reactions triggered by the same invader, one at a much faster rate than the other (Fig. 2a). The invader tile has a 2-nt 4-staple toehold, expected to interact with two types of cover:base tile complexes, one with a matching toehold and the other with a 1-nt 4-staple toehold. For the latter, based on the kinetics measurements, the effect of a single dangling nucleotide should be insignificant here. We expect the overall rate of the faster reaction to be approximately 18 times that of the slower reaction (Supplementary Fig. 2). Two distinct fluorophores were used to monitor the two products simultaneously.

When the invader concentration was less than 2 nM (1×), it preferentially triggered the faster reaction and the concentration of the product from the slower reaction remained low (Fig. 2b). However, when the invader concentration exceeded 1×, the faster reaction was quickly saturated and the excess invader was available to also trigger the slower reaction. With the same model and rate constants shown in Fig. 1e (Supplementary Eqs. (3) and (4)), we were able to predict the kinetics of the competition reasonably well. Looking at the completion levels of the two competing reactions at 24 h, the product of the faster reaction increased linearly in response to the invader concentration, until reaching its maximum, while the product of the slower reaction exhibited a sigmoidal function (Fig. 2c). The faster reaction functioned as a threshold for the slower reaction. By tuning the concentration of the cover:base tile complex in the faster reaction, one can in principle tune the value of the threshold and thus shift the sigmoidal function as desired.

We chose two representative samples with an invader concentration below and above the threshold to visualize the reconfiguration results using AFM (Fig. 2b). In the first case, most tile complexes were unreacted with a cover tile (labeled with an O) bound to the base tile (labeled with a line for one type and left unlabeled for the other). Largely, only one type of product from the faster reaction was observed, in which the cover tile was replaced by an invader labeled with an X. In the second case, nearly no unreacted tile complexes were present and both types of reconfigured products were found. Using 5 by 5 μm images that contained at least 90 tile complexes, the percentage of reactants that were converted to products for the slower and faster reconfiguration pathways were quantified to be 8.7 ± 0.8% and 57.0 ± 3.6%, respectively, with 0.6× invaders, and 97.7 ± 2.3% and 100%, respectively, with 3× invaders (Supplementary Fig. 3b). Again, the results were consistent between AFM and fluorescence kinetics experiments.

### Sequential reconfiguration

**Fig. 3:**
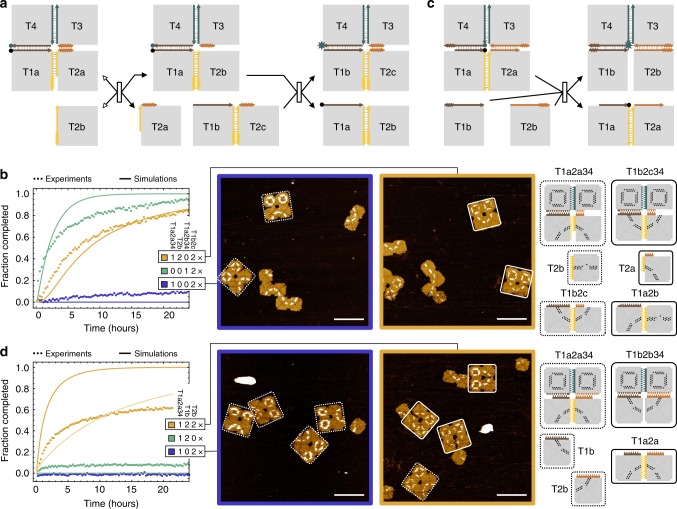
Sequential and cooperative reconfiguration. **a** Domain-level diagram of two reactions that take place in a cascade to first displace a single tile and then displace two tiles from a 2 by 2 array. Toehold and branch migration domains with unique sets of edge staples are in distinct colors. Arrows with black-filled and white-filled arrowheads indicate the forwards and backwards directions of a reaction step, respectively. **b** Fluorescence kinetics experiments, simulations, and AFM images of sequential tile displacement. Species names of multi-tile complexes are abbreviated by omitting colons and repeated Ts (e.g., T1a2a34 indicates T1a:T2a:T3:T4). Some structures in the AFM images are spurious dimers of invaders (T1b2c) or products (T1a2b), due to non-specific binding of the stacking bonds or sequence similarity of the sticky ends. Imperfect stoichiometry in assembling the reactants leads to small excess of some tiles, which could further introduce aggregates between structures with active edges. A few example diagrams for interpreting these spurious structures are shown in Supplementary Fig. 5. **c** Domain-level diagram of two invader tiles cooperatively displacing two tiles from a 2 by 2 array. **d** Fluorescence kinetics experiments, simulations, and AFM images of cooperative tile displacement. In simulations, the lighter yellow trajectory corresponds to a model that includes invader dimerization. The standard concentration (1×) in all experiments was 2 nM. In all AFM images, dotted and solid boxes highlight reactants and products, respectively. Scale bars are 200 nm

Next, we explored if the mechanism of toehold sequestering can be implemented in tile displacement to allow for cascades of structural reconfigurations and thus more sophisticated system behaviors. We designed a 2 by 2 array of tiles in which a first invader can displace one tile while revealing a previously protected toehold, and sequentially a second invader consisting of two tiles can displace two other tiles from the same array (Fig. 3a). The fluorophore and quencher were placed near the end of the second branch migration domain to monitor the completion of the two-step reconfiguration. From initial experiments, we learned that displacement across tile corners could be slow and intermediate states of displacement should be considered for possible spurious reactions (Supplementary Fig. 4 and Supplementary Note 2). With this understanding, the design shown in Fig. 3a ensures that spontaneous dissociation only occurs for toehold domains and no intermediate states should lead to any undesired products.

The fluorescence kinetics experiments showed that with both invaders, the two-step reconfiguration successfully took place (Fig. 3b, yellow trajectory). As expected, the two-step cascade was slower than the second step alone (green trajectory). Lacking the first invader, the second invader alone triggered only a small fraction of spurious reactions (blue trajectory). We revised the model to include a branch migration step across the corners of adjacent tiles (Supplementary Eqs. (5) and (6)). Comparing the data with simulations, we estimated that the displacement rate across corners is roughly 100 times slower than that within the same tile edge.

AFM experiments confirmed that the 2 by 2 arrays remained unreacted, labeled as a frown, with the second but not the first invader, and reconfigured into a smile with the presence of both invaders (Fig. 3b and Supplementary Fig. 5a). With increased complexity of the reactants, imperfect stoichiometry of tiles in the self-assembly process and spurious interactions between tiles with active edges become more significant and thus interpreting the structures in AFM images become more challenging. Using the two types of products (a 2 by 2 array and a two-tile complex), the yield of sequential tile displacement (i.e. the percentage of reactants that were converted to products) was estimated as 83.3 ± 9.8% and 90.5 ± 6.1% at 48 h, respectively. The difference of the two estimates is within the statistical error, indicating that the estimates are not perfectly accurate but still reasonable.

The sequential tile displacement system not only demonstrated the principle of toehold sequestering and cascades, but also enabled another basic type of information-based structural reconfiguration: response to more than one environmental signal that indicate given instructions or available resources, represented as two types of invaders. Functionally, the first invader could arrive much earlier than the second and the structure would still be reconfigured as expected. In the next example system, we ask: can structural reconfiguration be programmed to take place only when two types of signals are simultaneously present?

### Cooperative reconfiguration

Similar to the principle of cooperative hybridization^31^, we designed two invader tiles that each bind to one side of a 2 by 2 array (Fig. 3c). If only one tile is present, it should branch migrate to the center of the array. However, without a second tile, the process should be reversible and the invader will dissociate again. When both tiles are present, the two branch migration processes should meet at the center of the array, resulting in a cooperative tile displacement. Because it is effectively a trimolecular reaction with all three reactants at a relatively low concentration, for the overall reaction to take place at a reasonable rate, the binding and branch migration should be sufficiently fast and the dissociation should be sufficiently slow. With these considerations, we chose a 2-nt 3-staple toehold for each invader (Fig. 3d), which was shown to be almost as fast as a 2-nt 4-staple toehold in the kinetics experiments but also reversible enough to allow for toehold dissociation.

In fluorescence kinetics experiments, when the 2 by 2 array was mixed together with one or the other invader, the fluorescence signal remained low (Fig. 3d, blue and green trajectories). But when both invaders were present, the signal went high (yellow trajectory). We simulated the cooperative reactions with all possible binding, dissociation, and displacement steps (Supplementary Eqs. (7) and (8)). Despite that the completion level was different, the half completion time of the experiment roughly agreed with the simulation. In AFM experiments, the 2 by 2 arrays remained unreacted (labeled as a frown) with only one invader, but 68.0 ± 7.7% of them reconfigured into a smile with both invaders (Supplementary Fig. 5b). We observed a higher fraction of invaders that spuriously formed dimers in these AFM images, suggesting a possible explanation for the lower completion level. By adding the dimerization reactions into the model (Supplementary Eq. (9)), the completion level in simulation better agreed with the experiment (Fig. 3d, lighter yellow trajectory).

### Generality of DNA tile displacement

With the three example systems, we have demonstrated two key properties of DNA tile displacement reactions as well as using them for information-based autonomous reconfiguration in systems of multiple interacting DNA origami structures. The properties of controlled kinetics with varying toehold strengths and cascades through toehold sequestering, which have enabled sophisticated dynamic behaviors in DNA circuits and robots, are now proven to exist at a much larger scale in complex DNA nanostructures. However, to what extent tile displacement reactions can be used to create increasingly powerful system behaviors depends on the generality of these reactions for taking place at any location in an array.

**Fig. 4:**
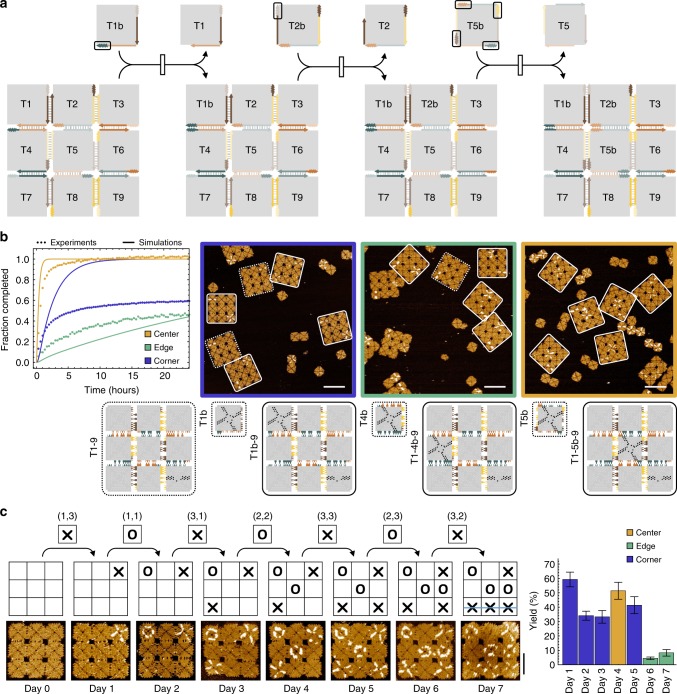
Generality of DNA tile displacement. **a** Three types of tile displacement reactions, shown in a cascade, in 3 by 3 arrays. 12 toehold and 12 branch migration domains with unique sets of edge staples are in distinct colors. Toeholds used to initiate each reaction are highlighted in boxes. **b** Fluorescence kinetics experiments, simulations, and AFM images of three example reactions, one of each type, performed separately. All three reactions have the same array as a reactant at 4 nM and distinct invader tiles at 8 nM. In AFM images, dotted and solid boxes highlight reactants and products, respectively. Species names of multi-tile complexes are further abbreviated by replacing continuous tile numbers with a dash (e.g., T1-9 indicates T123456789). Scale bars are 200 nm. **c** Design diagram, AFM images, and yield estimation of a tic-tac-toe game. Two players each have 9 tiles labeled with X or O, each of which is designed to displace one specific tile from the array. (*x*, *y*) indicates the position of the tile to be displaced is in row *x* and column *y*. Scale bar is 100 nm. Yield was estimated as the number of desired products (shown in the design diagram and representative AFM image for each day) divided by the total number of all 3 by 3 arrays in 10 by 10 μm AFM images. The standard error was calculated as $p\sqrt {1 - p} {\mathrm{/}}\sqrt n$, where *p* is the estimated yield and *n* is the total number of arrays, treating the yield as a Bernoulli probability. *n* = 54, 82, 39, 37, 29, 22, and 12 for days 1 through 7. Error bars correspond to the standard error of the yield

To explore this generality, we designed a 3 by 3 array that allows nine unique tile displacement reactions to take place in any desired order (Fig. 4a). There are three types of reactions: displacing a corner tile, an edge tile, and a center tile, which complete the set of possible reactions for displacing a tile with any number of neighbors in arrays of any size. Along the exterior of the array, we placed one toehold between any two adjacent tiles, resulting in 8 unique toeholds. Half of them were used to initiate a corner tile displacement, where an invader with the matching toehold binds to the edge tile adjacent to the corner tile, branch migrates within a tile edge and then across a 90 degree corner, releasing the previously bound corner tile and integrating itself into the array. The other half of the toeholds were used to initiate an edge tile displacement. These toeholds are present both in the original corner tiles and their invaders. Thus, regardless of whether the adjacent corner tile has been displaced yet, an edge tile invader can bind to the matching toehold, branch migrate through three tile edges across two corners, and release the previously bound edge tile.

Considering that it may be difficult to initiate a center tile displacement because of limited toehold accessibility near the interior of an array, 4 additional toeholds between the center tile and its neighbors were used to collectively initiate the reaction (Fig. 4a). Similar to the other types of reactions, these toeholds are also present both in the original edge tiles and their invaders. With 4 toeholds on a center tile invader, any of the toeholds can bind to an edge tile and branch migrate to disconnect one edge of the center tile. When all four edges of the original center tile have been disconnected, it will be fully displaced by the invader.

To create 12 unique toeholds and branch migration domains, we reduced the number of staples in a toehold but increased the number of nucleotides in each sticky end. With 5-nt 2-staple toeholds, whose strength should be similar to 2-nt 4-staple toeholds, 9 staples are now available for creating coded branch migration domains in which specific sets of edge staples are left out. We designed three unique edge codes, each consisting of 6 staples (Supplementary Fig. 7a).

We modeled the three types of tile displacement reactions, considering the branch migration steps within a tile edge and across a tile corner (Supplementary Eqs. (10) and (11)). Comparing the simulations with experimental data, we estimated that the displacement rate for coded branch migration domains is roughly 25 times slower than that for branch migration domains with continuous edge staples. As predicted by simulations, fluorescence kinetics data showed that the center tile displacement was the fastest and the edge tile displacement was the slowest (Fig. 4b). A simple explanation is that the overall reaction rate depends on the total number of branch migration steps across a tile corner, which is the slowest reaction step for completing a displacement. Unlike what the simulations predicted, the completion level of the corner tile displacement is much lower than that of the center tile displacement. We attribute it to the impurity of the molecules: similar to how synthesis errors in unpurified DNA strands could significantly affect the completion level of a strand displacement circuit^32^, given the complexity of the molecules, tile displacement reactions could be even more prone to synthesis errors. Moreover, missing staples^33,34^, especially those in the toeholds, could also significantly affect the completion level of tile displacement reactions. Thus, the fraction of arrays that are fully reactive—those with neither synthesis errors in edge staples nor missing edge staples—would decrease quickly with an increasing number of branch migration domains per toehold. This hypothesis is reflected in the order of completion levels of the center, corner, and edge tile displacement: it agrees with the number of toeholds per branch migration domain—1, 1/2, and 1/3, respectively.

With a unique label on a corner tile, AFM experiments confirmed that all three types of invaders were incorporated into the designed locations in the array, and the percentage of reactants that were converted to products was estimated as 78.4 ± 6.0%, 52.8 ± 6.0%, and 100% for the corner, edge, and center tile displacement, respectively (Supplementary Fig. 6).

With an understanding of the three types of tile displacement reactions, we proceeded with a tic-tac-toe game that demonstrated all 9 possible reactions in cascades. In this game, the 3 by 3 array with all original plain tiles was used as a nanoscale game board (Supplementary Fig. 7a). Each of the two players was given 9 invader tiles labeled with an O or X. Making a move simply corresponded to adding a tile to the test tube containing the game board. Each subsequent move was made after 24 h. Representative AFM images showed that the game board was piece-by-piece reconfigured in response to the signals given by the players, in all three games that we played (Fig. 4c and Supplementary Fig. 7d, e). The yield of the correctly reconfigured arrays incorporating all target moves mostly decreased with an increasing number of moves. However, because the desired reactions continue to approach completion after 24 h, if the new move is fast enough, some arrays could incorporate a previous move and a current move within the same day, recovering the yield. This was seen when a center piece was played. By the end of the game, the yield was estimated to be 8.3 ± 2.3% (Fig. 4c). Because the number of possible distinct arrays increases quickly with the number of moves played (Supplementary Fig. 8) and spurious interactions between all structures also increase quickly with an increasing excess of invaders and displaced monomers, the analysis became much rougher toward the end of the game (Supplementary Figs. 9–15).

## Discussion

We have shown that tile displacement is a simple-yet-powerful mechanism for programming dynamic behaviors in systems of interacting DNA nanostructures. A few challenges need to be addressed for scaling up tile displacement systems, including the yield of DNA origami arrays before displacement (Supplementary Fig. 16), aggregation of invaders, and spurious reactions involving intermediate products (Supplementary Discussion). Nonetheless, in principle, displacement allows for tiles at any desired locations in larger arrays to be reconfigured (Supplementary Fig. 17a). Increasing the number of toeholds along each tile edge could lead to faster reaction kinetics and higher completion levels (Supplementary Fig. 17b). With stacking bonds and sticky end sequences fully dependent on the M13 scaffold sequence, there is not enough specificity within the branch migration domains when the edge staples are continuous (Supplementary Fig. 17c). However, this specificity can be increased by using coded edges (as shown in Fig. 4b), sticky ends with varying lengths, and different combinations of extensions and truncations at both ends of the staples. Furthermore, extended edges^24^ could be used to remove the sequence dependence and create a much larger library of unique toehold and branch migration domains with more precisely controlled binding energies. Importantly, there is already enough specificity in the toeholds (Supplementary Fig. 17c) that could allow for reconfiguration of DNA origami arrays even if the tiles have common edges for binding to each other and the arrays are assembled using hierarchical approaches^11^.

In this work, tile displacement experiments were performed at a much lower concentration compared to typical strand displacement systems (2 nM vs. 100 nM). Using a mass-production method^35^, DNA origami structures could be assembled at a much higher concentration and a much lower cost, making tile displacement systems much faster for practical applications.

Conceptually, tile displacement provides a new understanding of how complex molecular structures could interact with each other and how system-level reconfiguration behaviors could occur. Along the tile edges within a two-dimensional (2D) structure, square or any other shape, information can be encoded into several functionally independent domains. Depending on which domains are protected and which are revealed, the structure could interact with various other structures and exchange which ones they are bound to at different times. The dynamic interactions between 2D structures directed by these “smart edges” could be extended to that between 3D structures, especially the flexible ones^36,37^, directed by “smart surfaces” with information-bearing 2D domains, resulting in more general forms of “structure displacement”. Compared to the previously known binding and unbinding interactions between complex DNA nanostructures, displacement allows isothermal reconfiguration in non-equilibrium structures and thus much more interesting dynamic behaviors with lower energy barriers.

Practically, tile displacement provides several unique advantages for controlling structural reconfiguration, including efficiently swapping in and out complex functional components that are pre-fabricated on DNA origami surfaces, programming cascades of autonomous reconfiguration events within a network of interacting complex molecular structures, and creating parallel reconfiguration behaviors unique to the information embedded within each molecular structure (Supplementary Discussion).

In general, the ability to swap out any desired structural components at the right time, together with the molecules attached to their surfaces, will allow artificial molecular machines to adapt their functions in response to the molecular environment during autonomous operation (Supplementary Fig. 18). In this environment, different instructions from other machines and resources may be presented at different times, requiring reconfiguration decisions to be made accordingly. Integrating tile displacement with strand displacement circuits for sensing small molecules and proteins using aptamers^38^ could allow reconfiguration to be triggered by more diverse environmental signals. Integration could also allow more sophisticated information processing, for example as shown by the classic deoxyribozyme-based automaton that plays tic-tac-toe^39^, to direct structural reconfiguration (Supplementary Discussion). The principle of reconfiguration-enabled adaptive behaviors in biology, epitomised by how membrane proteins on cell surfaces are swapped out to alter cell states during development and learning^40^, inspires a possible future application of tile displacement: information-based structural reconfiguration could be employed on the surface of an artificial cell to empower it with intelligent and responsive behaviors.

## Methods

### Edge staple design

Each edge staple that is not in a toehold has one stacking bond and one 2-nt sticky end. These staples hold the adjacent tiles together in an array, and some of them are in a branch migration domain. In order to create a large set of unique binding or branch migration domains, they may be encoded by using a subset of edge staples. To promote domain continuity, no more than 2 staples may be absent in a row.

Each toehold staple has one stacking bond and one 1-, 2-, or 5-nt sticky end. All toeholds on invader tiles have nucleotide extensions (“giving” staples), whereas the complementary toeholds have nucleotide truncations (“receiving” staples). Generally, the 5′ ends of the edge staples are extended for giving staples, and the 3′ ends are truncated for receiving staples. The inverse may also be true when more toeholds with unique sticky end sequences are desired such as in the tic-tac-toe experiments. Up to four edge staples may be involved in a single toehold.

The sequences of the sticky ends in the edge staples depend on the sequence of the M13 scaffold strand and thus interactions between different edges of a tile are naturally different due to the variation of M13 sequence along the edges of the tile. Taking advantage of this property, up to four unique edge interactions (including toehold and branch migration domains) can be created even when the locations of edge staples and the lengths of their sticky ends are the same. It may be possible to utilize an extended edge design^24^ as a more general approach for increasing the design space of edge interactions, which could potentially be used for creating a large set of distinct branch migration and toehold domains.

### DTD Designer

An online software tool, the DTD Designer^41^, was developed to facilitate the design of tile displacement systems. It assists a user via a graphical user interface in defining a set of square DNA origami tiles, including their edge staple design. Patterns designed using the FracTile Compiler^42^ can be applied to individual tiles. The final layout may be compiled into DNA sequences, experimental protocols, and a mixing scheme that can be read by an Echo 525 liquid handler to automatically mix all DNA strands for constructing each tile.

### Sample preparation

The scaffold strand was single-stranded M13mp18 DNA (Bayou Biolabs, catalog # P-107), supplied in 1× TE buffer (10 mM Tris-HCl, 1 mM EDTA, pH 8.0). The concentration of the scaffold was measured using NanoDrop2000 (Thermo Scientific) based on absorbance at 260 nm. Staple strands (sequences listed in Supplementary Tables 1–4) and negation strands (sequences listed in Supplementary Table 5) were purchased from Integrated DNA Technologies. Fluorophore and quencher modified staple strands were purchased with HPLC purification and all other strands were purchased with no purification (standard desalting). Staple strands were stored at 100 μM and negation strands at 300 μM in 1× TE buffer in Echo qualified 384-well source microplates (Labcyte).

To prepare individual DNA origami tiles, 50 nM scaffold strand, 50 nM staples with fluorophores and quenchers, and 250 nM all other staples were mixed together in 1 × TE/Mg^2+^ buffer (1× TE with 12.5 mM Mg^2+^) using an Echo 525 liquid handler (Labcyte), transferred from source plates into a 96-well destination plate (Eppendorf, catalog # 951020401). The typical volume of each sample was 40 μL. The destination plate was centrifuged at 2,000 rcf for 1 min, and the samples were then transferred into PCR tube strips (Eppendorf, catalog # 951010022). Annealing of the DNA origami tiles was performed by keeping the samples at 90 °C for 2 min and then cooling down from 90 to 20 °C at 6 s per 0.1 °C on a Nexus Mastercycler (Eppendorf). After the anneal, a five-fold excess (relative to the concentration of the staple strands) of a full set of 44 negation strands were added to each type of DNA origami tile and quickly cooled down from 50 to 20 °C at 2 s per 0.1 °C.

The cover:base tile complexes (shown in Figs. 1f and 2b) were prepared by adding 20% excess of the cover tile. All other origami arrays were prepared by mixing equal volumes of individual tiles. All origami arrays were annealed from 50 to 20 °C at 2 min per 0.1 °C.

### Echo protocol

The transfer volume in a protocol for an Echo 525 liquid handler must be multiples of 25 nL. Additionally, the volume of sample in each well of the Echo qualified 384-well source plate must be 15–65 μL, resulting in 15 μL of unusable sample. Because of both constraints, we diluted the fluorophore staples to 20 μM and quencher staples to 40 μM before storing them in a source plate. All other edge staples and internal staples were at 100 μM. This resulted in a transfer volume of 25 nL for each edge and internal staple to have the 250 nM target concentration per 10 μL of final volume.

Because the bridge staples are the same in all tiles, we mixed them together and divided the mixture into five wells in a source plate. The concentration of the bridge staple mixture was at 100/38 = 2.63 μM, for 38 distinct bridge staples. A volume of 950 nL was transferred for a target concentration of 250 nM per 10 μL of final volume.

The concentration of the M13 scaffold varied from batch to batch, but typically the difference is no more than 10%. We used 0.333 μM of M13 divided into twelve wells in the source plate. The total transfer volume was 1.5 μL per 10 μL of final volume for a target of 50 nM.

We used eight wells of 1 × TE/10 × Mg^2+^, resulting in a transfer volume of 125 nL per well per 10 μL of final volume. We used sixteen wells of 1 × TE, transferring as evenly as possible as needed. The variable volume of 1 × TE is due to the fact that the total number of edge staples varies in different tiles.

### Fluorescence spectroscopy

Fluorescence kinetics data were collected every 3 or 4 min using a microplate reader (Synergy H1, Biotek) over 48 h. Experiments were performed at 1× = 4 nM for the tic-tac-toe experiments shown in Fig. 4 and Supplementary Fig. 7 and 1× = 2 nM for all other experiments. Measurements were made in 384-well plates (Corning # 3544) with 45 μL reaction mixture per well. The initial concentrations of all arrays were 1× and those of all invader tiles were 2×, unless otherwise specified in the figures. Excitation and emission wavelengths were 588 and 608 nm for ROX, and 549 and 563 nm for TYE563.

### AFM imaging

After being diluted to 1 nM (scaffold concentration) in 1 × TE/Mg^2+^ buffer, 40 μL of each sample was deposited onto freshly cleaved mica (SPI Supplies, catalog # 01873-CA). The solution was removed after 30 s, the mica surface was washed three times with 40 μL TE buffer containing 10 mM MgCl_2_ and 100 mM NaCl, and 80 μL of 1 × TE/Mg^2+^ buffer was then added before imaging. Imaging was done in fluid using FastScan-D probes (Bruker) and tapping mode on a FastScan Bio (Bruker), typically at a scan rate of 5 Hz with 1024 lines per image. The amplitude setpoint was usually between 30 and 50 mV, with drive amplitude at 180 to 240 mV and drive frequency at 110 Hz. The integral and proportional gains were set to 1 and 2, respectively. Samples that were not imaged immediately after the fluorescence kinetics experiments were kept at −20 °C and thawed before AFM imaging.

### Data normalization

All fluorescence kinetics data were normalized from raw fluorescence level to the fraction of reactions completed. Each set of experiments was performed with a negative and a positive control. The negative control was the initial structure (cover:base tile complexes or larger DNA origami arrays) without any invader tiles. The positive control was the target structure, in which the invader tile(s) were annealed together with the other tile(s) that were not to be displaced. The first 5 data points of the negative control and the last 5 data points of the positive control were averaged to determine the fluorescence levels that correspond to 0 and 100% of reactions completed, respectively. Occasionally, due to experimental noise such as inaccurate concentrations and volumes, the first (or last) 5 data points of a measured tile displacement reaction were below (or above) the negative (or positive) control. In those cases, the 0% (or 100%) level was determined using the smaller (or larger) averaged value.

### Code availability

Simulation code is available upon request to the corresponding author.

## Supplementary information

Supplementary Information

Description of Additional Supplementary Files

Supplementary Movie 1

## Data Availability

All data supporting the findings of this study are included in the manuscript and its Supplementary Information.
