# Author Correction: Information-based autonomous reconfiguration in systems of interacting DNA nanostructures

**DOI:** 10.1038/s41467-019-08439-z

**Published:** 2019-01-22

**Authors:** Philip Petersen, Grigory Tikhomirov, Lulu Qian

**Affiliations:** 1 Biology, California Institute of Technology, Pasadena, CA 91125 USA; 2 Bioengineering, California Institute of Technology, Pasadena, CA 91125 USA; 3 Computer Science, California Institute of Technology, Pasadena, CA 91125 USA

Correction to: *Nature Communications*; 10.1038/s41467-018-07805-7; published online: 18 December 2018

The original version of this Article omitted a reference to previous work in ‘Stojanovic, M. N. & Stefanovic, D. A deoxyribozyme-based molecular automaton. *Nat. Biotechnol*. **21**, 1069–1074 (2003)’. This has been added as reference 42. The following has been added after the third sentence of the fifth paragraph of the Discussion: ‘Integration could also allow more sophisticated information processing, for example as shown by the classic deoxyribozyme-based automaton that plays tic-tac-toe^42^, to direct structural reconfiguration (Supplementary Discussion)’. This has been corrected in the PDF and HTML versions of the Article.

