# Supplementary material for: Information-based autonomous reconfiguration in systems of interacting DNA nanostructures
Source: Nat Commun. 2018 Dec 18;9:5362. doi: 10.1038/s41467-018-07805-7 (PMC6299139; doi:10.1038/s41467-018-07805-7)
Supplement: Supplementary file 1 — Supplementary Information [file 41467_2018_7805_MOESM1_ESM.pdf]

Information-based autonomous reconfiguration in systems of interacting DNA nanostructures

Petersen et al.

Supplementary Figures

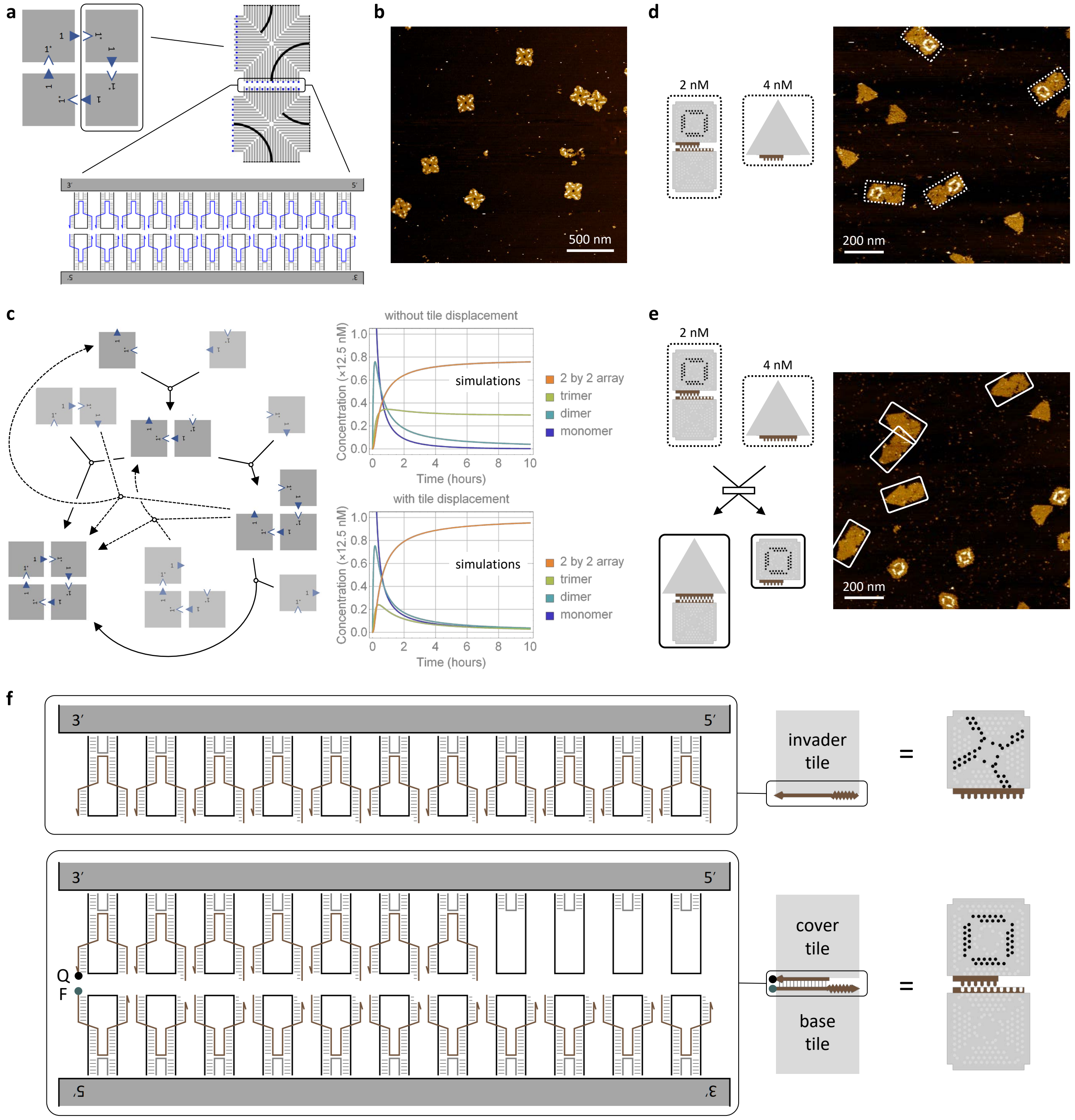

Supplementary Figure 1 | Discovery of DNA tile displacement. (**a**) A 2 by 2 array design that uses one tile with 11 edge staples along two edges. Each staple has a stacking bond and a 1-nt sticky end. (**b**) AFM image of the 2 by 2 array, with 50 nM of the tile annealed from 90 to 20 °C at 6 sec per 0.1 °C. (**c**) Possible self-assembly pathways without (solid arrows) and with (dotted arrows) tile displacement. Fainter tiles are duplicate and rotated representations of the same monomer, dimer or trimer. Simulations were performed with all possible reactions at the same effective rate $k = 2.5 \times 10^4\ \mathrm{M}^{-1}\mathrm{s}^{-1}$, which is the binding rate (and maximum effective tile displacement rate) for tiles with 1-nt sticky ends in the model shown in Fig. 1e. Simulations suggest that, if all bindings are irreversible, then without tile displacement, monomer depletion will prevent trimers from further converting to 2 by 2 arrays and lead to a significant fraction of kinetically trapped trimers at the end point. With tile displacement, a trimer could interact with a dimer to form a 2 by 2 array while releasing a monomer, and two copies of trimers could interact with each other to form a 2 by 2 array while releasing a dimer, resulting in nearly 100% of 2 by 2 arrays at the end point. (**d**) Design diagram and AFM image of a square dimer mixed together with an triangular invader[1] without a toehold and (**e**) with a toehold. Experiments were performed at 25 °C with 2 nM square dimer and 4 nM triangular invader. AFM images were collected after 48 hours. Dotted and solid boxes highlight reactants and products, respectively. (**f**) Edge staples composing the toehold and branch migration domains of a tile displacement reaction shown in Fig. 1c.

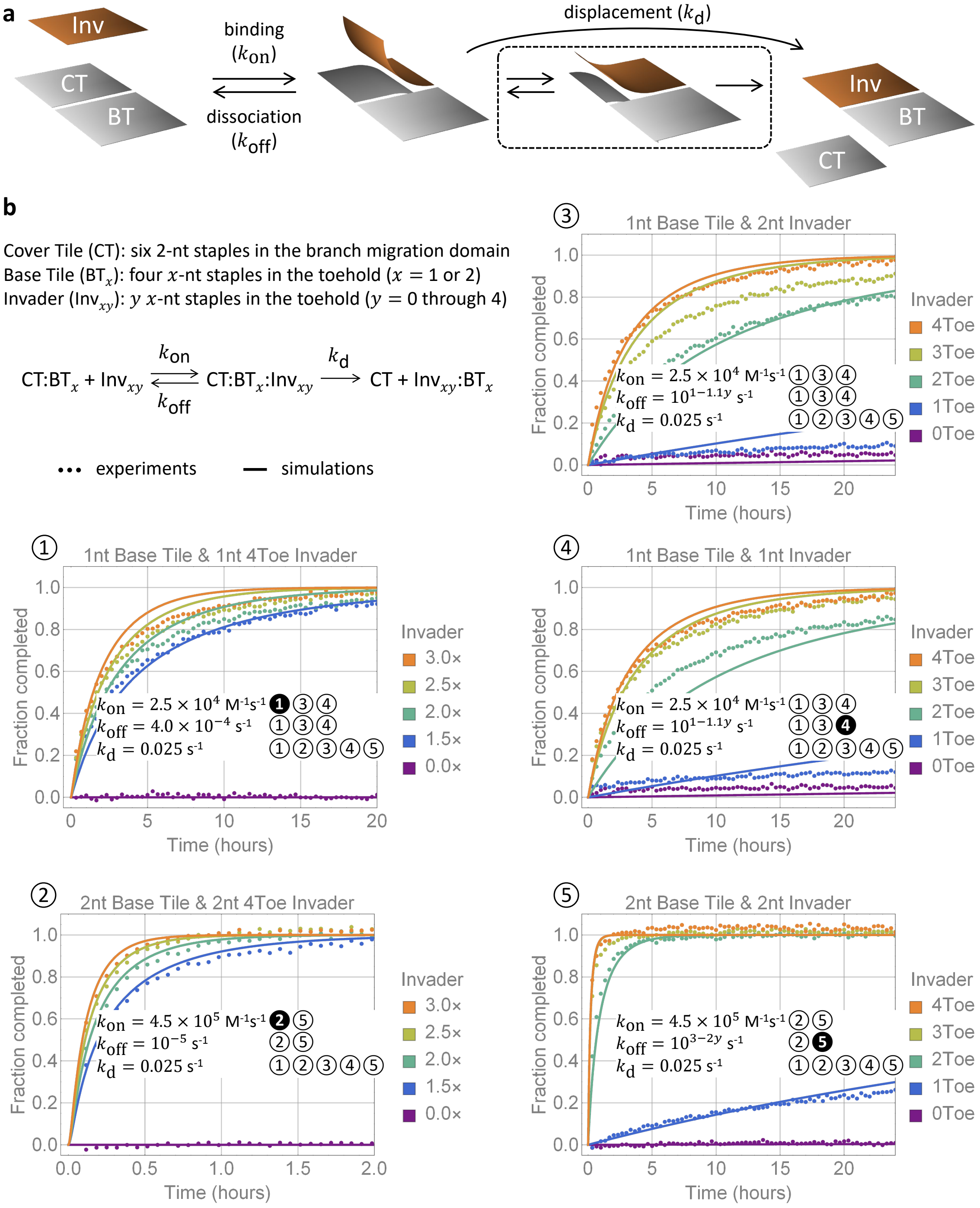

Supplementary Figure 2 | Kinetics of DNA tile displacement. (**a**) The pathway of a tile displacement reaction. Dotted box highlights an intermediate state of the displacement step. (**b**) Fluorescence kinetics experiments and simulations. Experiments were performed at 25 °C with 2 nM (1×) cover:base dimer and 4 nM (2×) invader (or otherwise specified invader concentrations in the plot legend). k_{on}, k_{off}, and k_{d} are the rates of binding, dissociation, and displacement, respectively. Numbers next to the rates indicate the plots for which each rate is associated with, and the highlighted numbers indicate the plot for which the rate was largely derived from.

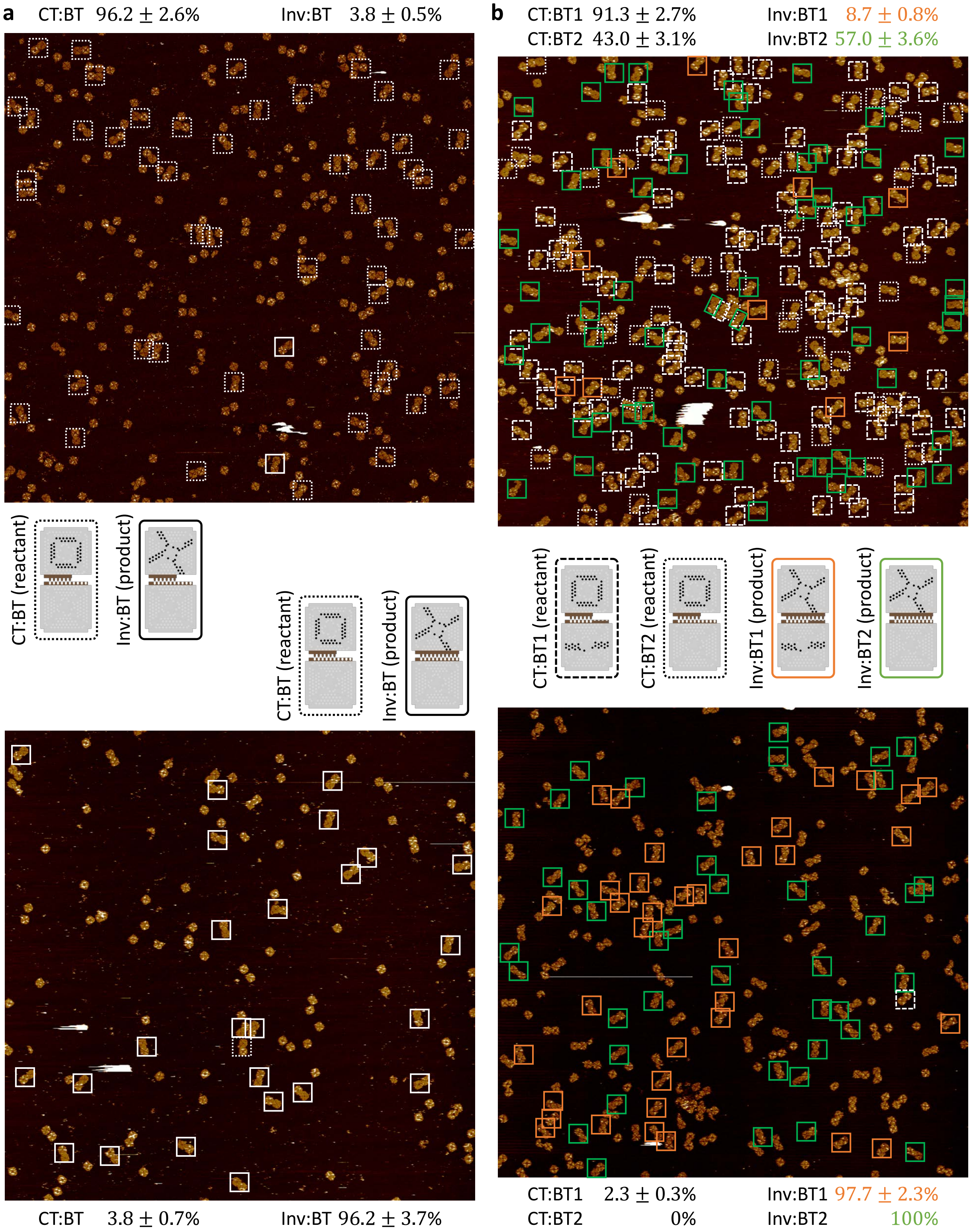

Supplementary Figure 3 | Yield estimation of basic and competitive tile displacement. (**a**) Cover:base tile complex mixed together with an invader without (top) and with (bottom) a toehold. (**b**) Two types of cover:based tile complexes mixed together with 0.6× (top) and 3× (bottom) invader. All AFM images are 5 by 5 μm. Each two-tile complex is highlighted as either a reactant (dotted boxes) or a product (solid boxes). The yield of each tile displacement reaction was estimated as $p = m/n$, where m is the total number of products and n is the total number of reactants and products counted in each image. Ambiguous and spurious structures were not counted. The standard error was calculated as $p\sqrt{1-p}/\sqrt{n}$, treating the yield as a Bernoulli probability.[2]

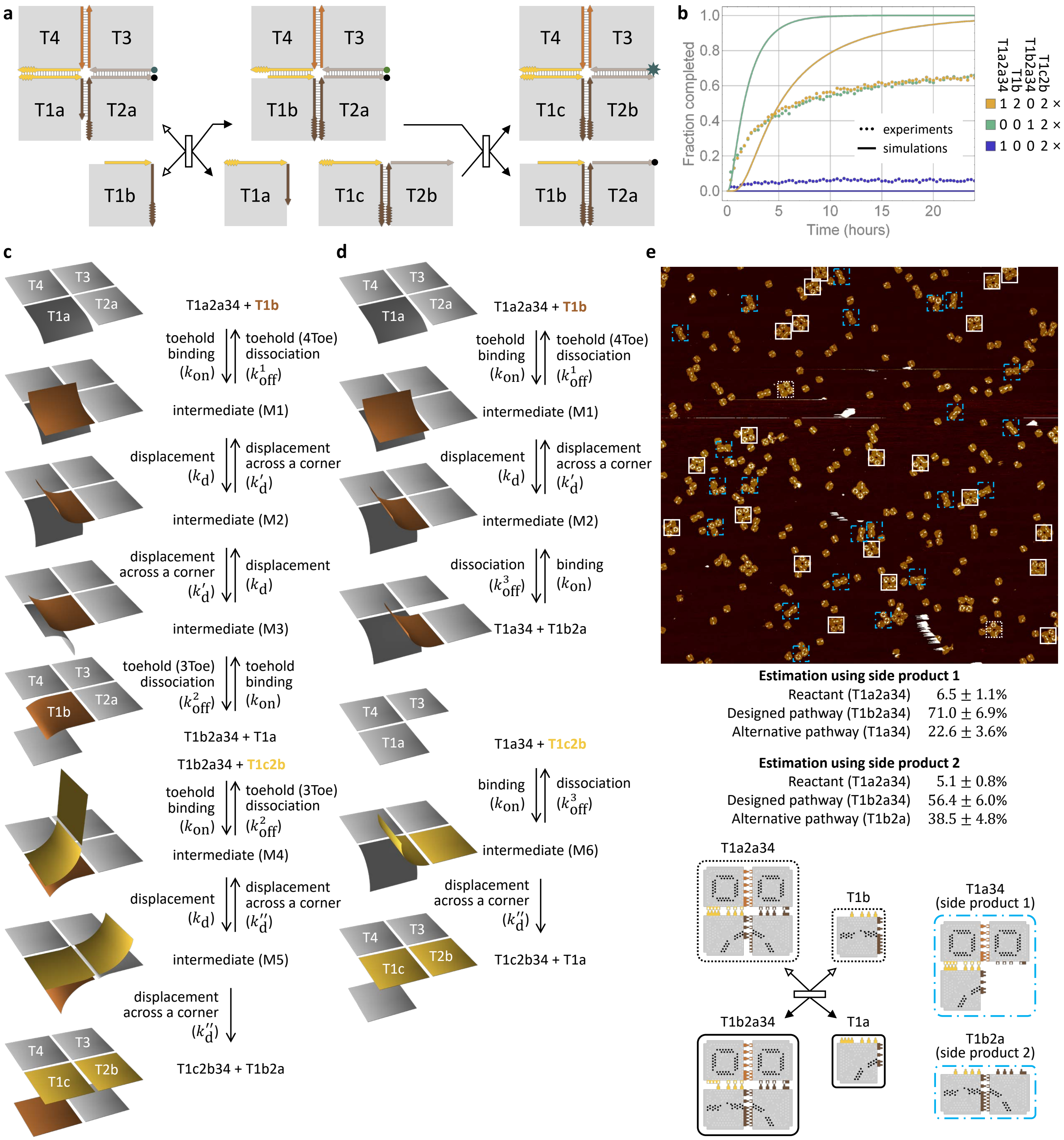

Supplementary Figure 4 | An earlier design of sequential reconfiguration. (**a**) Domain-level design diagram. (**b**) Fluorescence kinetics experiments and simulations. (**c**) Designed pathway. The first invader (T1b) displaces one tile from the 2 by 2 array, and then the second invader (T1c2b) displaces two tiles. (**d**) Alternative pathway. The first invader removes one tile from the 2 by 2 array, and then the second invader displaces another tile. (**e**) AFM image and yield estimation of the designed and alternative pathways in the first step of sequential tile displacement. The AFM image is 5 by 5 μm. Each 2 by 2 array is highlighted as either a reactant (dotted boxes) or a product (solid boxes). Two types of side products of the alternative pathway are also highlighted (dashed blue boxes) to provide two separate estimations of the yield. Using each type of side product, the yields of the designed pathway and alternative pathway were estimated as $p_1 = m_1/n$ and $p_2 = m_2/n$, respectively, where m_1 is the total number of products, m_2 is the total number of side products, and n is the total number of reactants, products, and side products. Ambiguous and spurious structures were not counted. The standard error was calculated as $p_i\sqrt{1-p_i}/\sqrt{n}$, treating the yield as a Bernoulli probability.

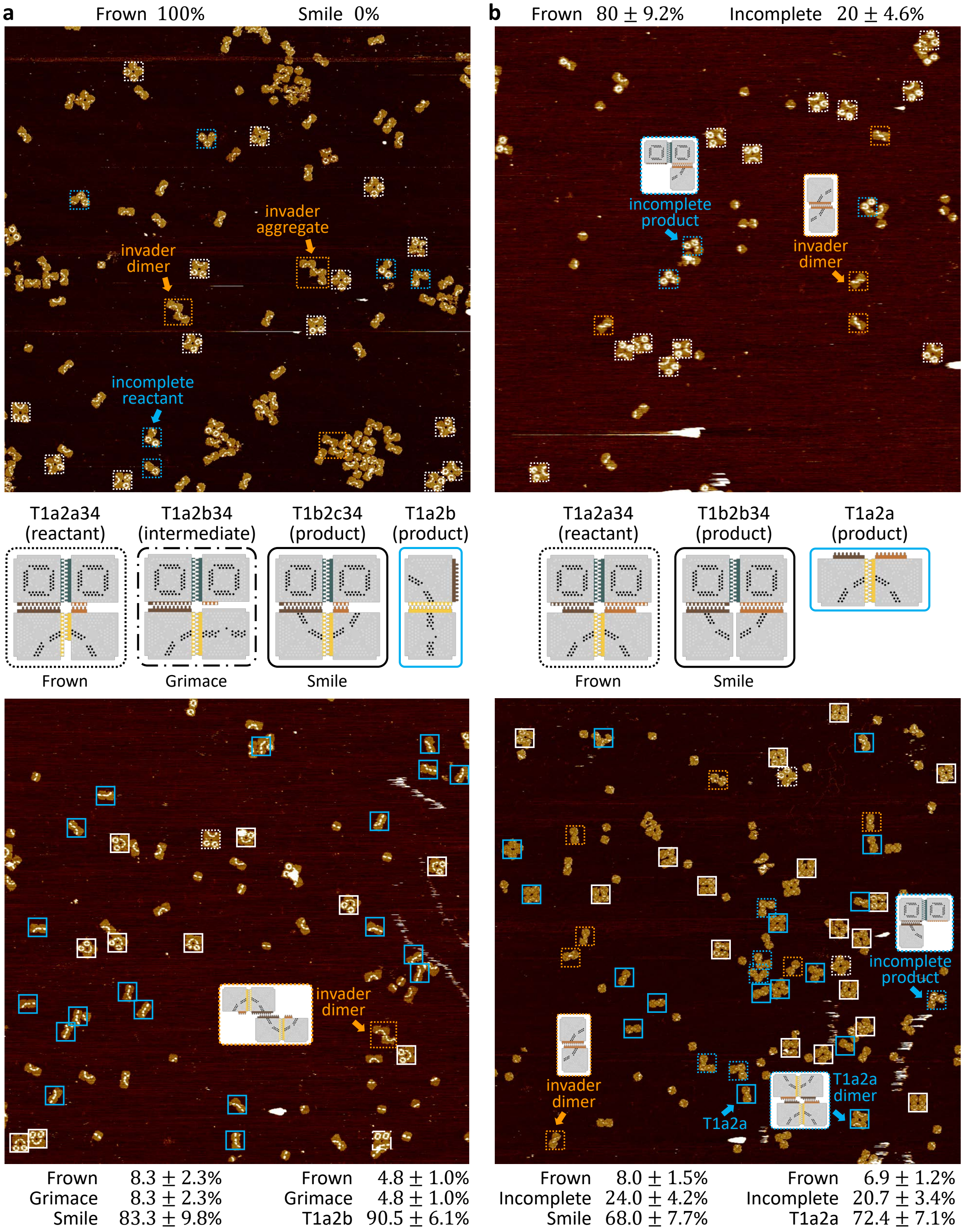

Supplementary Figure 5 | Yield estimation of sequential and cooperative tile displacement. (**a**) Sequential reaction in which 2 by 2 arrays were mixed together with the second invader without (top) and with (bottom) the first invader. (**b**) Cooperative reaction in which 2 by 2 arrays were mixed together with one (top) and both (bottom) invaders. All AFM images are 5 by 5 μm. Each 2 by 2 array is highlighted as either a reactant (dotted boxes) or a product (solid boxes). Incomplete reactants and products are highlighted in dotted blue boxes. In the bottom two images, the two-tile-complex products are also highlighted (solid blue boxes) to provide a second estimation of the yield. Invaders and products with active edges can form spurious dimers or even larger aggregates — a few examples of these spurious structures are highlighted with diagrams for interpreting the structures. The yield of each tile displacement reaction was estimated as $p_i = m_i/n_i$, where m_i is the total number of products ($i = 1$ for 2 by 2 arrays and $i = 2$ for two-tile complexes), and n_i is the total number of reactants, products, and incomplete products counted in each image. Ambiguous structures were not counted. The standard error was calculated as $p_i\sqrt{1-p_i}/\sqrt{n_i}$, treating the yield as a Bernoulli probability.

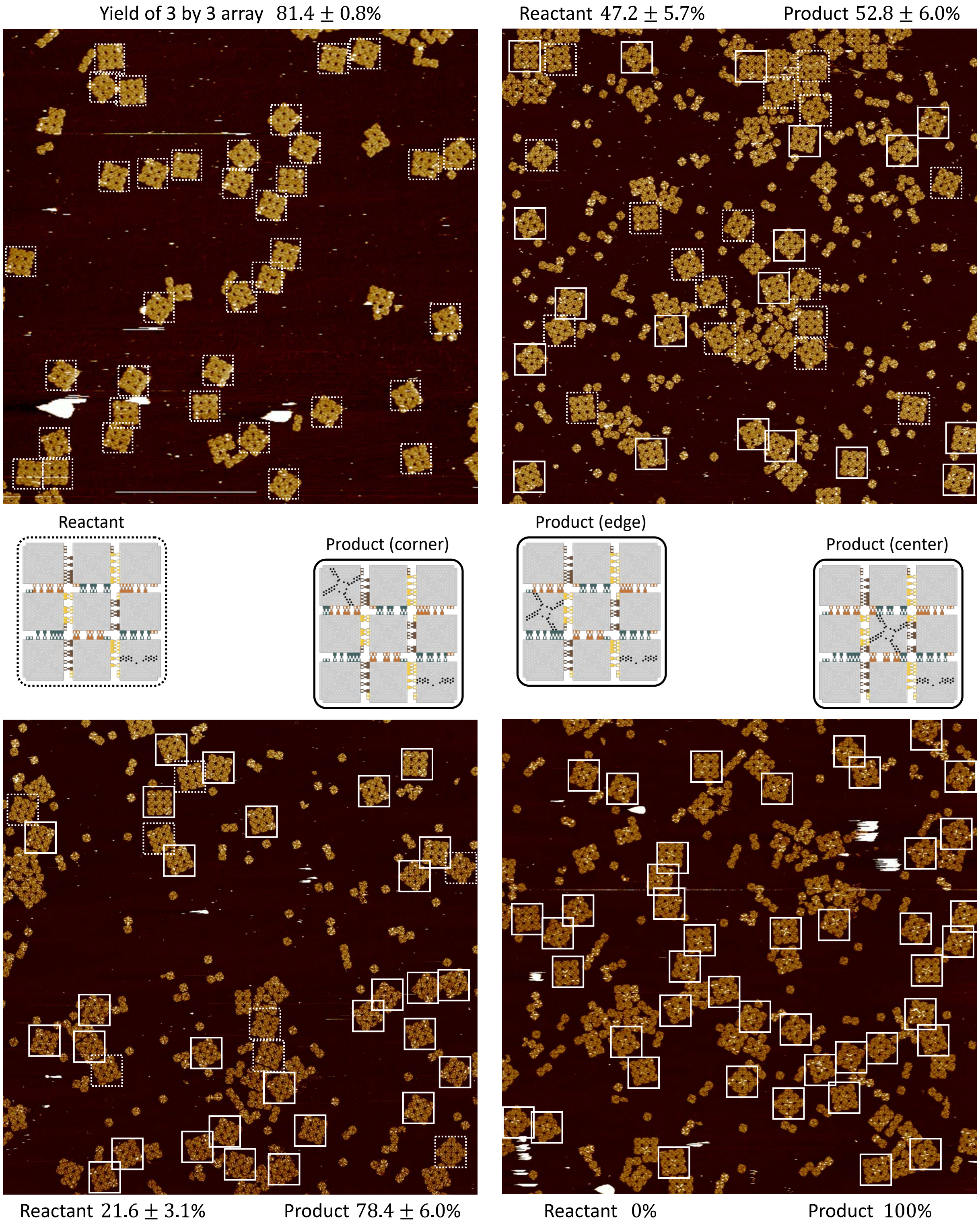

Supplementary Figure 6 | Yield estimation of three types of tile displacement reactions in 3 by 3 arrays. All AFM images are 5 by 5 μm. Each 3 by 3 array is highlighted as either a reactant (dotted boxes) or a product (solid boxes). Yield of the initial 3 by 3 arrays before tile displacement was estimated as the number of tiles in complete structures over the total number of tiles (top left). Yields of corner (bottom left), edge (top right), and center (bottom right) tile displacement reactions were each estimated as $p = m/n$, where m is the total number of products and n is the total number of reactants and products counted in each image. Ambiguous and spurious structures were not counted. The standard error was calculated as $p\sqrt{1-p}/\sqrt{n}$, treating the yield as a Bernoulli probability.

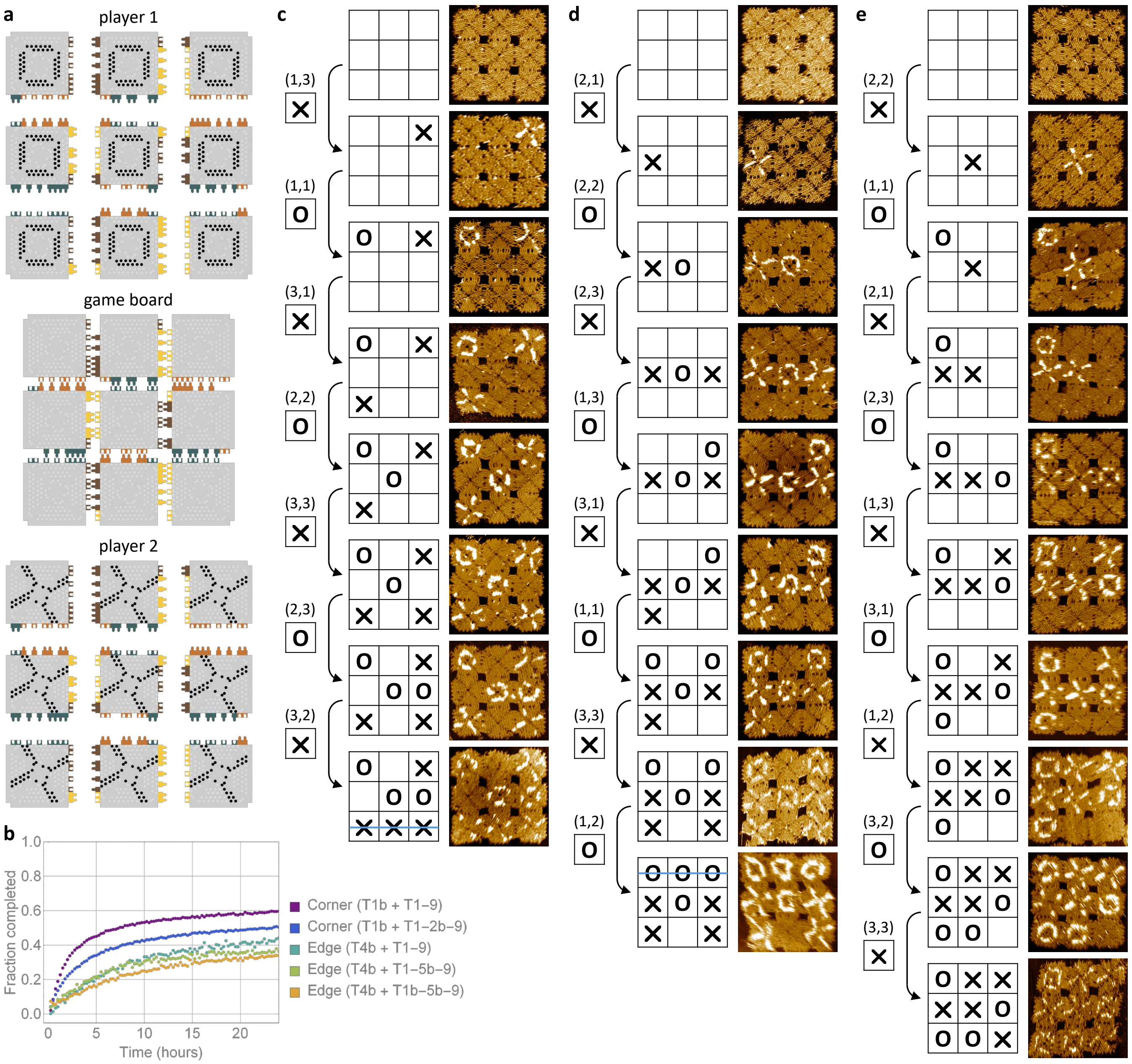

Supplementary Figure 7 | Tic-tac-toe. (**a**) Design. Two players each has 9 tiles labeled with X or O, each of which is designed to make a move by displacing one specific tile from the 3 by 3 array. All moves can be made in an arbitrary order. All toeholds are composed of 2 staples that each has a stacking bond and a 5-nt sticky end. The toeholds are of three types: near the 3' end of a tile edge and extended from the 5' end of the staples, near the 5' end of a tile edge and extended from the 5' or 3' end of the staples. All branch migration domains are composed of 6 staples that each has a stacking bond and a 2-nt sticky end, always extended from the 5' end of the staples. The branch migration domains are of three types with three unique edge codes: ___2_2_2222, 22_22_2_2__, and __22_22__22 ('_' indicates a scaffold loop and '2' indicates a 2-nt staple). Because M13 scaffold naturally has different sequences near the four edges of the square tile, three types of toeholds and three edge codes readily provide 12 unique toeholds and 12 unique branch migration domains. (**b**) Fluorescence kinetics experiments of corner and edge tile displacement reactions with different starting conditions. The tile names correspond to those shown in Fig. 4a. (**c**) Game 1. After 7 moves, X won (same as shown in Fig. 4c). (**d**) Game 2. After 8 moves, O won. (**e**) Game 3. After 9 moves, the two players tied. (x, y) indicates the position of the tile to be displaced is in row x and column y. Experiments were performed at 25 °C and AFM images were collected after 24 hours of each move. All AFM images are 285 by 285 nm.

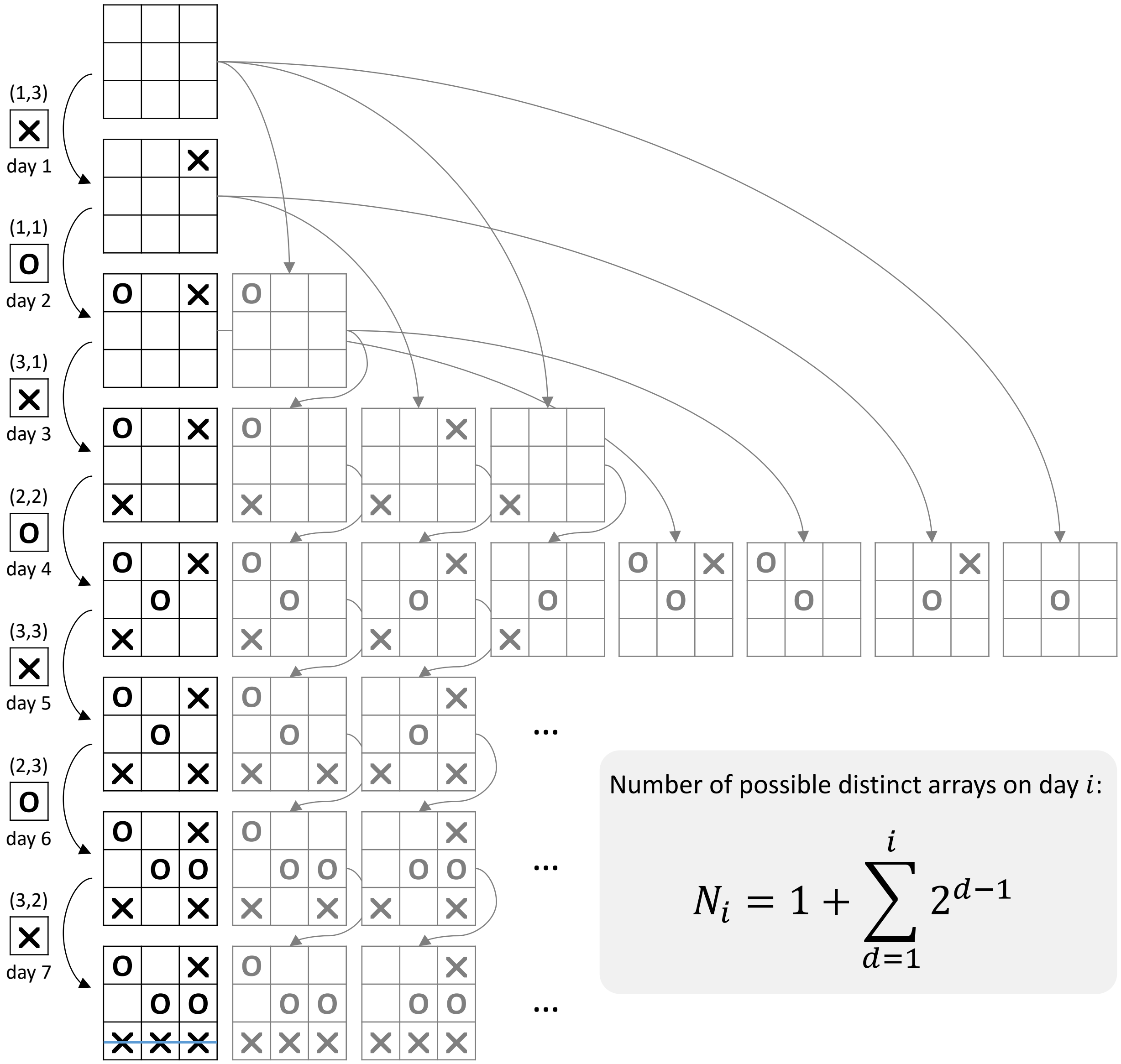

Supplementary Figure 8 | Possible distinct arrays in Game 1. On each day, when a new tile is added, it could either react with a correctly reconfigured array that has incorporated all target moves (black arrows) or react with any other array that only incorporated some of the target moves (gray arrows). Because of this, the total number of possible distinct arrays increases quickly with the number of moves played.

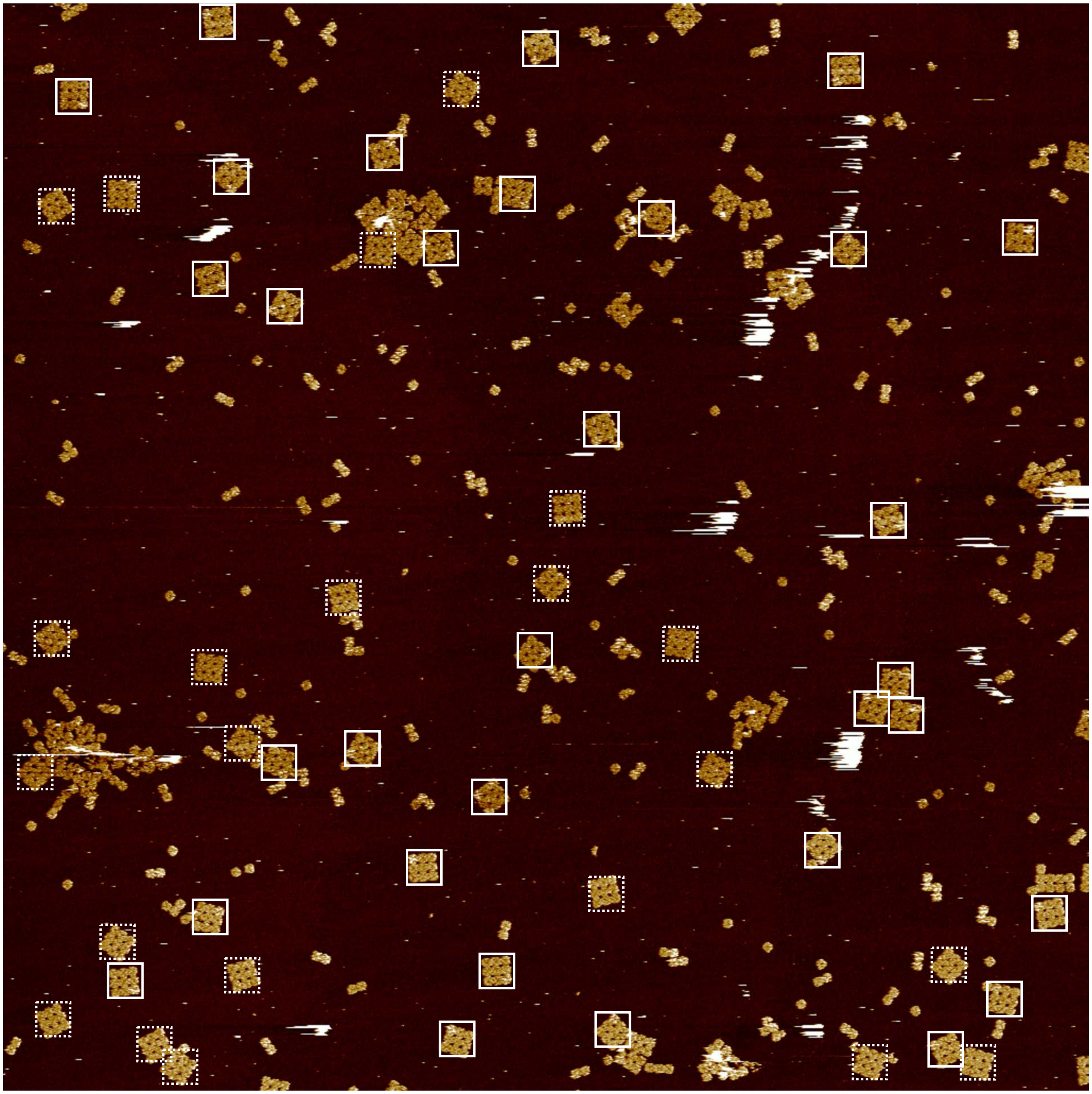

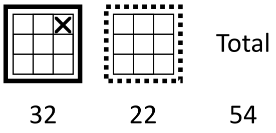

Supplementary Figure 9 | Analysis of distinct arrays in day 1 of Game 1. The AFM image is 10 by 10 μm. Each 3 by 3 array is highlighted as either a product (solid boxes) or a reactant (dotted boxes). Yield of the tile displacement reaction was estimated as $p = m/n = 59.3\%$, where $m = 32$ is the total number of products and $n = 54$ is the total number of products and reactants. Ambiguous and spurious structures were not counted. The standard error was calculated as $p\sqrt{1-p}/\sqrt{n} = 5.1\%$, treating the yield as a Bernoulli probability.

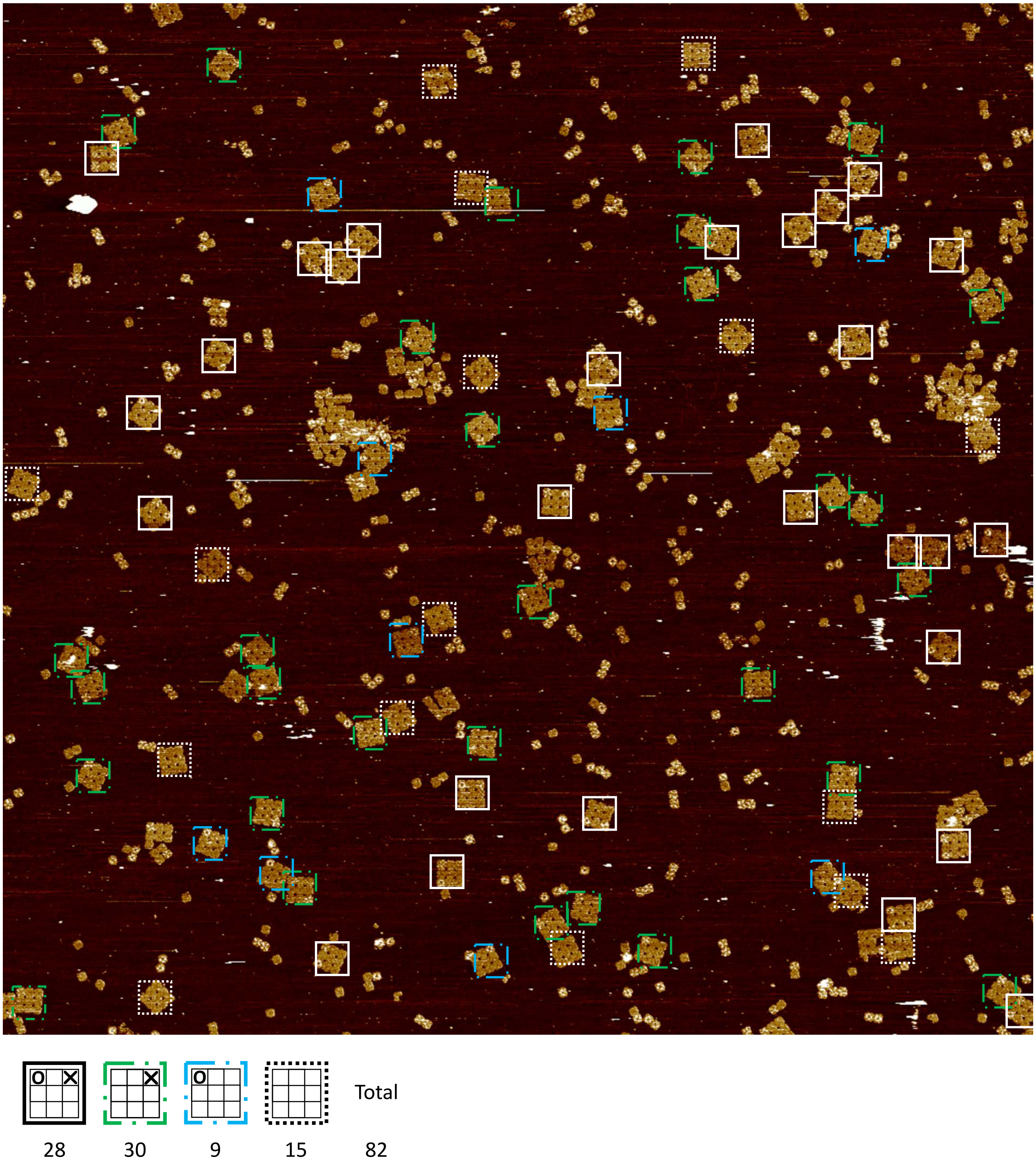

Supplementary Figure 10 | Analysis of distinct arrays in day 2 of Game 1. The AFM image is 10 by 10 μm. Each 3 by 3 array is highlighted as either a product incorporating all target moves (solid boxes), or an incomplete product incorporating only some of the moves (dashed boxes), or a reactant (dotted boxes). Yield of the tile displacement cascade was estimated as $p = m/n = 34.1\%$, where $m = 28$ is the total number of products and $n = 82$ is the total number of products, incomplete products, and reactants. Ambiguous and spurious structures were not counted. The standard error was calculated as $p\sqrt{1-p}/\sqrt{n} = 3.1\%$, treating the yield as a Bernoulli probability.

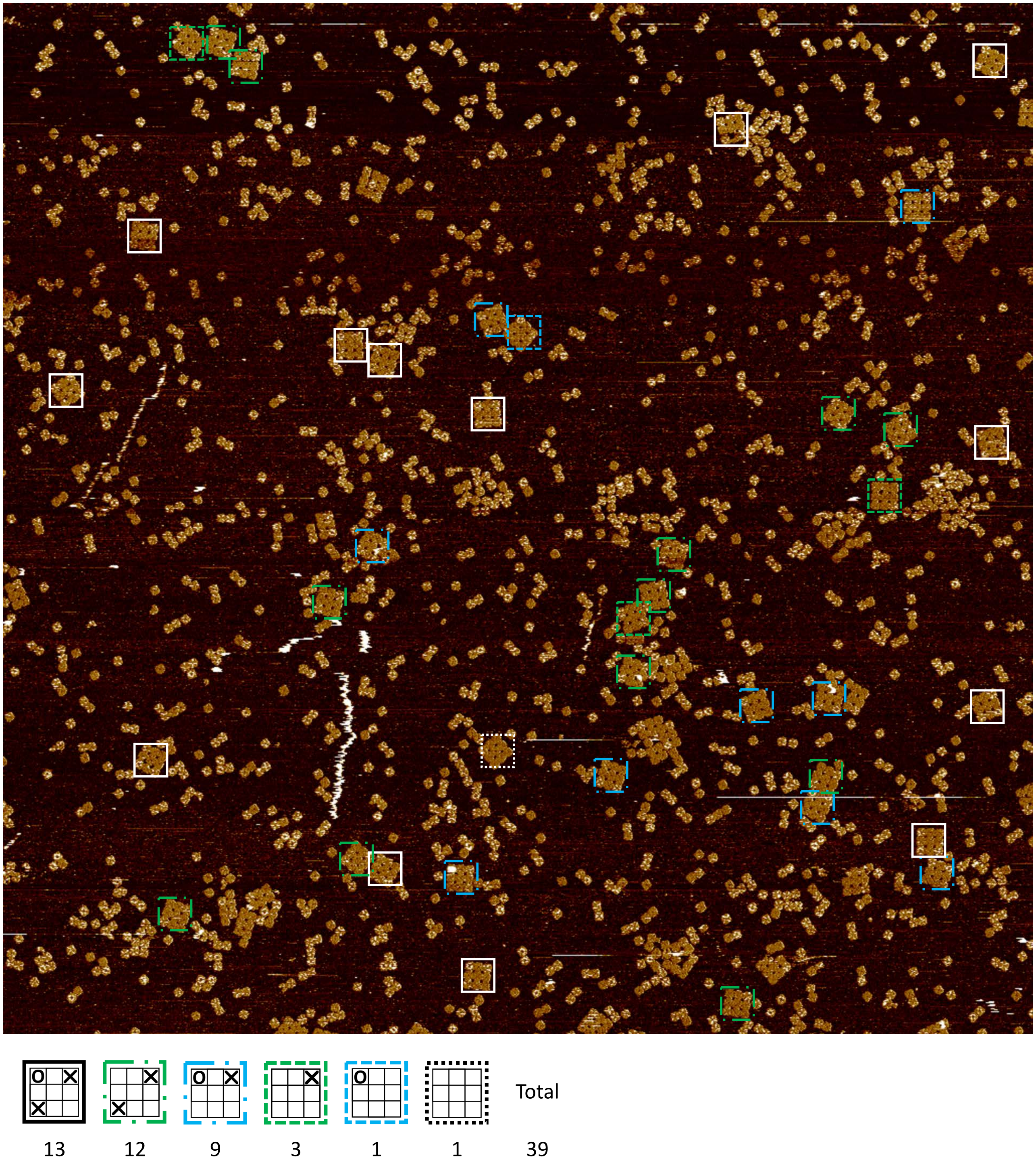

Supplementary Figure 11 | Analysis of distinct arrays in day 3 of Game 1. The AFM image is 10 by 10 μm. Each 3 by 3 array is highlighted as either a product incorporating all target moves (solid boxes), or an incomplete product incorporating only some of the moves (dashed boxes), or a reactant (dotted boxes). Yield of the tile displacement cascade was estimated as $p = m/n = 33.3\%$, where $m = 13$ is the total number of products and $n = 39$ is the total number of products, incomplete products, and reactants. Ambiguous and spurious structures were not counted. The standard error was calculated as $p\sqrt{1-p}/\sqrt{n} = 4.4\%$, treating the yield as a Bernoulli probability.

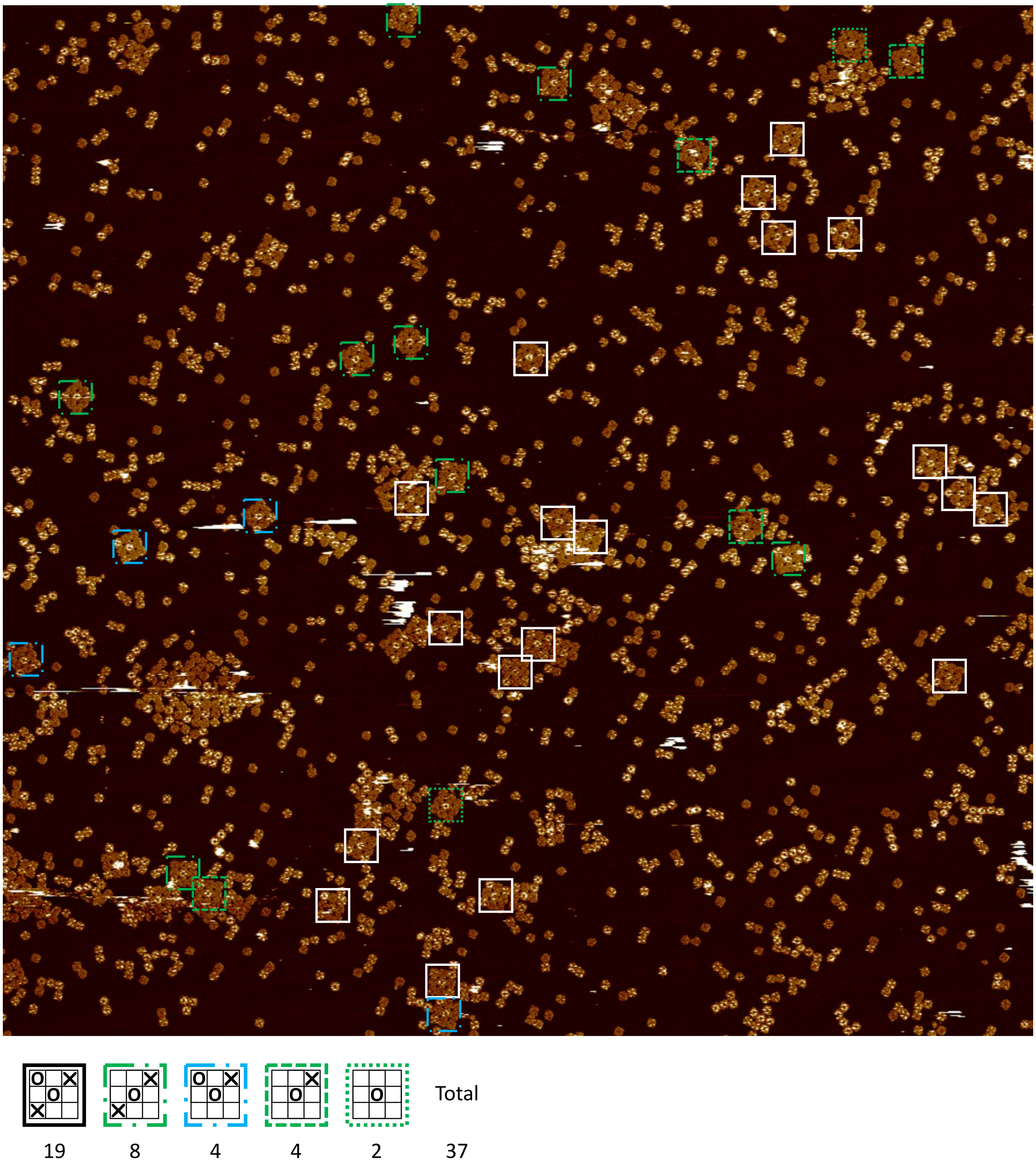

Supplementary Figure 12 | Analysis of distinct arrays in day 4 of Game 1. The AFM image is 10 by 10 μm. Each 3 by 3 array is highlighted as either a product incorporating all target moves (solid boxes) or an incomplete product incorporating only some of the moves (dashed and dotted boxes). No reactants (i.e. plain 3 by 3 arrays) were found in this image. Yield of the tile displacement cascade was estimated as $p = m/n = 51.4\%$, where $m = 19$ is the total number of products and $n = 37$ is the total number of products and incomplete products. Ambiguous and spurious structures were not counted. The standard error was calculated as $p\sqrt{1-p}/\sqrt{n} = 5.9\%$, treating the yield as a Bernoulli probability.

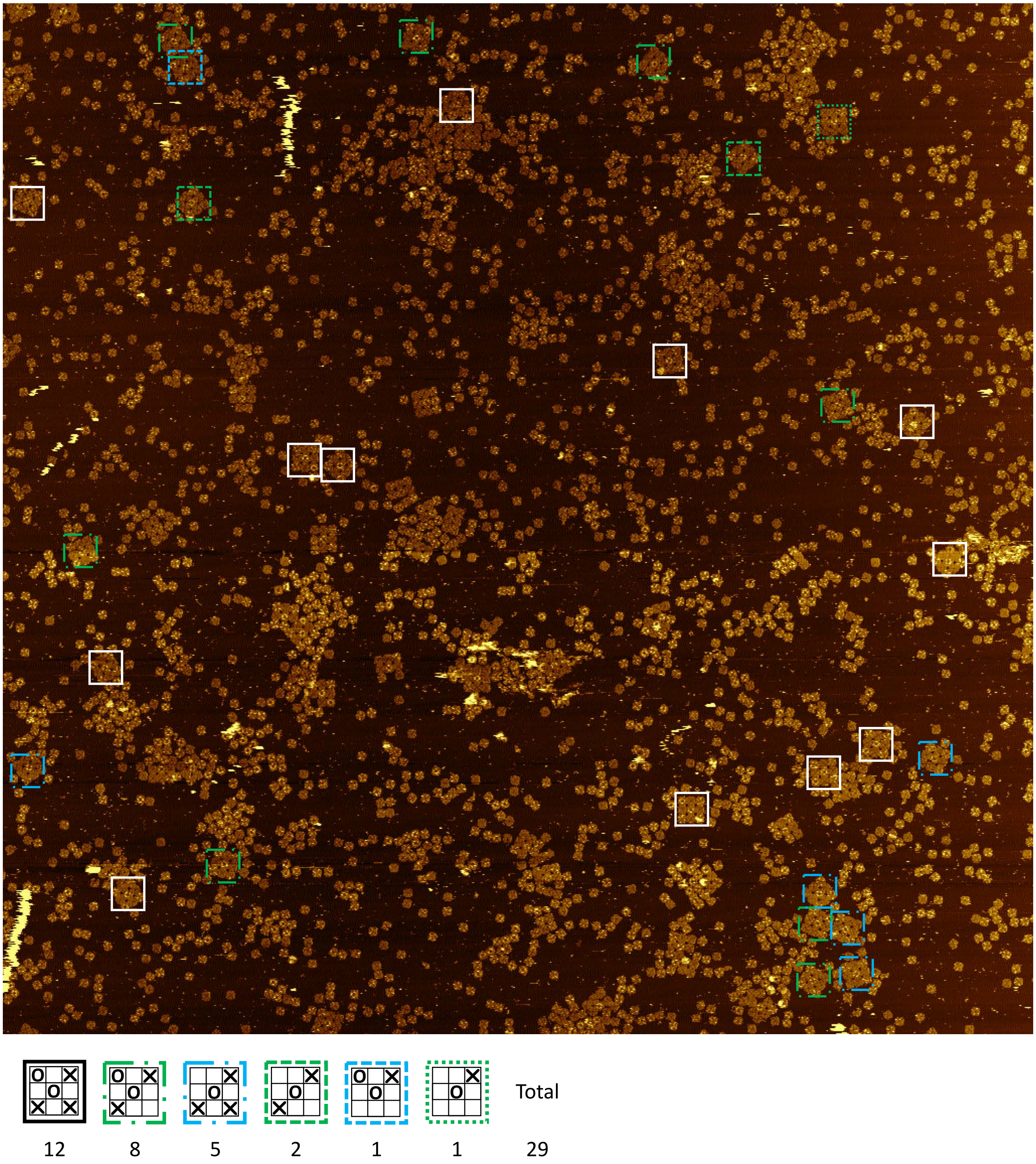

Supplementary Figure 13 | Analysis of distinct arrays in day 5 of Game 1. The AFM image is 10 by 10 μm. Each 3 by 3 array is highlighted as either a product incorporating all target moves (solid boxes) or an incomplete product incorporating only some of the moves (dashed and dotted boxes). No reactants (i.e. plain 3 by 3 arrays) were found in this image. Yield of the tile displacement cascade was estimated as $p = m/n = 41.4\%$, where $m = 12$ is the total number of products and $n = 29$ is the total number of products and incomplete products. Ambiguous and spurious structures were not counted. The standard error was calculated as $p\sqrt{1-p}/\sqrt{n} = 5.9\%$, treating the yield as a Bernoulli probability.

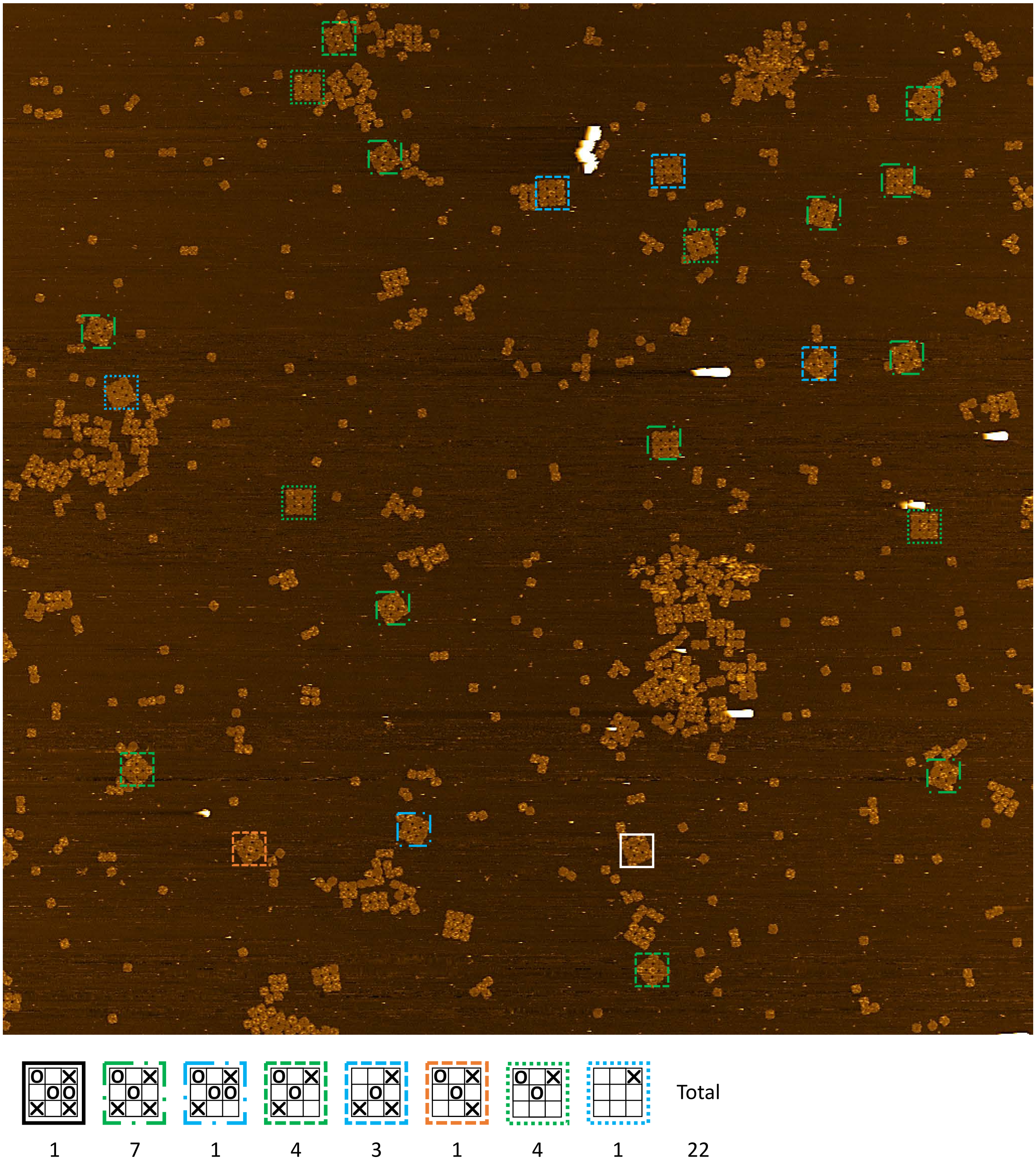

Supplementary Figure 14 | Analysis of distinct arrays in day 6 of Game 1. The AFM image is 10 by 10 μm. Each 3 by 3 array is highlighted as either a product incorporating all target moves (solid boxes) or an incomplete product incorporating only some of the moves (dashed and dotted boxes). No reactants (i.e. plain 3 by 3 arrays) were found in this image. Yield of the tile displacement cascade was estimated as $p = m/n = 4.5\%$, where $m = 1$ is the total number of products and $n = 22$ is the total number of products and incomplete products. Ambiguous and spurious structures were not counted. The standard error was calculated as $p\sqrt{1-p}/\sqrt{n} = 0.9\%$, treating the yield as a Bernoulli probability.

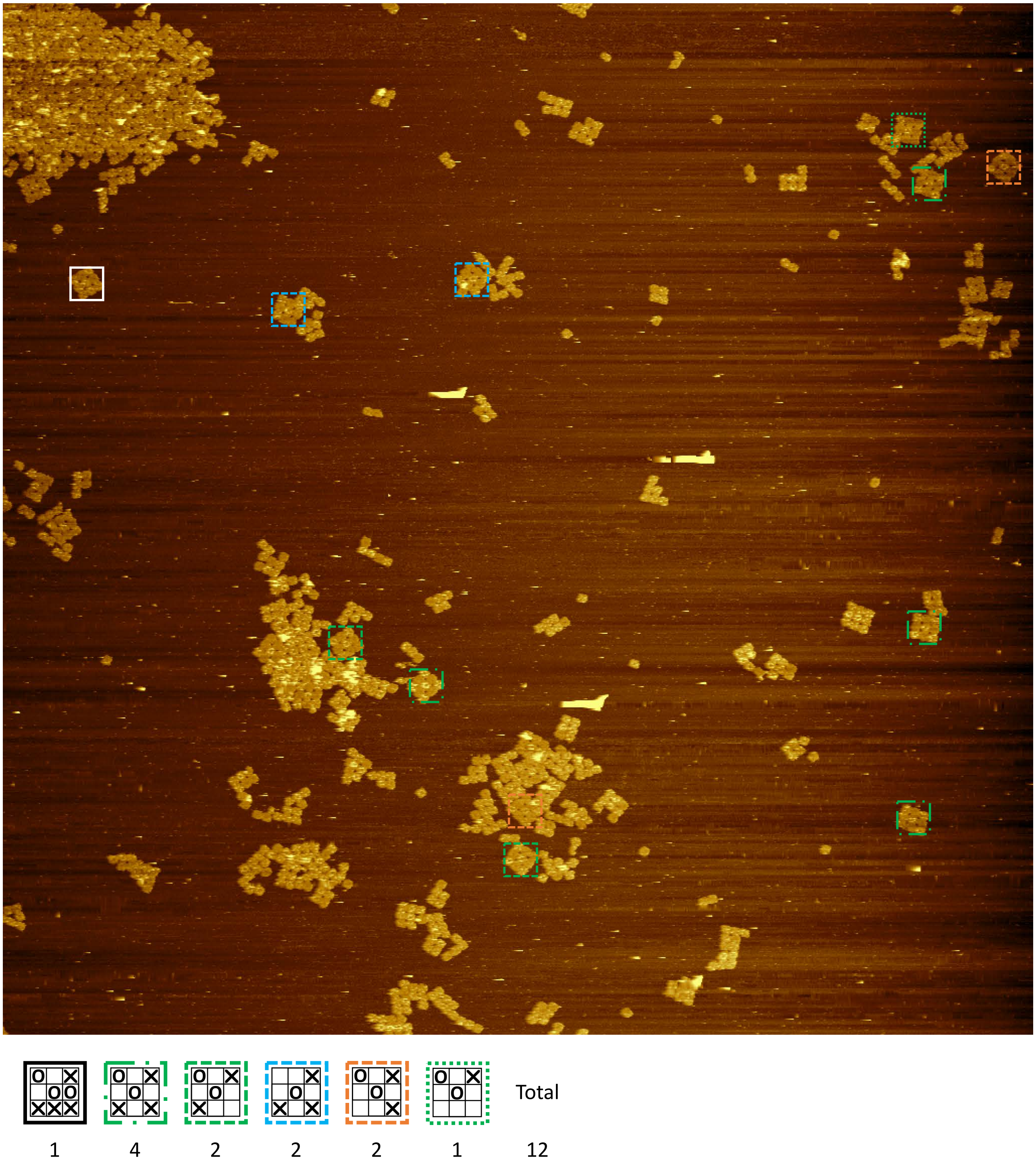

Supplementary Figure 15 | Analysis of distinct arrays in day 7 of Game 1. The AFM image is 10 by 10 μm. Each 3 by 3 array is highlighted as either a product incorporating all target moves (solid boxes) or an incomplete product incorporating only some of the moves (dashed and dotted boxes). No reactants (i.e. plain 3 by 3 arrays) were found in this image. Yield of the tile displacement cascade was estimated as $p = m/n = 8.3\%$, where $m = 1$ is the total number of products and $n = 12$ is the total number of products and incomplete products. Ambiguous and spurious structures were not counted. The standard error was calculated as $p\sqrt{1-p}/\sqrt{n} = 2.3\%$, treating the yield as a Bernoulli probability.

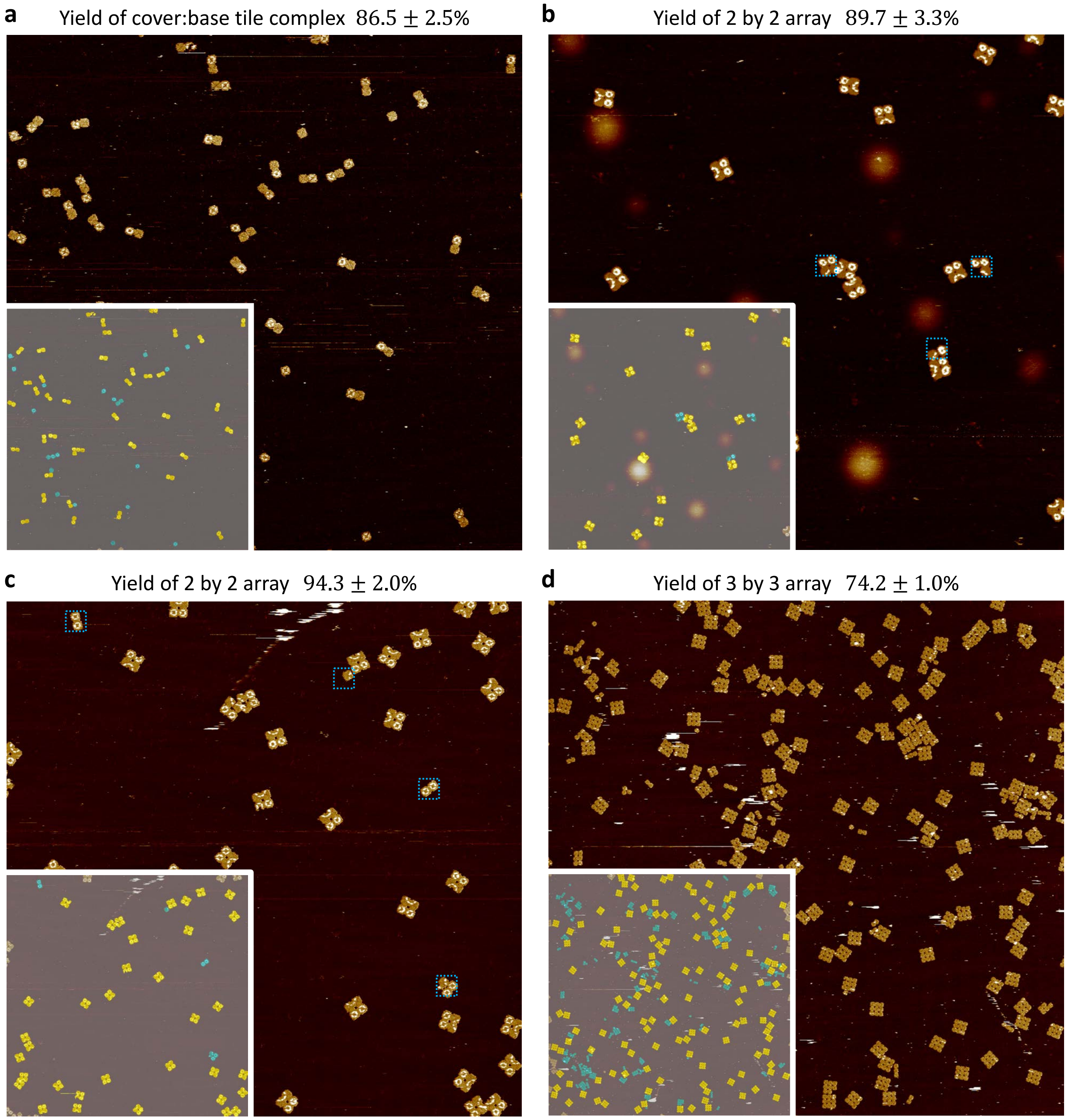

Supplementary Figure 16 | Yield estimation of tile complexes and arrays before tile displacement. (**a**) Cover:base tile complexes. Cover tile was in 20% excess. Therefore, the yield of the complexes was adjusted by ×1.1. (**b**) 2 by 2 arrays that are the reactants in sequential reconfiguration. (**c**) 2 by 2 arrays that are the reactants in cooperative reconfiguration. (**d**) 3 by 3 arrays that are the reactants in the tic-tac-toe game. The bottom right AFM image is 10 by 10 μm and the other three AFM images are 5 by 5 μm. All yields were estimated as the number of pixels in target structures over the total number of pixels in all structures, using a Yield Calculator.[3] The standard error was calculated as $p\sqrt{1-p}/\sqrt{n}$, where p is the estimated yield and n is the total number of tiles, treating the yield as a Bernoulli probability. The structures highlighted in **b** and **c** helped identifying incomplete reactants in Supplementary Figure 5.

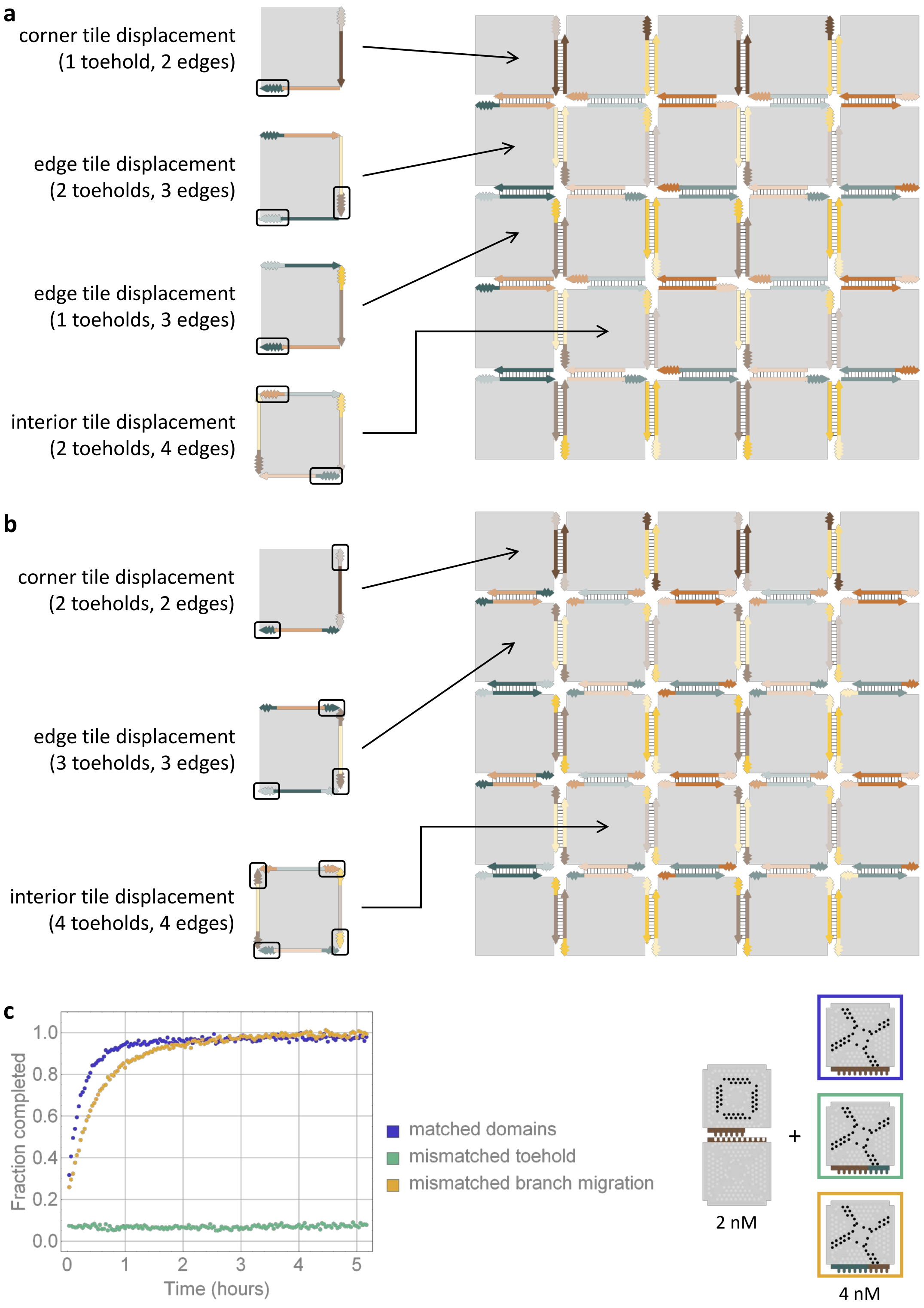

Supplementary Figure 17 | Considerations for further generalization. Design of tile displacement in a 5 by 5 array with one toehold (**a**) and two toeholds (**b**) along each edge. (**c**) Fluorescence kinetics experiments for mismatched toehold and branch migration domains. Note that these branch migration domains are continuous. For coded edges shown in Fig. 4b, even with up to 80% similarity in the sticky end sequences of the toehold staples, no incorrect tile integration was observed in AFM images (Supplementary Figure 6), indicating sufficient specificity in branch migration domains.

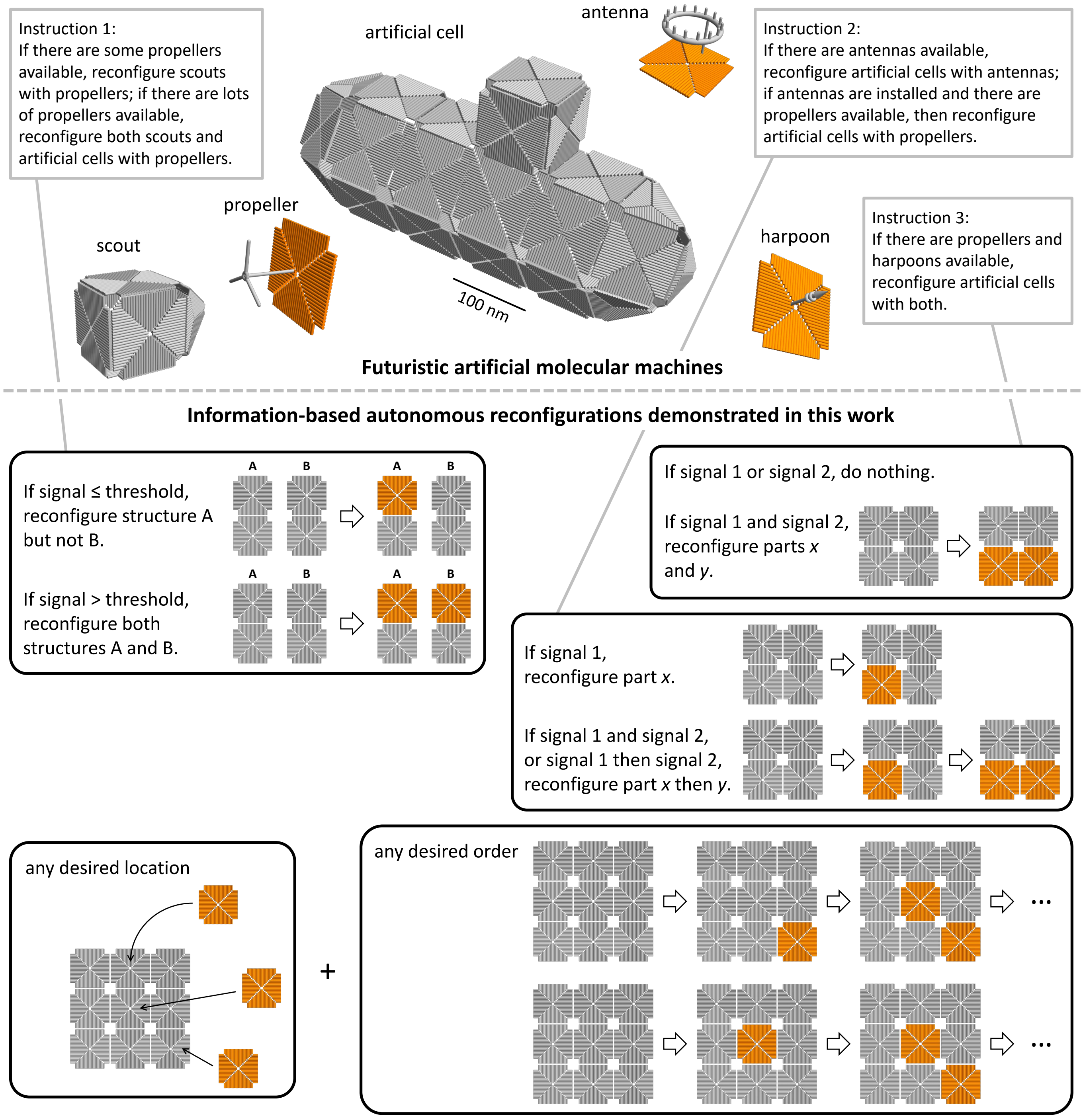

Supplementary Figure 18 | A future of adaptive artificial molecular machines. The artificial cell and scout indicate different species of molecular machines operating in the same environment. The antenna, propeller and harpoon indicate resources available at certain times or instructions sent from one molecular machine to another. Tile displacement allows pre-fabricated complex molecular components with specific spatial arrangements to be integrated into molecular machines at the right time during autonomous operations. Note that both square tiles and triangular tiles[1] shown in the artificial cell have been implemented with DNA origami tiles. The angle shown between tiles is not compatible with blunt-end stacking interactions but the tiles can bend along the seams between the isosceles triangles composing the squares and equilateral triangles to allow for desired interactions between adjacent tiles, for example as demonstrated in a 20-triangular-tile 3D structure interpreted as a rhombic triacontahedron.[1]

Supplementary Notes

Supplementary Note 1 | Modeling and simulations

Tile displacement reactions with varying toeholds shown in Fig. 1d were modeled as follows. Assume that an invader (Inv_{xy}) binds to a cover:base tile complex ($\mathrm{CT{:}BT}_x$) at rate k_{on}. The toehold either dissociates at rate k_{off}, converting the invader back to a free tile, or initiates a branch migration at rate k_{d}, resulting in release of the cover tile (CT) and an invader:base tile complex ($\mathrm{Inv}_{xy}{:}\mathrm{BT}_x$). $x = 1$ or 2 indicates the number of nucleotides in the sticky end of each edge staple. $y = 0$ through 4 indicates the number of edge staples in the toehold domain.

$$\mathrm{CT{:}BT}_x + \mathrm{Inv}_{xy} \underset{k_{\mathrm{off}}}{\overset{k_{\mathrm{on}}}{\rightleftharpoons}} \mathrm{CT{:}BT}_x{:}\mathrm{Inv}_{xy} \xrightarrow{k_{\mathrm{d}}} \mathrm{CT} + \mathrm{Inv}_{xy}{:}\mathrm{BT}_x \tag{1}$$

With this model, we simulated the reactions with mass-action kinetics by solving the differential equations involved in all reaction steps using a CRN Simulator Package.[4] Comparing the simulations with experimental data (Fig. 1f), including additional experiments with varying concentrations of the invader (Supplementary Figure 2b), we found a set of parameters that explained the data reasonably well.

$$\begin{aligned} k_{\mathrm{on}} &= \begin{cases} 2.5\times10^{4}\ \mathrm{M^{-1}s^{-1}} & \text{if } x=1 \\ 4.5\times10^{5}\ \mathrm{M^{-1}s^{-1}} & \text{if } x=2 \end{cases} \\ k_{\mathrm{off}} &= \begin{cases} 10^{1-1.1y}\ \mathrm{s^{-1}} & \text{if } x=1 \\ 10^{3-2y}\ \mathrm{s^{-1}} & \text{if } x=2 \end{cases} \\ k_{\mathrm{d}} &= 0.025\ \mathrm{s^{-1}} \end{aligned} \tag{2}$$

These parameters led to three interpretations: First, the binding rate for 1-nt toehold staples is slower than that for 2-nt ones, both of which are slower than the binding rate of a strand displacement reaction (same as the rate of DNA hybridization[5]). This is likely because the entropic cost for parts of the invader and cover tiles bending out of the way to allow for toehold binding (Supplementary Figure 2a) makes it difficult for the first pair of staples to successfully make a contact. A longer sticky end makes it geometrically easier for the success, or energetically allows for the first contact to hang on long enough to zip up the rest of the staples rather than falling off immediately. Second, similar to strand displacement, the dissociation rate decreases exponentially with increasing number of nucleotides in the toehold, which depends on both the number of toehold staples and the length of each sticky end. However, with 0 toehold staples, the same invader dissociates faster if the base tile has 1-nt toehold staples, presumably because the spurious interactions between the branch migration domain in the invader and the toehold in the base tile is more significant for 2-nt toehold staples. Lastly, the displacement rate is approximately 40 times slower than a strand displacement reaction. This is not surprising because each branch migration step requires the dissociation of a stacking bond and a 2-nt sticky end in a staple rather than just a single nucleotide, and it requires both the invader and the cover tiles to adjust their degree of bending (Supplementary Figure 2a). Overall, the tile displacement reaction was completed along 65 nm of 22 helices on the edge of a DNA origami structure, a much larger scale compared to a typical strand displacement reaction along 7 nm of 21 base pairs.

Competitive tile displacement reactions shown in Fig. 2a were modeled as follows.

$$\begin{aligned} \mathrm{CT{:}BT1} + \mathrm{Inv} &\underset{k^{1}_{\mathrm{off}}}{\overset{k^{1}_{\mathrm{on}}}{\rightleftharpoons}} \mathrm{CT{:}BT1{:}Inv} \xrightarrow{k_{\mathrm{d}}} \mathrm{CT} + \mathrm{Inv{:}BT1} \\ \mathrm{CT{:}BT2} + \mathrm{Inv} &\underset{k^{2}_{\mathrm{off}}}{\overset{k^{2}_{\mathrm{on}}}{\rightleftharpoons}} \mathrm{CT{:}BT2{:}Inv} \xrightarrow{k_{\mathrm{d}}} \mathrm{CT} + \mathrm{Inv{:}BT2} \end{aligned} \tag{3}$$

The same rate constants for the 1-nt 4-toehold-staple ($x = 1$ and $y = 4$) and 2-nt 4-toehold-staple ($x = 2$ and $y = 4$) reactions shown above were used here.

$$\begin{aligned} k^{1}_{\mathrm{on}} &= 2.5\times10^{4}\ \mathrm{M^{-1}s^{-1}} \\ k^{2}_{\mathrm{on}} &= 4.5\times10^{5}\ \mathrm{M^{-1}s^{-1}} \\ k^{1}_{\mathrm{off}} &= 10^{1-1.1\times4} = 4.0\times10^{-4}\ \mathrm{s^{-1}} \\ k^{2}_{\mathrm{off}} &= 10^{3-2\times4} = 10^{-5}\ \mathrm{s^{-1}} \\ k_{\mathrm{d}} &= 0.025\ \mathrm{s^{-1}} \end{aligned} \tag{4}$$

Sequential tile displacement reactions shown in Fig. 3a were modeled as follows.

$$\begin{aligned} &\mathrm{T1a2a34} + \mathrm{T2b} \underset{k_{\mathrm{off}}^{1}}{\overset{k_{\mathrm{on}}}{\rightleftharpoons}} \mathrm{M1} \underset{k_{\mathrm{d}}}{\overset{k_{\mathrm{d}}}{\rightleftharpoons}} \mathrm{M2} \underset{k_{\mathrm{on}}}{\overset{k_{\mathrm{off}}^{2}}{\rightleftharpoons}} \mathrm{T1a2b34} + \mathrm{T2a} \\ &\mathrm{T1a2b34} + \mathrm{T1b2c} \underset{k_{\mathrm{off}}^{2}}{\overset{k_{\mathrm{on}}}{\rightleftharpoons}} \mathrm{M3} \xrightarrow{k'_{\mathrm{d}}} \mathrm{T1b2c34} + \mathrm{T1a2b} \end{aligned} \tag{5}$$

We used the same binding ($x = 2$) and displacement rate constants obtained from the above experiments and revised the model to include a branch migration step across the corners of two adjacent tiles. We expect the rate of this step (k'_{d}) to be slower than that within the same tile edge (k_{d}), because the square DNA origami tile has an empty area near the corners and thus there should be an entropic cost for closing the gap, similar to the cost for closing a loop in DNA strands. We also used the same dissociation rate constant ($x = 2$ and $y = 4$) for the toehold between T1 and T2 (k_{off}^{1}), but allowed that for the toehold between T2 and T3 to be different (k_{off}^{2}). We found the following values of k_{off}^{2} and k'_{d} that agreed with the data reasonably well.

$$\begin{aligned} k_{\mathrm{on}} &= 4.5 \times 10^{5}\ \mathrm{M}^{-1}\mathrm{s}^{-1} \\ k_{\mathrm{off}}^{1} &= 10^{-5}\ \mathrm{s}^{-1} \\ k_{\mathrm{off}}^{2} &= 10^{-4}\ \mathrm{s}^{-1} \\ k_{\mathrm{d}} &= 0.025\ \mathrm{s}^{-1} \\ k'_{\mathrm{d}} &= 1.3 \times 10^{-4}\ \mathrm{s}^{-1} \end{aligned} \tag{6}$$

Cooperative tile displacement reactions shown in Fig. 3c were modeled as follows. M2 and M4 are two possible intermediate states with one or the other invader branch migrated to the center of the array, both of which can reach a common intermediate state M5 where a second invader is now bound to the array. With another branch migration step, two tiles that are originally part of the array will be no longer attached and the two invaders will be fully bound as part of the reconfigured 2 by 2 array. Once reaching the final state, the process should become irreversible.

$$\begin{aligned} &\mathrm{T1a2a34} + \mathrm{T1b} \underset{k_{\mathrm{off}}}{\overset{k_{\mathrm{on}}}{\rightleftharpoons}} \mathrm{M1} \underset{k_{\mathrm{d}}}{\overset{k_{\mathrm{d}}}{\rightleftharpoons}} \mathrm{M2} \\ &\mathrm{T1a2a34} + \mathrm{T2b} \underset{k_{\mathrm{off}}}{\overset{k_{\mathrm{on}}}{\rightleftharpoons}} \mathrm{M3} \underset{k_{\mathrm{d}}}{\overset{k_{\mathrm{d}}}{\rightleftharpoons}} \mathrm{M4} \\ &\mathrm{M2} + \mathrm{T2b} \underset{k_{\mathrm{off}}}{\overset{k_{\mathrm{on}}}{\rightleftharpoons}} \mathrm{M5} \\ &\mathrm{M4} + \mathrm{T1b} \underset{k_{\mathrm{off}}}{\overset{k_{\mathrm{on}}}{\rightleftharpoons}} \mathrm{M5} \\ &\mathrm{M5} \xrightarrow{k_{\mathrm{d}}} \mathrm{T1b2b34} + \mathrm{T1a2a} \end{aligned} \tag{7}$$

We used the same binding and displacement rate constants as above, but tuned the dissociation rate to better explain the kinetics observed from the fluorescence experiments.

$$\begin{aligned} k_{\mathrm{on}} &= 4.5 \times 10^{5}\ \mathrm{M}^{-1}\mathrm{s}^{-1} \\ k_{\mathrm{off}} &= 10^{-2}\ \mathrm{s}^{-1} \\ k_{\mathrm{d}} &= 0.025\ \mathrm{s}^{-1} \end{aligned} \tag{8}$$

To explore the impact of invader dimerization, two additional reactions were introduced in a separate simulation (shown as the lighter yellow trajectory in Fig. 3d):

$$\begin{aligned} &\mathrm{T1b} + \mathrm{T1b} \underset{k_{\mathrm{off}}^{\mathrm{d}}}{\overset{k_{\mathrm{on}}}{\rightleftharpoons}} \mathrm{D1b} \\ &\mathrm{T2b} + \mathrm{T2b} \underset{k_{\mathrm{off}}^{\mathrm{d}}}{\overset{k_{\mathrm{on}}}{\rightleftharpoons}} \mathrm{D2b} \end{aligned} \tag{9}$$

With dimer dissociation rate constant $k^{\mathrm{d}}_{\mathrm{off}} = 3.2 \times 10^{-4}\ \mathrm{s}^{-1}$, which is about 30 times slower than toehold dissociation, the simulation showed a decreased completion level of reconfiguration comparable to the experimental data. The reality is likely to be more complex: the invader dimer could still trigger tile displacement but at a much slower rate, similar to how four-way branch migration is much slower than three-way branch migration in strand displacement systems.

Corner, edge, and center tile displacement reactions shown in Fig. 4a were modeled as follows. For center tile displacement (last reaction), we only included the reaction steps along one edge and assumed that the same reaction steps along other edges will take place simultaneously.

$$\begin{aligned}
&\text{T1-9} + \text{T1b} \underset{k_{\mathrm{off}}}{\overset{k_{\mathrm{on}}}{\rightleftharpoons}} \text{M1} \underset{k'_{\mathrm{d}}}{\overset{k_{\mathrm{d}}}{\rightleftharpoons}} \text{M2} \xrightarrow{k'_{\mathrm{d}}} \text{T1b-9} + \text{T1} \\
&\text{T1-9} + \text{T4b} \underset{k_{\mathrm{off}}}{\overset{k_{\mathrm{on}}}{\rightleftharpoons}} \text{M1} \underset{k'_{\mathrm{d}}}{\overset{k_{\mathrm{d}}}{\rightleftharpoons}} \text{M2} \underset{k'_{\mathrm{d}}}{\overset{k'_{\mathrm{d}}}{\rightleftharpoons}} \text{M3} \xrightarrow{k'_{\mathrm{d}}} \text{T1-4b-9} + \text{T4} \\
&\text{T1-9} + \text{T5b} \underset{k_{\mathrm{off}}}{\overset{k_{\mathrm{on}}}{\rightleftharpoons}} \text{M1} \xrightarrow{k'_{\mathrm{d}}} \text{T1-5b-9} + \text{T5}
\end{aligned} \tag{10}$$

Compared to edges with continuous staples, the displacement rate constant k_{d} was estimated to be roughly 25 times slower for coded edges. All other rate constants remained the same.

$$\begin{aligned}
k_{\mathrm{on}} &= 4.5 \times 10^{5}\ \mathrm{M}^{-1}\mathrm{s}^{-1} \\
k_{\mathrm{off}} &= 10^{-5}\ \mathrm{s}^{-1} \\
k_{\mathrm{d}} &= 10^{-3}\ \mathrm{s}^{-1} \\
k'_{\mathrm{d}} &= 1.3 \times 10^{-4}\ \mathrm{s}^{-1}
\end{aligned} \tag{11}$$

When a corner tile displacement reaction takes place after the adjacent edge tile (whose invader binds to this corner tile) has already been displaced, the second branch migration step will have to go though an additional toehold domain and thus become a bit slower (Supplementary Figure 7b). Similarly, when an edge tile displacement reaction takes place after the center tile has already been displaced (or after both the center tile and the relevant corner tile have been displaced), the second branch migration step (or both the second and third branch migration steps) will become a bit slower.

Supplementary Note 2 | An earlier design of sequential reconfiguration

At first, to test the possibility of using coded edges as branch migration domains, which is necessary for the self-assembly of DNA origami arrays with increasing size,[6,7] we left out a few edge staples in all four pairs of edge interactions in the 2 by 2 array for demonstrating sequential tile displacement (Supplementary Figure 4a, e). However, unexpectedly, the one-step reaction starting from a 2 by 2 array that was already the product of the first step was almost as slow as the two steps in cascade (Supplementary Figure 4b). There was also a lack of delay in the two-step reaction as predicted by simulations.

What could have happened that was not as designed when the molecules were mixed together? We came up with a hypothesis of an alternative reconfiguration pathway (Supplementary Figure 4d): at the intermediate state M2, instead of proceeding with displacement across a tile corner, dissociation could occur and remove a tile from the 2 by 2 array, turning it into a three-tile complex. When the second invader arrives, it could then bind by an entire tile edge rather than a toehold and displace one tile from the three-tile complex, forming a 2 by 2 array that is the same as the designed product. In this pathway, the first tile being removed was the one with a quencher, and thus fluorescence signal would increase as soon as the first half of the pathway has been completed.

As this hypothesis would explain what we observed from the fluorescence kinetics experiments, we looked for evidence in an AFM experiment where the 2 by 2 arrays were mixed together with only the first invader. Two possible products of the alternative pathway, the three-tile complex and the dissociated two-tile complex, were both found in the images (Supplementary Figure 4e). Products from the designed (and alternative) pathway was estimated to be $71.0 \pm 6.9\%$ (and $22.6 \pm 3.6\%$) or $56.4 \pm 6.0\%$ (and $38.5 \pm 4.8\%$), comparing to the three-tile or two-tile complex, respectively. Comparing to the three-tile complex is likely to be an overestimate of the designed pathway because the complex could further dissociate into smaller structures. Comparing to the two-tile complex is likely to be an underestimate of the designed pathway because the complex looks similar to a spurious product of the excess invader tile binding to the displaced tile of the designed pathway. With these estimations, we concluded that the alternative pathway indeed played a significant role in the sequential reconfiguration.

Therefore, we redesigned the system to have continuous edge staples within the branch migration domains, which strengthened the binding between adjacent tiles that should be held together at any intermediate steps of the desired reaction pathway (Fig. 3a). As tile displacement reactions take place at room temperature, we need to consider possible aggregations of the invaders. In revising branch migration domains from coded edges to continuous edges, we also revised the two-tile invader to have just one rather than two branch migration domains to reduce aggregation.

Supplementary Discussion

Three main difficulties have to be considered for scaling up tile displacement systems: First, in addition to the impurity of the staple strands, the yield of the DNA origami arrays before tile displacement (Supplementary Figure 16) also limits the success of reconfiguration. While previously developed criteria for assembling DNA origami arrays[6,7] remain useful, new criteria are required for creating arrays that are compatible with tile displacement. For example, we exclusively used staple extensions rather than truncations in toeholds on the invaders, for the purpose of promoting faster toehold binding with more molecular fluctuation of the free sticky ends as well as reducing spurious interactions among diverse invaders. For where the toeholds need to be in an array, the tiles may not satisfy the previously established giving and receiving rule.[7] Second, as reconfiguration takes place at room temperature, aggregation becomes more of a concern and limits the complexity of the invaders. For example, a two-tile invader with an uncovered toehold and branch migration domain across two tile edges (for example, the second invader in sequential tile displacement shown in Supplementary Figure 4a) could aggregate if the spurious interactions including a non-specific stacking bond in each staple is significant. Finally, because the displacement step, especially across a tile corner, is much slower than that in strand displacement reactions, it is important to evaluate if any intermediate states could lead to spurious reaction steps that would affect the desired reconfiguration.

Unlike in strand displacement where a reaction is initiated by matching domains in 1D (for example, a desired toehold next to a desired branch migration domain), specific 2D geometries are required to initiate a tile displacement reaction (for example, two adjacent branch migration domains in a 90 degree angle). This richness in geometry could be exploited to allow information-based reconfiguration to be carried out more efficiently and more robustly than otherwise possible.

Compared to other strategies for structural reconfiguration, for example incorporating flexible components[8,9] or manipulating individual strands in a DNA nanostructure,[10–14] tile displacement has distinct advantages for demonstrating reconfiguration in systems of multiple structures and for swapping in and out complex functional components without requiring disassembly and reassembly. For example, the arbitrary patterning capability of DNA origami has enabled the fabrication of functional components in devices with nanometer precision, including routing nanotubes[15] and polymers[16] for creating nanoscale electronic circuits, organizing metal nanoparticles[17] and nanorods[18] for creating nanoscale optical circuits,[19] and arranging quantum dots[20] for creating quantum-dot cellular automata.[21,22] In principle, tile displacement now opens up the possibility for complex components in these devices to be reconfigured and thus adapt their functions in response to given instructions. In these reconfiguration tasks, pre-fabricated components could be simply swapped in and out without changes in the spatial arrangement of the molecules tethered to individual tile surfaces, making the process highly efficient.

Furthermore, there is a fundamental difference between tile displacement and the possibility of reconfiguring multi-origami structures by using a set of DNA strands to disconnect some components and then reconnect others:[23] the former is intrinsically suited for information-based autonomous reconfiguration, while the latter would require DNA strands to be added to the test tube for each reconfiguration step. This difference is similar to that between nonautonomous[24,25] and autonomous[26,27] DNA walkers. Importantly, the ability to perform cascades of structural reconfiguration without external fuels (as shown in sequential reconfiguration) will allow artificial molecular machines to carry out sophisticated tasks by themselves. In principle, strand displacement circuits are also capable of directing self-assembly and reconfiguration without external fuels,[28] but there has been no experimental demonstration beyond simple self-assembly processes in a single step.[29]

Taking advantage of the autonomous reconfiguration, it should be possible to design an “intelligent” game board that can make a move in response to a human player’s move, similar to the classic tic-tac-toe game played by a deoxyribozyme-based automaton.[30] However, without the global information of all pieces that have been played, the molecular decision could only be made based on local information of the neighboring tiles. To enhance the capabilities of tile displacement, the reaction mechanism could be combined with DNA strand displacement: a single-stranded output signal could be released upon completion of a tile displacement reaction, triggering computation carried out by a DNA circuit, similar to the self-assembly-triggered chemical reactions discussed in a theoretical model.[28] The circuit output could then be programmed to activate sticky ends[29] of a toehold domain on a tile and trigger another tile displacement reaction, allowing dynamic behaviors at different scales to be integrated together. As articulated in spatially-organized DNA circuits[31] and robots,[32] parallel behaviors could be obtained with the compartmentalization provided by DNA nanostructures. The integration of tile displacement and strand displacement could further allow these parallel behaviors to involve structural reconfiguration, for example the robots could send a signal to swap structures after the task on a particular structure has been completed.

Supplementary Tables

Supplementary Table 1 | Interior staples.

Strand name	Sequence
Reg-T1R01C6	TCATTTGCTAATAGTAGTAGCATT
Reg-T1R03C5	CAACTAAAGTACGGTGGGATGGCT
Reg-T1R03C6	TTTCATTGAGTAGATTTAGTTTCTATATTT
Reg-T1R04C5	TAGAGCTTCAGACCGGAAGCAAACCTATTATA
Reg-T1R05C6	GTCAGGAAGAGGTCATTTTTGCTCTGGAAG
Reg-T1R06C3	TTAAGAGGGTCCAATACTGCGGATAGCGAG
Reg-T1R06C5	GTCAGAAGATTGAATCCCCCTCAACCTCGTTT
Reg-T1R07C4	AAATATTCCAAAGCGGATTGCATCGAGCTTCA
Reg-T1R07C6	AACAGTTAGGTCTTTACCCTGATCCAACAG
Reg-T1R08C3	AGGCTTTTCAGGTAGAAAGATTCAATTACC
Reg-T1R08C5	ACCAGACGGAATACCACATTCAACGAGATGGT
Reg-T1R09C2	CATTATTAGCAAAAGAAGTTTTGC
Reg-T1R09C4	AGATTTAGACGATAAAAACCAAAAATCGTCAT
Reg-T1R09C6	ATACATACAACACTATCATAACATGCTTTA
Reg-T1R10C1	AGTCAGGACATAGGCTGGCTGACCTTTGAAAG
Reg-T1R10C3	TTATGCGATTGACAAGAACCGGAGGTCAAT
Reg-T1R10C5	TTAATTTCCAACGTAACAAAGCTGTCCATGTT
Reg-T1R11C2	GAGTAATCTTTTAAGAACTGGCTCCGGAACAA
Reg-T1R11C4	ACCCAAATAACTTTAATCATTGTGATCAGTTG
Reg-T1R11C6	GTGAATATAGTAAATTGGGCTTTAATGCAG
Reg-T1R12C3	CATAAGGGACACTAAAACACTCACATTAAA
Reg-T1R12C5	ACTTAGCCATTATACCAAGCGCGAGAGGACTA
Reg-T1R13C2	AAAAGAATAACCGAACTGACCAACTTCATCAA
Reg-T1R13C4	CCCCAGCGGGAACGAGGCGCAGACTATTCATT
Reg-T1R13C6	ACAACGGAAATCCGCGACCTGCCTCATTCA
Reg-T1R14C3	CGGGTAAAATTCGGTCGCTGAGGAATGACA
Reg-T1R14C5	AAGACTTTGGCCGCTTTTGCGGGATTAAACAG
Reg-T1R15C4	GAGTTAAATTCATGAGGAAGTTTCTCTTTGAC
Reg-T1R15C6	CTCAGCAGGCTACAGAGGCTTTAACAAAGT
Reg-T1R16C5	CTTGATACTGAAAATCTCCAAAAAAGCGGAGT
Reg-T1R17C4	TTTCACGTCGATAGTTGCGCCGACCTTGCAGG
Reg-T1R17C6	CAAAAGGTTCGAGGTGAATTTCTCGTCACC
Reg-T1R19C6	GTTAGTAACTTTCAACAGTTTCAAAGGCTC
Reg-T1R21C5	CCATGTACCGTAACACTGTAGCATTCCACAGATTCCAGAC
Reg-T2R01C6	ACCCTCATTCAGGGATAGCAAGCC
Reg-T2R03C5	TTAGGATTAGCGGGGTGGAACCTA
Reg-T2R03C6	GTACCAGGTATAGCCCGGAATAGAACCGCC
Reg-T2R04C5	TTATTCTGACTGGTAATAAGTTTTAACAAATA
Reg-T2R05C6	CAGTGCCCCCCCTGCCTATTTCTTTGCTCA
Reg-T2R06C3	GTCTCTGACACCCTCAGAGCCACATCAAAA
Reg-T2R06C5	AATCCTCAACCAGAACCACCACCAGCCCCCTT
Reg-T2R07C4	GAGCCGCCTTAAAGCCAGAATGGAGATGATAC
Reg-T2R07C6	GCCAGCAGCCTTGATATTCACAAACGGGGT
Reg-T2R08C3	TCACCGGAAACGTCACCAATGAATTATTCA
Reg-T2R08C5	ATTAGCGTCCGTAATCAGTAGCGAATTGAGGG
Reg-T2R09C2	AGGCCGGAACCAGAGCCACCACCG
Reg-T2R09C4	TAGCAGCATTGCCATCTTTTCATACACCCTCA
Reg-T2R09C6	AGTTTGCGCATTTTCGGTCATAGAGCCGCC
Reg-T2R10C1	GCCATTTGCAAACGTAGAAAATACCTGGCATG
Reg-T2R10C3	TTAAAGGTACATATAAAAGAAACAAACGCA
Reg-T2R10C5	AGGGAAGGATAAGTTTATTTTGTCAGCCGAAC
Reg-T2R11C2	AGGTGGCAGAATTATCACCGTCACCATTAGCA
Reg-T2R11C4	ACCACGGATAAATATTGACGGAAAACCATCGA

Strand name	Sequence
Reg-T2R11C6	TAGAAAAGGCGACATTCAACCGCAGAATCA
Reg-T2R12C3	ATAATAACTCAGAGAGATAACCCGAAGCGC
Reg-T2R12C5	AAAGTTACGCCCAATAATAAGAGCAGCCTTTA
Reg-T2R13C2	CGCTAATAGGAATACCCAAAAGAAATACATAA
Reg-T2R13C4	TGAGTTAACAGAAGGAAACCGAGGGCAAAGAC
Reg-T2R13C6	ATGAAATGAAAAGTAAGCAGATACAATCAA
Reg-T2R14C3	ATTAGACGGAGCGTCTTTCCAGAGCTACAA
Reg-T2R14C5	CAGAGAGAACAAAATAAACAGCCATTAAATCA
Reg-T2R15C4	TGCCAGTTATAACATAAAAACAGGACAAGAAT
Reg-T2R15C6	ATCCCAAAAAAAATGAAAATAGCAAGAAACA
Reg-T2R16C5	AGATTAGTATATAGAAGGCTTATCCAAGCCGT
Reg-T2R17C4	CAAATCAGTGCTATTTTGCACCCAGCCTAATT
Reg-T2R17C6	TAAGAACGGAGGTTTTGAAGCCTATTATTT
Reg-T2R19C6	CTTATCACTCATCGAGAACAAGCGGTATTC
Reg-T2R21C5	AGCTAATGCAGAACGCGAGAAAAATAATATCCTGTCTTTC
Reg-T3R01C6	AGAATATCAGACGACGACAATAAA
Reg-T3R03C5	TCATATGCGTTATACAAAGGCGTT
Reg-T3R03C6	CCAGTATGAATCGCCATATTTAGTAATAAG
Reg-T3R04C5	AAATAAGAACTTTTTCAAATATATCTGAGAGA
Reg-T3R05C6	ATTTCATGACCGTGTGATAAATAATTCTTA
Reg-T3R06C3	TATATAACGTAAATCGTCGCTATATTTGAA
Reg-T3R06C5	CTACCTTTAGAATCCTTGAAAACAAGAAAACA
Reg-T3R07C4	TTTCCCTTTTAACCTCCGGCTTAGCAAAGAAC
Reg-T3R07C6	GCTTAGAATCAAAATCATAGGTTTTAGTTA
Reg-T3R08C3	TTACCTTTACAATAACGGATTCGCAAAATT
Reg-T3R08C5	AAATTAATACCAAGTTACAAAATCCTGAATAA
Reg-T3R09C2	CGGGAGAATTTAATGGAAACAGTA
Reg-T3R09C4	CTTTGAATTACATTTAACAATTTCTAATTAAT
Reg-T3R09C6	GCGAATTATGAAACAAACATCATAGCGATA
Reg-T3R10C1	GTAGATTTGTTATTAATTTTAAAAAACAATTC
Reg-T3R10C3	ATTTGCACCATTTTGCGGAACAAATTTGAG
Reg-T3R10C5	TGGAAGGGAGCGGAATTATCATCAACTAATAG
Reg-T3R11C2	AACATTATGTAAAACAGAAATAAATTTTACAT
Reg-T3R11C4	CCAGAAGGTTAGAACCTACCATATCCTGATTG
Reg-T3R11C6	ATTATCAGTTTGGATTATACTTGCGCAGAG
Reg-T3R12C3	GATTTAGATTGCTGAACCTCAAAGTATTAA
Reg-T3R12C5	ATTAGAGCAATATCTGGTCAGTTGCAGCAGAA
Reg-T3R13C2	GCATCACCAGTATTAGACTTTACAGTTTGAGT
Reg-T3R13C4	CCTCAATCCGTCAATAGATAATACAGAAACCA
Reg-T3R13C6	ACAGTTGTTAGGAGCACTAACATATTCCTG
Reg-T3R14C3	CACCGCCTGAAAGCGTAAGAATACATTCTG
Reg-T3R14C5	GATAAAACTTTTTGAATGGCTATTTTCACCAG
Reg-T3R15C4	AGACAATAAGAGGTGAGGCGGTCATATCAAAC
Reg-T3R15C6	ATGCGCGTACCGAACGAACCACGCAAATCA
Reg-T3R16C5	TCACACGATGCAACAGGAAAAACGGAAGAACT
Reg-T3R17C4	CCAGCCATCCAGTAATAAAAGGGACGTGGCAC
Reg-T3R17C6	AATACCTATTTACATTGGCAGAAGTCTTTA
Reg-T3R19C6	TTAACCGTCACTTGCCTGAGTACTCATGGA
Reg-T3R21C5	CTAAACAGGAGGCCGATAATCCTGAGAAGTGTCACGCAAA
Reg-T4R01C6	GCGCGTACTTTCCTCGTTAGAATC
Reg-T4R03C5	AAAGCCGGCGAACGTGTGCCGTAA
Reg-T4R03C6	GGAAGGGGGCAAGTGTAGCGGTGCTACAGG
Reg-T4R04C5	AGCACTAAAAAGGGCGAAAAACCGAAATCCCT
Reg-T4R05C6	GGCGATGTTTTTGGGGTCGAGGGCGAGAAA
Reg-T4R06C3	TGAGTGTTCAGCTGATTGCCCTTGCGCGGG
Reg-T4R06C5	TATAAATCGAGAGTTGCAGCAAGCGTCGTGCC
Reg-T4R07C4	GGCCCTGAAAAAGAATAGCCCGAGCGTGGACT

Strand name	Sequence
Reg-T4R07C6	CTGGTTTGTTCCGAAATCGGCATCTATCAG
Reg-T4R08C3	GAGAGGCGACAACATACGAGCCGCTGCAGG
Reg-T4R08C5	AGCTGCATAGCCTGGGGTGCCTAAGTAAAACG
Reg-T4R09C2	AATTCCACGTTTGCGTATTGGGCG
Reg-T4R09C4	AAGTGTAATAATGAATCGGCCAACCACCGCCT
Reg-T4R09C6	CTAACTCCCAGTCGGGAAACCTGGTCCACG
Reg-T4R10C1	GAATTCGTGCCATTCGCCATTCAGTTCCGGCA
Reg-T4R10C3	TCGACTCTGAAGGGCGATCGGTGCGGCCTC
Reg-T4R10C5	ACGGCCAGTACGCCAGCTGGCGAACATCTGCC
Reg-T4R11C2	ACTGTTGGAGAGGATCCCCGGGTACCGCTCAC
Reg-T4R11C4	TTCGCTATTGCCAAGCTTGCATGCGAAGCATA
Reg-T4R11C6	GTGCTGCCCCAGTCACGACGTTTGAGTGAG
Reg-T4R12C3	AGGAAGATCATTAAATGTGAGCGTTTTTAA
Reg-T4R12C5	AGTTTGAGATTCTCCGTGGGAACAATTCGCAT
Reg-T4R13C2	TTCATCAACGCACTCCAGCCAGCTGCTGCGCA
Reg-T4R13C4	CCCGTCGGGGGACGACGACAGTATCGGGCCTC
Reg-T4R13C6	ATTGACCCGCATCGTAACCGTGAGGGGGAT
Reg-T4R14C3	CCAATAGGAAACTAGCATGTCAAGGAGCAA
Reg-T4R14C5	TAAATTTTTGATAATCAGAAAAGCACAAAGGC
Reg-T4R15C4	ACCCCGGTTGTTAAATCAGCTCATAGTAACAA
Reg-T4R15C6	CAGGAAGTAATATTTTGTTAAAAACGGCGG
Reg-T4R16C5	TATCAGGTAAATCACCATCAATATCAATGCCT
Reg-T4R17C4	AGACAGTCCATTGCCTGAGAGTCTTCATATGT
Reg-T4R17C6	ACCGTTCATTTTTGAGAGATCTCCCAAAAA
Reg-T4R19C6	CCTTTATCATATATTTTAAATGGATATTCA
Reg-T4R21C5	AATCATACAGGCAAGGCAGAGCATAAAGCTAAGGGAGAAG

Supplementary Table 2 | Bridge staples.

Strand name	Sequence
Bri-T1R02C5	GATACATTTCGCTTTTTTGACCCTGTAAT
Bri-T1R05C4	AAGCGAACAATTGCTGAATATAATGCTGTATTTTTTTGTGAGAAAGGCCGG
Bri-T1R07C2	TGGATAGCAAGCCCGATTTTTAATCGTAAACGCCAT
Bri-T1R08C1	CAGAGGGGGTTTTGCCTTCCTGTAGCCAGCT
Bri-T1R12C1	AGGACAGATGATTTTTTCACCAGTAGCACCATTACCGACTTGA
Bri-T1R14C2	TGCCACTACTTTTTTTGCCACCCTC
Bri-T1R16C3	ACAACCATTTTTTCATACATGGCTTTTAAGCGCA
Bri-T1R18C5	GAGAATAGAAAGGAACAACTATTTTCTCAAGAGAAGGA
Bri-T1R19C5	TGTCGTCTCAGCCCTCATATTTTTTTCGCCACCCTCAGGTGTATC
Bri-T2R02C5	ACCGTACTCAGGTTTTTGATCTAAAGTTT
Bri-T2R05C4	AGGAGTGTAAACATGAAAGTATTAAGAGGCTTTTTTTGCGAATAATAATTT
Bri-T2R07C2	AGAACCGCATTTACCGTTTTACCGATATATACGTAA
Bri-T2R08C1	GAACCGCCTCTTTACCTAAAACGAAAGAGGC
Bri-T2R10C0	GGAATTAGAGCTTTTTTTTCAGACCAGGCGCGTTGGGAAGATTTTTTTTCCAGGCAAAGC
Bri-T2R12C1	ATTAAGACTCCTTTTTAATATACAGTAACAGTACCGAAATTGC
Bri-T2R14C2	AACTGAACATTTTTTTTGAATAACC
Bri-T2R16C3	TTTTATCTTTTTTATCCAATCGCAAGAGTTGGGT
Bri-T2R18C5	TTTTATTTTCATCGTAGGAATTTTTAGCCTGTTTAGTA
Bri-T2R19C5	TAATCGGCCATCCTAATTTTTTTTTTTTTCGAGCCAACAACGCC
Bri-T3R02C5	AACATGTAATTTTTTTTGAAACCAATCAA
Bri-T3R05C4	GCGAGAAAATAAACACCGGAATCATAATTATTTTTTTCGCCCAATAGCAAG
Bri-T3R07C2	TTGCTTCTTATATGTATTTTACGCTAACGGAGAATT
Bri-T3R08C1	CATAAATCAATTTAGTCAGAGGGTAATTGAG
Bri-T3R12C1	GACAACTCGTATTTTTTCCTGTGTGAAATTGTTATCCGAGCTC
Bri-T3R14C2	GCCACGCTGTTTTTTTACCAGTGAG
Bri-T3R16C3	GCCAACATTTTTTCCACTATTAAAGAAATAGGGT
Bri-T3R18C5	CAAACTATCGGCCTTGCTGGTTTTTGAGCTTGACGGGG
Bri-T3R19C5	CTGTCCATTTTTATAATCATTTTTTTCTTAATGCGCCCACGCTGC
Bri-T4R02C5	GCGTAACCACCATTTTTGAGTAAAAGAGT
Bri-T4R05C4	CCAACGTCATCGGAACCCTAAAGGGAGCCCTTTTTTTGAACAATATTACCG
Bri-T4R07C2	ACGGGCAAGTTCCAGTTTTTTCTGACCTGCAACAGT
Bri-T4R08C1	CCAGGGTGGTTTTGCAAATGAAAAATCTAAA
Bri-T4R10C0	AATCATGGTCATTTTTTTTTTTGCCCGAACTCAGGTTTAACTTTTTTTTCAGTATGTTAG
Bri-T4R12C1	CCGCTTCTGGTTTTTTCGTTAATAAAACGAACTAAATTATACC
Bri-T4R14C2	CAAAAATAATTTTTTTTGTTTAGAC
Bri-T4R16C3	ACAAGAGTTTTTTTCGCGTTTTAATTCAAAAAGA
Bri-T4R18C5	GAGTAATGTGTAGGTAAAGATTTTTTGTTTTAAATATG
Bri-T4R19C5	ACTTTTGCATCGGTTGTACTTTTTTTAACCTGTTTAGGACCATTA

Supplementary Table 3 | Edge staples.

Strand name	Sequence
Edg-2G1T3C7R00	GTGTAAAGTAATTCTGTCAAAGTACCGACAAAAG
Edg-2G1T3C7R02	ATAGTAGGGCTTAATTGAAAAGCCAACGCTCAAC
Edg-2G1T3C7R04	TCAATGGTTTGAAATACCCTTCTGACCTAAATTT
Edg-2G1T3C7R06	CGAGTCAATAGTGAATTTTTAAGACGCTGAGAAG
Edg-2G1T3C7R08	GCTGAGCAAAAGAAGATGATTCATTTCAATTACC
Edg-2G1T3C7R10	GACAATATAATCCTGATTGATGATGGCAATTCAT
Edg-2G1T3C7R12	TAGTTATCTAAAATATCTAAAGGAATTGAGGAAG
Edg-2G1T3C7R14	GAACATCGCCATTAAAAAAACTGATAGCCCTAAA
Edg-2G1T3C7R16	TATCGTCTGAAATGGATTACATTTTGACGCTCAA
Edg-2G1T3C7R18	ATTTGATTAGTAATAACATTGTAGCAATACTTCT
Edg-2G1T3C7R20	AAAGGAACGGTACGCCAGTAAAGGGATTTTAGAC
Edg-2G1T4C7R00	GTGAGCACGTATAACGTGCTATGGTTGCTTTGAC
Edg-2G1T4C7R04	TCATCACCCAAATCAAGTGCCCACTACGTGAACC
Edg-2G1T4C7R10	GAGTAACGCCAGGGTTTTAAGGCGATTAAGTTGG
Edg-2G1T4C7R14	GATTTAAATTGTAAACGTATTGTATAAGCAAATA
Edg-2G1T4C7R16	TAGCCGGAGAGGGTAGCTTAGCTGATAAATTAAT
Edg-2G1T4C7R20	AATAAGCAATAAAGCCTCAAAGAATTAGCAAAAT
Edg-2G2T1C7R00	ATGGTGGCATCAATTCTAGGGCGCGAGCTGAAAA
Edg-2G2T1C7R02	ATTCCCAATTCTGCGAACCCATATAACAGTTGAT
Edg-2G2T1C7R06	GACCATAAATCAAAAATCCAGAAAACGAGAATGA
Edg-2G2T1C7R10	CAGAAACACCAGAACGAGAGGCTTGCCCTGACGA
Edg-2G2T1C7R12	TGCTGATAAATTGTGTCGAGATTTGTATCATCGC
Edg-2G2T1C7R14	AGGAACGAGGGTAGCAACGCGAAAGACAGCATCG
Edg-2G2T1C7R16	CCGGTTTATCAGCTTGCTAGCCTTTAATTGTATC
Edg-2G2T1C7R18	TCGGGATTTTGCTAAACAAATGAATTTTCTGTAT
Edg-2G2T1C7R20	AGACAAACTACAACGCCTGAGTTTCGTCACCAGT
Edg-2G2T3C7R00	ATGTAAAGTAATTCTGTCAAAGTACCGACAAAAG
Edg-2G2T3C7R02	ATAGTAGGGCTTAATTGAAAAGCCAACGCTCAAC
Edg-2G2T3C7R04	CGAATGGTTTGAAATACCCTTCTGACCTAAATTT
Edg-2G2T3C7R06	GAAGTCAATAGTGAATTTTTAAGACGCTGAGAAG
Edg-2G2T3C7R08	TATGAGCAAAAGAAGATGATTCATTTCAATTACC
Edg-2G2T3C7R10	CACAATATAATCCTGATTGATGATGGCAATTCAT
Edg-2G2T3C7R12	TGGTTATCTAAAATATCTAAAGGAATTGAGGAAG
Edg-2G2T3C7R14	AGACATCGCCATTAAAAAAACTGATAGCCCTAAA
Edg-2G2T3C7R16	CCTCGTCTGAAATGGATTACATTTTGACGCTCAA
Edg-2G2T3C7R18	TCTTGATTAGTAATAACATTGTAGCAATACTTCT
Edg-2G2T4C7R00	ATGAGCACGTATAACGTGCTATGGTTGCTTTGAC
Edg-2G2T4C7R02	ATCGGGCGCTAGGGCGCTAAGAAAGCGAAAGGAG
Edg-2G2T4C7R04	CGATCACCCAAATCAAGTGCCCACTACGTGAACC
Edg-2G2T4C7R06	GAATCCTGTTTGATGGTGGCCCCAGCAGGCGAAA
Edg-2G2T4C7R08	TAGCTCACTGCCCGCTTTACATTAATTGCGTTGC
Edg-2G2T4C7R10	CAGTAACGCCAGGGTTTTAAGGCGATTAAGTTGG
Edg-2G2T4C7R12	TGCGTTGGTGTAGATGGGGTAATGGGATAGGTCA
Edg-2G2T4C7R14	AGTTTAAATTGTAAACGTATTGTATAAGCAAATA
Edg-2G2T4C7R16	CCGCCGGAGAGGGTAGCTTAGCTGATAAATTAAT
Edg-2G2T4C7R18	TCAAATTTTTAGAACCCTTTCAACGCAAGGATAA
Edg-2G2T4C7R20	AGTAAGCAATAAAGCCTCAAAGAATTAGCAAAAT
Edg-2G3T1C7R00	ACGGTGGCATCAATTCTAGGGCGCGAGCTGAAAA
Edg-2G3T1C7R02	CTTCCCAATTCTGCGAACCCATATAACAGTTGAT
Edg-2G3T1C7R04	AAATTGCTCCTTTTGATATTAGAGAGTACCTTTA
Edg-2G3T1C7R06	AACCATAAATCAAAAATCCAGAAAACGAGAATGA
Edg-2G3T1C7R08	AGCGAGGCATAGTAAGAGACGCCAAAAGGAATTA
Edg-2G3T1C7R10	ATGAAACACCAGAACGAGAGGCTTGCCCTGACGA
Edg-2G3T1C7R12	CCCTGATAAATTGTGTCGAGATTTGTATCATCGC
Edg-2G3T1C7R14	AGGAACGAGGGTAGCAACGCGAAAGACAGCATCG

Strand name	Sequence
Edg-2G3T1C7R16	TTGGTTTATCAGCTTGCTAGCCTTTAATTGTATC
Edg-2G3T1C7R18	ACGGGATTTTGCTAAACAAATGAATTTTCTGTAT
Edg-2G3T1C7R20	AGACAAACTACAACGCCTGAGTTTCGTCACCAGT
Edg-2G3T3C7R00	ACGTAAAGTAATTCTGTCAAAGTACCGACAAAAG
Edg-2G3T3C7R02	CTAGTAGGGCTTAATTGAAAAGCCAACGCTCAAC
Edg-2G3T3C7R04	AAAATGGTTTGAAATACCCTTCTGACCTAAATTT
Edg-2G3T3C7R06	AAAGTCAATAGTGAATTTTTAAGACGCTGAGAAG
Edg-2G3T3C7R08	AGTGAGCAAAAGAAGATGATTCATTTCAATTACC
Edg-2G3T3C7R10	ATCAATATAATCCTGATTGATGATGGCAATTCAT
Edg-2G3T3C7R12	CCGTTATCTAAAATATCTAAAGGAATTGAGGAAG
Edg-2G3T3C7R14	AGACATCGCCATTAAAAAAACTGATAGCCCTAAA
Edg-2G3T3C7R16	TTTCGTCTGAAATGGATTACATTTTGACGCTCAA
Edg-2G3T3C7R18	ACTTGATTAGTAATAACATTGTAGCAATACTTCT
Edg-2G3T3C7R20	AGAGGAACGGTACGCCAGTAAAGGGATTTTAGAC
Edg-2G4T2C7R00	ATAGCCACCACCCTCATTGAACCGCCACCCTCAG
Edg-2G4T2C7R02	AAGAGAGGGTTGATATAAGCGGATAAGTGCCGTC
Edg-2G4T2C7R04	ATGTATAAACAGTTAATGTTGAGTAACAGTGCCC
Edg-2G4T2C7R06	TAGCAGGTCAGACGATTGTTGACAGGAGGTTGAG
Edg-2G4T2C7R08	CATAGCGCGTTTTCATCGCTTTAGCGTCAGACTG
Edg-2G4T2C7R10	GGGCGCCAAAGACAAAAGTTCATATGGTTTACCA
Edg-2G4T2C7R12	GCCCGAAGCCCTTTTTAAAGCAATAGCTATCTTA
Edg-2G4T2C7R14	AATTTTTTGTTTAACGTCTCCAAATAAGAAACGA
Edg-2G4T2C7R16	CCAACCTCCCGACTTGCGGCGAGGCGTTTTAGCG
Edg-2G4T2C7R18	AGTAAACCAAGTACCGCATTCCAAGAACGGGTAT
Edg-2G4T2C7R20	ACAGATAAGTCCTGAACACCTGTTTATCAACAAT
Edg-2G4T4C7R00	ATGAGCACGTATAACGTGCTATGGTTGCTTTGAC
Edg-2G4T4C7R02	AACGGGCGCTAGGGCGCTAAGAAAGCGAAAGGAG
Edg-2G4T4C7R04	ATATCACCCAAATCAAGTGCCCACTACGTGAACC
Edg-2G4T4C7R06	TAATCCTGTTTGATGGTGGCCCCAGCAGGCGAAA
Edg-2G4T4C7R12	GCCGTTGGTGTAGATGGGGTAATGGGATAGGTCA
Edg-2G4T4C7R16	CCGCCGGAGAGGGTAGCTTAGCTGATAAATTAAT
Edg-2G4T4C7R20	ACTAAGCAATAAAGCCTCAAAGAATTAGCAAAAT
Edg-2bRT1C7R00	GGTGGCATCAATTCTAGGGCGCGAGCTGAA
Edg-2bRT1C7R02	TCCCAATTCTGCGAACCCATATAACAGTTG
Edg-2bRT1C7R04	ATTGCTCCTTTTGATATTAGAGAGTACCTT
Edg-2bRT1C7R06	CCATAAATCAAAAATCCAGAAAACGAGAAT
Edg-2bRT1C7R08	CGAGGCATAGTAAGAGACGCCAAAAGGAAT
Edg-2bRT1C7R10	GAAACACCAGAACGAGAGGCTTGCCCTGAC
Edg-2bRT1C7R12	CTGATAAATTGTGTCGAGATTTGTATCATC
Edg-2bRT1C7R14	GAACGAGGGTAGCAACGCGAAAGACAGCAT
Edg-2bRT1C7R16	GGTTTATCAGCTTGCTAGCCTTTAATTGTA
Edg-2bRT1C7R18	GGGATTTTGCTAAACAAATGAATTTTCTGT
Edg-2bRT1C7R20	ACAAACTACAACGCCTGAGTTTCGTCACCA
Edg-2bRT2C7R00	AGCCACCACCCTCATTGAACCGCCACCCTC
Edg-2bRT2C7R02	GAGAGGGTTGATATAAGCGGATAAGTGCCG
Edg-2bRT2C7R04	GTATAAACAGTTAATGTTGAGTAACAGTGC
Edg-2bRT2C7R06	GCAGGTCAGACGATTGTTGACAGGAGGTTG
Edg-2bRT2C7R08	TAGCGCGTTTTCATCGCTTTAGCGTCAGAC
Edg-2bRT2C7R10	GCGCCAAAGACAAAAGTTCATATGGTTTAC
Edg-2bRT2C7R12	CCGAAGCCCTTTTTAAAGCAATAGCTATCT
Edg-2bRT2C7R14	TTTTTTGTTTAACGTCTCCAAATAAGAAAC
Edg-2bRT2C7R16	AACCTCCCGACTTGCGGCGAGGCGTTTTAG
Edg-2bRT2C7R18	TAAACCAAGTACCGCATTCCAAGAACGGGT
Edg-2bRT2C7R20	AGATAAGTCCTGAACACCTGTTTATCAACA
Edg-2bRT3C7R00	GTAAAGTAATTCTGTCAAAGTACCGACAAA
Edg-2bRT3C7R02	AGTAGGGCTTAATTGAAAAGCCAACGCTCA
Edg-2bRT3C7R04	AATGGTTTGAAATACCCTTCTGACCTAAAT

Strand name	Sequence
Edg-2bRT3C7R06	AGTCAATAGTGAATTTTTAAGACGCTGAGA
Edg-2bRT3C7R08	TGAGCAAAAGAAGATGATTCATTTCAATTA
Edg-2bRT3C7R10	CAATATAATCCTGATTGATGATGGCAATTC
Edg-2bRT3C7R12	GTTATCTAAAATATCTAAAGGAATTGAGGA
Edg-2bRT3C7R14	ACATCGCCATTAAAAAAACTGATAGCCCTA
Edg-2bRT3C7R16	TCGTCTGAAATGGATTACATTTTGACGCTC
Edg-2bRT3C7R18	TTGATTAGTAATAACATTGTAGCAATACTT
Edg-2bRT3C7R20	AGGAACGGTACGCCAGTAAAGGGATTTTAG
Edg-2bRT4C7R00	GAGCACGTATAACGTGCTATGGTTGCTTTG
Edg-2bRT4C7R02	CGGGCGCTAGGGCGCTAAGAAAGCGAAAGG
Edg-2bRT4C7R04	ATCACCCAAATCAAGTGCCCACTACGTGAA
Edg-2bRT4C7R06	ATCCTGTTTGATGGTGGCCCCAGCAGGCGA
Edg-2bRT4C7R08	GCTCACTGCCCGCTTTACATTAATTGCGTT
Edg-2bRT4C7R10	GTAACGCCAGGGTTTTAAGGCGATTAAGTT
Edg-2bRT4C7R12	CGTTGGTGTAGATGGGGTAATGGGATAGGT
Edg-2bRT4C7R14	TTTAAATTGTAAACGTATTGTATAAGCAAA
Edg-2bRT4C7R16	GCCGGAGAGGGTAGCTTAGCTGATAAATTA
Edg-2bRT4C7R18	AAATTTTTAGAACCCTTTCAACGCAAGGAT
Edg-2bRT4C7R20	TAAGCAATAAAGCCTCAAAGAATTAGCAAA
Edg-1bRT2C7R14	TTTTTTGTTTAACGTCTCCAAATAAGAAACG
Edg-1bRT2C7R16	AACCTCCCGACTTGCGGCGAGGCGTTTTAGC
Edg-1bRT2C7R18	TAAACCAAGTACCGCATTCCAAGAACGGGTA
Edg-1bRT2C7R20	AGATAAGTCCTGAACACCTGTTTATCAACAA
Edg-1bG2T3C7R00	TGTAAAGTAATTCTGTCAAAGTACCGACAAAAG
Edg-1bG2T3C7R02	TAGTAGGGCTTAATTGAAAAGCCAACGCTCAAC
Edg-1bG2T3C7R04	GAATGGTTTGAAATACCCTTCTGACCTAAATTT
Edg-1bG2T3C7R06	AAGTCAATAGTGAATTTTTAAGACGCTGAGAAG
Edg-5bRT1C7R00	GGTGGCATCAATTCTAGGGCGCGAGCT
Edg-5bRT1C7R02	TCCCAATTCTGCGAACCCATATAACAG
Edg-5bRT1C7R18	GGGATTTTGCTAAACAAATGAATTTTC
Edg-5bRT1C7R20	ACAAACTACAACGCCTGAGTTTCGTCA
Edg-5bRT2C7R00	AGCCACCACCCTCATTGAACCGCCACC
Edg-5bRT2C7R02	GAGAGGGTTGATATAAGCGGATAAGTG
Edg-5bRT2C7R18	TAAACCAAGTACCGCATTCCAAGAACG
Edg-5bRT2C7R20	AGATAAGTCCTGAACACCTGTTTATCA
Edg-5bRT3C7R00	GTAAAGTAATTCTGTCAAAGTACCGAC
Edg-5bRT3C7R02	AGTAGGGCTTAATTGAAAAGCCAACGC
Edg-5bRT3C7R18	TTGATTAGTAATAACATTGTAGCAATA
Edg-5bRT3C7R20	AGGAACGGTACGCCAGTAAAGGGATTT
Edg-5bRT4C7R00	GAGCACGTATAACGTGCTATGGTTGCT
Edg-5bRT4C7R02	CGGGCGCTAGGGCGCTAAGAAAGCGAA
Edg-5bRT4C7R18	AAATTTTTAGAACCCTTTCAACGCAAG
Edg-5bRT4C7R20	TAAGCAATAAAGCCTCAAAGAATTAGC
Edg-5bG1T3C7R00	CCAGTGTAAAGTAATTCTGTCAAAGTACCGACAAAAG
Edg-5bG1T3C7R02	TGTATAGTAGGGCTTAATTGAAAAGCCAACGCTCAAC
Edg-5bG1T3C7R18	TTGATTTGATTAGTAATAACATTGTAGCAATACTTCT
Edg-5bG1T3C7R20	GAAAAAGGAACGGTACGCCAGTAAAGGGATTTTAGAC
Edg-5bG2T4C7R00	ACAATGAGCACGTATAACGTGCTATGGTTGCTTTGAC
Edg-5bG2T4C7R02	GGTATCGGGCGCTAGGGCGCTAAGAAAGCGAAAGGAG
Edg-5bG2T4C7R18	CCGTCAAATTTTTAGAACCCTTTCAACGCAAGGATAA
Edg-5bG2T4C7R20	CTCAGTAAGCAATAAAGCCTCAAAGAATTAGCAAAAT
Edg-5bG3T1C7R00	TAGACGGTGGCATCAATTCTAGGGCGCGAGCTGAAAA
Edg-5bG3T1C7R02	CTTCTTCCCAATTCTGCGAACCCATATAACAGTTGAT
Edg-5bG3T1C7R18	TCAACGGGATTTTGCTAAACAAATGAATTTTCTGTAT
Edg-5bG3T1C7R20	AAAAGACAAACTACAACGCCTGAGTTTCGTCACCAGT
Edg-5bG4T2C7R00	AAAATAGCCACCACCCTCATTGAACCGCCACCCTCAG
Edg-5bG4T2C7R02	GATAAGAGAGGGTTGATATAAGCGGATAAGTGCCGTC

Strand name	Sequence
Edg-5bG4T2C7R18	AGGAGTAAACCAAGTACCGCATTCCAAGAACGGGTAT
Edg-5bG4T2C7R20	TTGACAGATAAGTCCTGAACACCTGTTTATCAACAAT
Edg-5bC2G1T3R18	TTGATTAGTAATAACATTGTAGCAATACTTCTTCCCA
Edg-5bC2G1T3R20	AGGAACGGTACGCCAGTAAAGGGATTTTAGACGGTGG
Edg-5bC2G2T4R18	AAATTTTTAGAACCCTTTCAACGCAAGGATAAGAGAG
Edg-5bC2G2T4R20	TAAGCAATAAAGCCTCAAAGAATTAGCAAAATAGCCA
Edg-5bC2G3T1R18	GGGATTTTGCTAAACAAATGAATTTTCTGTATAGTAG
Edg-5bC2G3T1R20	ACAAACTACAACGCCTGAGTTTCGTCACCAGTGTAAA
Edg-5bC2G4T2R18	TAAACCAAGTACCGCATTCCAAGAACGGGTATCGGGC
Edg-5bC2G4T2R20	AGATAAGTCCTGAACACCTGTTTATCAACAATGAGCA
Edg-5bC2RT1R00	CATCAATTCTAGGGCGCGAGCTGAAAA
Edg-5bC2RT1R02	ATTCTGCGAACCCATATAACAGTTGAT
Edg-5bC2RT2R00	CCACCCTCATTGAACCGCCACCCTCAG
Edg-5bC2RT2R02	GGTTGATATAAGCGGATAAGTGCCGTC
Edg-5bC2RT3R00	GTAATTCTGTCAAAGTACCGACAAAAG
Edg-5bC2RT3R02	GGCTTAATTGAAAAGCCAACGCTCAAC
Edg-5bC2RT4R00	CGTATAACGTGCTATGGTTGCTTTGAC
Edg-5bC2RT4R02	GCTAGGGCGCTAAGAAAGCGAAAGGAG
Edg-T1R02C7-DHP	GTGTCGTAGACACTCCCAATTCTGCGAACCCATATAACAGTTGATGTGTCGTAGACAC
Edg-T1R06C7-DHP	GTGTCGTAGACACCCATAAATCAAAAATCCAGAAAACGAGAATGAGTGTCGTAGACAC
Edg-T1R10C7-DHP	GTGTCGTAGACACGAAACACCAGAACGAGAGGCTTGCCCTGACGAGTGTCGTAGACAC
Edg-T1R14C7-DHP	GTGTCGTAGACACGAACGAGGGTAGCAACGCGAAAGACAGCATCGGTGTCGTAGACAC
Edg-T1R18C7-DHP	GTGTCGTAGACACGGGATTTTGCTAAACAAATGAATTTTCTGTATGTGTCGTAGACAC
Edg-T2R02C7-DHP	GTGTCGTAGACACGAGAGGGTTGATATAAGCGGATAAGTGCCGTCGTGTCGTAGACAC
Edg-T2R06C7-DHP	GTGTCGTAGACACGCAGGTCAGACGATTGTTGACAGGAGGTTGAGGTGTCGTAGACAC
Edg-T2R10C7-DHP	GTGTCGTAGACACGCGCCAAAGACAAAAGTTCATATGGTTTACCAGTGTCGTAGACAC
Edg-T2R14C7-DHP	GTGTCGTAGACACTTTTTTGTTTAACGTCTCCAAATAAGAAACGAGTGTCGTAGACAC
Edg-T2R18C7-DHP	GTGTCGTAGACACTAAACCAAGTACCGCATTCCAAGAACGGGTATGTGTCGTAGACAC
Edg-T3R02C7-DHP	GTGTCGTAGACACAGTAGGGCTTAATTGAAAAGCCAACGCTCAACGTGTCGTAGACAC
Edg-T3R06C7-DHP	GTGTCGTAGACACAGTCAATAGTGAATTTTTAAGACGCTGAGAAGGTGTCGTAGACAC
Edg-T3R10C7-DHP	GTGTCGTAGACACCAATATAATCCTGATTGATGATGGCAATTCATGTGTCGTAGACAC
Edg-T3R14C7-DHP	GTGTCGTAGACACACATCGCCATTAAAAAAACTGATAGCCCTAAAGTGTCGTAGACAC
Edg-T3R18C7-DHP	GTGTCGTAGACACTTGATTAGTAATAACATTGTAGCAATACTTCTGTGTCGTAGACAC
Edg-T4R02C7-DHP	GTGTCGTAGACACCGGGCGCTAGGGCGCTAAGAAAGCGAAAGGAGGTGTCGTAGACAC
Edg-T4R06C7-DHP	GTGTCGTAGACACATCCTGTTTGATGGTGGCCCCAGCAGGCGAAAGTGTCGTAGACAC
Edg-T4R10C7-DHP	GTGTCGTAGACACGTAACGCCAGGGTTTTAAGGCGATTAAGTTGGGTGTCGTAGACAC
Edg-T4R14C7-DHP	GTGTCGTAGACACTTTAAATTGTAAACGTATTGTATAAGCAAATAGTGTCGTAGACAC
Edg-T4R18C7-DHP	GTGTCGTAGACACAAATTTTTAGAACCCTTTCAACGCAAGGATAAGTGTCGTAGACAC
Edg-T3C7R20	AGGAACGGTACGCCAGTAAAGGGATTTTAGAC
Edg-ROX-T2C7R00	/5Rox-N/AGCCACCACCCTCATTGAACCGCCACCCTCAG
Edg-T3C7R20-Q	AGGAACGGTACGCCAGTAAAGGGATTTTAGAC/3IAbRQ/
Edg-TYE-T2C7R00	/5TYE563/AGCCACCACCCTCATTGAACCGCCACCCTCAG
Edg-T4C7R14-ROX	AGTTTAAATTGTAAACGTATTGTATAAGCAAATA/3Rox-N/
Edg-Q-T2C7R06	/5IAbRQ/GCAGGTCAGACGATTGTTGACAGGAGGTTG
Edg-T3C7R16-ROX	TCGTCTGAAATGGATTACATTTTGACGCTC/3Rox-N/
Edg-Q-T1C7R04	/5IAbRQ/AAATTGCTCCTTTTGATATTAGAGAGTACCTTTA
Edg-ROX-T2C7R00	/56-ROXN/ATAGCCACCACCCTCATTGAACCGCCACCCTCAG
Edg-T4C7R20-Q	TAAGCAATAAAGCCTCAAAGAATTAGCAAA/3IAbRQSp/

Supplementary Table 4 | Pattern staples.

Strand name	Sequence
ssEx-Reg-T1R01C6	TCATTTGCTAATAGTAGTAGCATTTTTTTTTTTTTTTTTTTTTT
ssEx-Reg-T1R03C5	CAACTAAAGTACGGTGGATGGCTTTTTTTTTTTTTTTTTTTTTT
ssEx-Reg-T1R03C6	TTTCATTGAGTAGATTTAGTTTCTATATTTTTTTTTTTTTTTTTTTTTTT
ssEx-Reg-T1R04C5	TAGAGCTTCAGACCGGAAGCAAACCTATTATATTTTTTTTTTTTTTTTTTTT
ssEx-Reg-T1R05C6	GTCAGGAAGAGGTCATTTTGCTCTGGAAGTTTTTTTTTTTTTTTTTTTTT
ssEx-Reg-T1R06C3	TTAAGAGGGTCCAATACTGCGGATAGCGAGTTTTTTTTTTTTTTTTTTTT
ssEx-Reg-T1R06C5	GTCAGAAGATTGAATCCCCCTCAACCTCGTTTTTTTTTTTTTTTTTTTTTTT
ssEx-Reg-T1R07C4	AAATATTCCAAAGCGGATTGCATCGAGCTTCATTTTTTTTTTTTTTTTTTTT
ssEx-Reg-T1R07C6	AACAGTTAGGTCTTTACCCTGATCCAACAGTTTTTTTTTTTTTTTTTTTT
ssEx-Reg-T1R08C3	AGGCTTTTCAGGTAGAAAGATTCAATTACCTTTTTTTTTTTTTTTTTTTT
ssEx-Reg-T1R08C5	ACCAGACGGAATACCACATTCAACGAGATGGTTTTTTTTTTTTTTTTTTTTT
ssEx-Reg-T1R09C2	CATTATTAGCAAAAGAAGTTTTGCTTTTTTTTTTTTTTTTTTTT
ssEx-Reg-T1R09C4	AGATTTAGACGATAAAAACCAAAAATCGTCATTTTTTTTTTTTTTTTTTTTT
ssEx-Reg-T1R09C6	ATACATACAACACTATCATAACATGCTTTATTTTTTTTTTTTTTTTTTTT
ssEx-Reg-T1R10C1	AGTCAGGACATAGGCTGGCTGACCTTTGAAAGTTTTTTTTTTTTTTTTTTTT
ssEx-Reg-T1R10C3	TTATGCGATTGACAAGAACCGGAGGTCAATTTTTTTTTTTTTTTTTTTTT
ssEx-Reg-T1R10C5	TTAATTTCCAACGTAACAAAGCTGTCCATGTTTTTTTTTTTTTTTTTTTTTT
ssEx-Reg-T1R11C2	GAGTAATCTTTTAAGAACTGGCTCCGGAACAATTTTTTTTTTTTTTTTTTTT
ssEx-Reg-T1R11C4	ACCCAAATAACTTTAATCATTGTGATCAGTTGTTTTTTTTTTTTTTTTTTTT
ssEx-Reg-T1R11C6	GTGAATATAGTAAATTGGGCTTTAATGCAGTTTTTTTTTTTTTTTTTTTT
ssEx-Reg-T1R12C3	CATAAGGGACACTAAAACACTCACATTAAATTTTTTTTTTTTTTTTTTTT
ssEx-Reg-T1R12C5	ACTTAGCCATTATACCAAGCGCGAGAGGACTATTTTTTTTTTTTTTTTTTTT
ssEx-Reg-T1R13C2	AAAAGAATAACCGAACTGACCAACTTCATCAATTTTTTTTTTTTTTTTTTTT
ssEx-Reg-T1R13C4	CCCCAGCGGGAACGAGGCGCAGACTATTCATTTTTTTTTTTTTTTTTTTTTT
ssEx-Reg-T1R13C6	ACAACGGAAATCCGCGACCTGCCTCATTCATTTTTTTTTTTTTTTTTTTT
ssEx-Reg-T1R14C3	CGGGTAAAATTCGGTCGCTGAGGAATGACATTTTTTTTTTTTTTTTTTTT
ssEx-Reg-T1R14C5	AAGACTTTGGCCGCTTTTGCGGGATTAAACAGTTTTTTTTTTTTTTTTTTTT
ssEx-Reg-T1R15C4	GAGTTAAATTCATGAGGAAGTTTCTCTTTGACTTTTTTTTTTTTTTTTTTTT
ssEx-Reg-T1R15C6	CTCAGCAGGCTACAGAGGCTTTAACAAAGTTTTTTTTTTTTTTTTTTTTT
ssEx-Reg-T1R16C5	CTTGATACTGAAAATCTCCAAAAAAGCGGAGTTTTTTTTTTTTTTTTTTTTT
ssEx-Reg-T1R17C4	TTTCACGTCGATAGTTGCGCCGACCTTGCAGGTTTTTTTTTTTTTTTTTTTT
ssEx-Reg-T1R17C6	CAAAAGGTTCGAGGTGAATTTCTCGTCACCTTTTTTTTTTTTTTTTTTTT
ssEx-Reg-T1R19C6	GTTAGTAACTTTCAACAGTTTCAAAGGCTCTTTTTTTTTTTTTTTTTTTT
ssEx-Reg-T1R21C5	CCATGTACCGTAACACTGTAGCATTCCACAGATTCCAGACTTTTTTTTTTTTTTTTTTTT
ssEx-Reg-T2R01C6	ACCCTCATTCAGGGATAGCAAGCCTTTTTTTTTTTTTTTTTTTT
ssEx-Reg-T2R03C5	TTAGGATTAGCGGGGTGGAACCTATTTTTTTTTTTTTTTTTTTT
ssEx-Reg-T2R03C6	GTACCAGGTATAGCCCGGAATAGAACCGCCTTTTTTTTTTTTTTTTTTTT
ssEx-Reg-T2R04C5	TTATTCTGACTGGTAATAAGTTTTAACAAATATTTTTTTTTTTTTTTTTTTT
ssEx-Reg-T2R05C6	CAGTGCCCCCCCTGCCTATTTCTTTGCTCATTTTTTTTTTTTTTTTTTTT
ssEx-Reg-T2R06C3	GTCTCTGACACCCTCAGAGCCACATCAAAATTTTTTTTTTTTTTTTTTTT
ssEx-Reg-T2R06C5	AATCCTCAACCAGAACCACCACCAGCCCCCTTTTTTTTTTTTTTTTTTTTTT
ssEx-Reg-T2R07C4	GAGCCGCCTTAAAGCCAGAATGGAGATGATACTTTTTTTTTTTTTTTTTTTT
ssEx-Reg-T2R07C6	GCCAGCAGCCTTGATATTCACAAACGGGGTTTTTTTTTTTTTTTTTTTTT
ssEx-Reg-T2R08C3	TCACCGGAAACGTCACCAATGAATTATTCATTTTTTTTTTTTTTTTTTTT
ssEx-Reg-T2R08C5	ATTAGCGTCCGTAATCAGTAGCGAATTGAGGGTTTTTTTTTTTTTTTTTTTT
ssEx-Reg-T2R09C2	AGGCCGGAACCAGAGCCACCACCGTTTTTTTTTTTTTTTTTTTT
ssEx-Reg-T2R09C4	TAGCAGCATTGCCATCTTTTCATACACCCTCATTTTTTTTTTTTTTTTTTTT
ssEx-Reg-T2R09C6	AGTTTGCGCATTTTCGGTCATAGAGCCGCCTTTTTTTTTTTTTTTTTTTT
ssEx-Reg-T2R10C1	GCCATTTGCAAACGTAGAAAATACCTGGCATGTTTTTTTTTTTTTTTTTTTT
ssEx-Reg-T2R10C3	TTAAAGGTACATATAAAAGAAACAAACGCATTTTTTTTTTTTTTTTTTTT
ssEx-Reg-T2R10C5	AGGGAAGGATAAGTTTATTTTGTCAGCCGAACTTTTTTTTTTTTTTTTTTTT
ssEx-Reg-T2R11C2	AGGTGGCAGAATTATCACCGTCACCATTAGCATTTTTTTTTTTTTTTTTTTT
ssEx-Reg-T2R11C4	ACCACGGATAAATATTGACGGAAAACCATCGATTTTTTTTTTTTTTTTTTTT
ssEx-Reg-T2R11C6	TAGAAAAGGCGACATTCAACCGCAGAATCATTTTTTTTTTTTTTTTTTTT
ssEx-Reg-T2R12C3	ATAATAACTCAGAGAGATAACCCGAAGCGCTTTTTTTTTTTTTTTTTTTT

Strand name	Sequence
ssEx-Reg-T2R12C5	AAAGTTACGCCCAATAATAAGAGCAGCCTTTATTTTTTTTTTTTTTTTTTTT
ssEx-Reg-T2R13C2	CGCTAATAGGAATACCCAAAAGAAATACATAATTTTTTTTTTTTTTTTTTTT
ssEx-Reg-T2R13C4	TGAGTTAACAGAAGGAAACCGAGGGCAAAGACTTTTTTTTTTTTTTTTTTTT
ssEx-Reg-T2R13C6	ATGAAATGAAAAGTAAGCAGATACAATCAATTTTTTTTTTTTTTTTTTTT
ssEx-Reg-T2R14C3	ATTAGACGGAGCGTCTTTCCAGAGCTACAATTTTTTTTTTTTTTTTTTTT
ssEx-Reg-T2R14C5	CAGAGAGAACAAAATAAACAGCCATTAAATCATTTTTTTTTTTTTTTTTTTT
ssEx-Reg-T2R15C4	TGCCAGTTATAACATAAAAACAGGACAAGAATTTTTTTTTTTTTTTTTTTTT
ssEx-Reg-T2R15C6	ATCCCAAAAAAAATGAAAATAGCAAGAAACATTTTTTTTTTTTTTTTTTT
ssEx-Reg-T2R16C5	AGATTAGTATATAGAAGGCTTATCCAAGCCGTTTTTTTTTTTTTTTTTTTTT
ssEx-Reg-T2R17C4	CAAATCAGTGCTATTTTGCACCCAGCCTAATTTTTTTTTTTTTTTTTTTTTT
ssEx-Reg-T2R17C6	TAAGAACGGAGGTTTTGAAGCCTATTATTTTTTTTTTTTTTTTTTTTTTT
ssEx-Reg-T2R19C6	CTTATCACTCATCGAGAACAAGCGGTATTCTTTTTTTTTTTTTTTTTTTT
ssEx-Reg-T2R21C5	AGCTAATGCAGAACGCGAGAAAAATAATATCCTGTCTTTCTTTTTTTTTTTTTTTTTTTT
ssEx-Reg-T3R01C6	AGAATATCAGACGACGACAATAAATTTTTTTTTTTTTTTTTTTT
ssEx-Reg-T3R03C5	TCATATGCGTTATACAAAGGCGTTTTTTTTTTTTTTTTTTTTTT
ssEx-Reg-T3R03C6	CCAGTATGAATCGCCATATTTAGTAATAAGTTTTTTTTTTTTTTTTTTTT
ssEx-Reg-T3R04C5	AAATAAGAACTTTTTCAAATATATCTGAGAGATTTTTTTTTTTTTTTTTTTT
ssEx-Reg-T3R05C6	ATTTCATGACCGTGTGATAAATAATTCTTATTTTTTTTTTTTTTTTTTTT
ssEx-Reg-T3R06C3	TATATAACGTAAATCGTCGCTATATTTGAATTTTTTTTTTTTTTTTTTTT
ssEx-Reg-T3R06C5	CTACCTTTAGAATCCTTGAAAACAAGAAAACATTTTTTTTTTTTTTTTTTTT
ssEx-Reg-T3R07C4	TTTCCCTTTTAACCTCCGGCTTAGCAAAGAACTTTTTTTTTTTTTTTTTTTT
ssEx-Reg-T3R07C6	GCTTAGAATCAAAATCATAGGTTTTAGTTATTTTTTTTTTTTTTTTTTTT
ssEx-Reg-T3R08C3	TTACCTTTACAATAACGGATTCGCAAAATTTTTTTTTTTTTTTTTTTTTT
ssEx-Reg-T3R08C5	AAATTAATACCAAGTTACAAAATCCTGAATAATTTTTTTTTTTTTTTTTTTT
ssEx-Reg-T3R09C2	CGGGAGAATTTAATGGAAACAGTATTTTTTTTTTTTTTTTTTTT
ssEx-Reg-T3R09C4	CTTTGAATTACATTTAACAATTTCTAATTAATTTTTTTTTTTTTTTTTTTTT
ssEx-Reg-T3R09C6	GCGAATTATGAAACAAACATCATAGCGATATTTTTTTTTTTTTTTTTTTT
ssEx-Reg-T3R10C1	GTAGATTTGTTATTAATTTAAAAAACAATTCTTTTTTTTTTTTTTTTTTTTT
ssEx-Reg-T3R10C3	ATTTGCACCATTTTGCGGAACAAATTTGAGTTTTTTTTTTTTTTTTTTTT
ssEx-Reg-T3R10C5	TGGAAGGGAGCGGAATTATCATCAACTAATAGTTTTTTTTTTTTTTTTTTTT
ssEx-Reg-T3R11C2	AACATTATGTAAAACAGAAATAAATTTACATTTTTTTTTTTTTTTTTTTTTT
ssEx-Reg-T3R11C4	CCAGAAGGTTAGAACCTACCATATCCTGATTGTTTTTTTTTTTTTTTTTTTT
ssEx-Reg-T3R11C6	ATTATCAGTTTGGATTATACTTGCGCAGAGTTTTTTTTTTTTTTTTTTTT
ssEx-Reg-T3R12C3	GATTTAGATTGCTGAACCTCAAAGTATTAATTTTTTTTTTTTTTTTTTTT
ssEx-Reg-T3R12C5	ATTAGAGCAATATCTGGTCAGTTGCAGCAGAATTTTTTTTTTTTTTTTTTTT
ssEx-Reg-T3R13C2	GCATCACCAGTATTAGACTTTACAGTTTGAGTTTTTTTTTTTTTTTTTTTTT
ssEx-Reg-T3R13C4	CCTCAATCCGTCAATAGATAATACAGAAACCATTTTTTTTTTTTTTTTTTTT
ssEx-Reg-T3R13C6	ACAGTTGTTAGGAGCACTAACATATTCCTGTTTTTTTTTTTTTTTTTTTT
ssEx-Reg-T3R14C3	CACCGCCTGAAAGCGTAAGAATACATTCTGTTTTTTTTTTTTTTTTTTTT
ssEx-Reg-T3R14C5	GATAAAACTTTTTGAATGGCTATTTTCACCAGTTTTTTTTTTTTTTTTTTTT
ssEx-Reg-T3R15C4	AGACAATAAGAGGTGAGGCGGTCATATCAAACTTTTTTTTTTTTTTTTTTTT
ssEx-Reg-T3R15C6	ATGCGCGTACCGAACGAACCACGCAAATCATTTTTTTTTTTTTTTTTTTT
ssEx-Reg-T3R16C5	TCACACGATGCAACAGGAAAAACGGAAGAACTTTTTTTTTTTTTTTTTTTTT
ssEx-Reg-T3R17C4	CCAGCCATCCAGTAATAAAAGGGACGTGGCACTTTTTTTTTTTTTTTTTTTT
ssEx-Reg-T3R17C6	AATACCTATTTACATTGGCAGAAGTCTTTATTTTTTTTTTTTTTTTTTTT
ssEx-Reg-T3R19C6	TTAACCGTCACTTGCCTGAGTACTCATGGATTTTTTTTTTTTTTTTTTTT
ssEx-Reg-T3R21C5	CTAAACAGGAGGCCGATAATCCTGAGAAGTGTCACGCAAATTTTTTTTTTTTTTTTTTTT
ssEx-Reg-T4R01C6	GCGCGTACTTTCCTCGTTAGAATCTTTTTTTTTTTTTTTTTTTT
ssEx-Reg-T4R03C5	AAAGCCGGCGAACGTGTGCCGTAATTTTTTTTTTTTTTTTTTTT
ssEx-Reg-T4R03C6	GGAAGGGGGCAAGTGTAGCGGTGCTACAGGTTTTTTTTTTTTTTTTTTTT
ssEx-Reg-T4R04C5	AGCACTAAAAAGGGCGAAAAACCGAAATCCCTTTTTTTTTTTTTTTTTTTTT
ssEx-Reg-T4R05C6	GGCGATGTTTTTGGGGTCGAGGGCGAGAAATTTTTTTTTTTTTTTTTTTT
ssEx-Reg-T4R06C3	TGAGTGTTCAGCTGATTGCCCTTGCGCGGGTTTTTTTTTTTTTTTTTTTT
ssEx-Reg-T4R06C5	TATAAATCGAGAGTTGCAGCAAGCGTCGTGCCTTTTTTTTTTTTTTTTTTTT
ssEx-Reg-T4R07C4	GGCCCTGAAAAAGAATAGCCCGAGCGTGGACTTTTTTTTTTTTTTTTTTTTT
ssEx-Reg-T4R07C6	CTGGTTTGTTCCGAAATCGGCATCTATCAGTTTTTTTTTTTTTTTTTTTT
ssEx-Reg-T4R08C3	GAGAGGCGACAACATACGAGCCGCTGCAGGTTTTTTTTTTTTTTTTTTTT

Strand name	Sequence
ssEx-Reg-T4R08C5	AGCTGCATAGCCTGGGGTGCCTAAGTAAAACGTTTTTTTTTTTTTTTTTTTT
ssEx-Reg-T4R09C2	AATTCCACGTTTGCGTATTGGGCGTTTTTTTTTTTTTTTTTTTT
ssEx-Reg-T4R09C4	AAGTGTAATAATGAATCGGCCAACCACCGCCTTTTTTTTTTTTTTTTTTTTT
ssEx-Reg-T4R09C6	CTAACTCCCAGTCGGGAAACCTGGTCCACGTTTTTTTTTTTTTTTTTTTT
ssEx-Reg-T4R10C1	GAATTCGTGCCATTCGCCATTCAGTTCCGGCATTTTTTTTTTTTTTTTTTTT
ssEx-Reg-T4R10C3	TCGACTCTGAAGGGCGATCGGTGCGGCCTCTTTTTTTTTTTTTTTTTTTT
ssEx-Reg-T4R10C5	ACGGCCAGTACGCCAGCTGGCGAACATCTGCCTTTTTTTTTTTTTTTTTTTT
ssEx-Reg-T4R11C2	ACTGTTGGAGAGGATCCCCGGGTACCGCTCACTTTTTTTTTTTTTTTTTTTT
ssEx-Reg-T4R11C4	TTCGCTATTGCCAAGCTTGCATGCGAAGCATATTTTTTTTTTTTTTTTTTTT
ssEx-Reg-T4R11C6	GTGCTGCCCCAGTCACGACGTTTGAGTGAGTTTTTTTTTTTTTTTTTTTT
ssEx-Reg-T4R12C3	AGGAAGATCATTAAATGTGAGCGTTTTTAATTTTTTTTTTTTTTTTTTTT
ssEx-Reg-T4R12C5	AGTTTGAGATTCTCCGTGGGAACAATTCGCATTTTTTTTTTTTTTTTTTTTT
ssEx-Reg-T4R13C2	TTCATCAACGCACTCCAGCCAGCTGCTGCGCATTTTTTTTTTTTTTTTTTTT
ssEx-Reg-T4R13C4	CCCGTCGGGGGACGACGACAGTATCGGGCCTCTTTTTTTTTTTTTTTTTTTT
ssEx-Reg-T4R13C6	ATTGACCCGCATCGTAACCGTGAGGGGGATTTTTTTTTTTTTTTTTTTTT
ssEx-Reg-T4R14C3	CCAATAGGAAACTAGCATGTCAAGGAGCAATTTTTTTTTTTTTTTTTTTT
ssEx-Reg-T4R14C5	TAAATTTTTGATAATCAGAAAAGCACAAAGGCTTTTTTTTTTTTTTTTTTTT
ssEx-Reg-T4R15C4	ACCCCGGTTGTTAAATCAGCTCATAGTAACAATTTTTTTTTTTTTTTTTTTT
ssEx-Reg-T4R15C6	CAGGAAGTAATATTTTGTTAAAAACGGCGGTTTTTTTTTTTTTTTTTTTT
ssEx-Reg-T4R16C5	TATCAGGTAAATCACCATCAATATCAATGCCTTTTTTTTTTTTTTTTTTTTT
ssEx-Reg-T4R17C4	AGACAGTCCATTGCCTGAGAGTCTTCATATGTTTTTTTTTTTTTTTTTTTTT
ssEx-Reg-T4R17C6	ACCGTTCATTTTTGAGAGATCTCCCAAAAATTTTTTTTTTTTTTTTTTTT
ssEx-Reg-T4R19C6	CCTTTATCATATATTTTAAATGGATATTCATTTTTTTTTTTTTTTTTTTT
ssEx-Reg-T4R21C5	AATCATACAGGCAAGGCAGAGCATAAAGCTAAGGGAGAAGTTTTTTTTTTTTTTTTTTTT

Supplementary Table 5 | Negation strands.

Strand name	Sequence
Neg-T1R00C7	TTTTCAGCTCGCGCCCTAGAATTGATGCCACC
Neg-T1R02C7	ATCAACTGTTATATGGGTTCGCAGAATTGGGA
Neg-T1R04C7	TAAAGGTACTCTCTAATATCAAAAGGAGCAAT
Neg-T1R06C7	TCATTCTCGTTTTCTGGATTTTTGATTTATGG
Neg-T1R08C7	TAATTCCTTTTGGCGTCTCTTACTATGCCTCG
Neg-T1R10C7	TCGTCAGGGCAAGCCTCTCGTTCTGGTGTTTC
Neg-T1R12C7	GCGATGATACAAATCTCGACACAATTTATCAG
Neg-T1R14C7	CGATGCTGTCTTTCGCGTTGCTACCCTCGTTC
Neg-T1R16C7	GATACAATTAAAGGCTAGCAAGCTGATAAACC
Neg-T1R18C7	ATACAGAAAATTCATTTGTTTAGCAAAATCCC
Neg-T1R20C7	ACTGGTGACGAAACTCAGGCGTTGTAGTTTGT
Neg-T2R00C7	CTGAGGGTGGCGGTTCAATGAGGGTGGTGGCT
Neg-T2R02C7	GACGGCACTTATCCGCTTATATCAACCCTCTC
Neg-T2R04C7	GGGCACTGTTACTCAACATTAACTGTTTATAC
Neg-T2R06C7	CTCAACCTCCTGTCAACAATCGTCTGACCTGC
Neg-T2R08C7	CAGTCTGACGCTAAAGCGATGAAAACGCGCTA
Neg-T2R10C7	TGGTAAACCATATGAACTTTTGTCTTTGGCGC
Neg-T2R12C7	TAAGATAGCTATTGCTTTAAAAAGGGCTTCGG
Neg-T2R14C7	TCGTTTCTTATTTGGAGACGTTAAACAAAAAA
Neg-T2R16C7	CGCTAAAACGCCTCGCCGCAAGTCGGGAGGTT
Neg-T2R18C7	ATACCCGTTCTTGGAATGCGGTACTTGGTTTA
Neg-T2R20C7	ATTGTTGATAAACAGGTGTTCAGGACTTATCT
Neg-T3R00C7	CTTTTGTCGGTACTTTGACAGAATTACTTTAC
Neg-T3R02C7	GTTGAGCGTTGGCTTTTCAATTAAGCCCTACT
Neg-T3R04C7	AAATTTAGGTCAGAAGGGTATTTCAAACCATT
Neg-T3R06C7	CTTCTCAGCGTCTTAAAAATTCACTATTGACT
Neg-T3R08C7	GGTAATTGAAATGAATCATCTTCTTTTGCTCA
Neg-T3R10C7	ATGAATTGCCATCATCAATCAGGATTATATTG
Neg-T3R12C7	CTTCCTCAATTCCTTTAGATATTTTAGATAAC
Neg-T3R14C7	TTTAGGGCTATCAGTTTTTTTAATGGCGATGT
Neg-T3R16C7	TTGAGCGTCAAAATGTAATCCATTTCAGACGA
Neg-T3R18C7	AGAAGTATTGCTACAATGTTATTACTAATCAA
Neg-T3R20C7	GTCTAAAATCCCTTTACTGGCGTACCGTTCCT
Neg-T4R00C7	GTCAAAGCAACCATAGCACGTTATACGTGCTC
Neg-T4R02C7	CTCCTTTCGCTTTCTTAGCGCCCTAGCGCCCG
Neg-T4R04C7	GGTTCACGTAGTGGGCACTTGATTTGGGTGAT
Neg-T4R06C7	TTTCGCCTGCTGGGGCCACCATCAAACAGGAT
Neg-T4R08C7	GCAACGCAATTAATGTAAAGCGGGCAGTGAGC
Neg-T4R10C7	CCAACTTAATCGCCTTAAAACCCTGGCGTTAC
Neg-T4R12C7	TGACCTATCCCATTACCCCATCTACACCAACG
Neg-T4R14C7	TATTTGCTTATACAATACGTTTACAATTTAAA
Neg-T4R16C7	ATTAATTTATCAGCTAAGCTACCCTCTCCGGC
Neg-T4R18C7	TTATCCTTGCGTTGAAAGGGTTCTAAAAATTT
Neg-T4R20C7	ATTTTGCTAATTCTTTGAGGCTTTATTGCTTA

Supplementary References

[1] Tikhomirov, G., Petersen, P. & Qian, L. Triangular DNA origami tilings. *Journal of the American Chemical Society* DOI: 10.1021/jacs.8b10609 (to appear).

[2] Papoulis, A. & Pillai, S. U. *Probability, random variables, and stochastic processes* (Tata McGraw-Hill Education, 2002).

[3] Petersen, P. Yield Calculator. `http://qianlab.caltech.edu/YieldCalculator` (2016).

[4] Soloveichik, D. CRNSimulator. `http://users.ece.utexas.edu/~soloveichik/crnsimulator.html` (2009).

[5] Wetmur, J. G. Hybridization and renaturation kinetics of nucleic acids. *Annual Review of Biophysics and Bioengineering* **5**, 337–361 (1976).

[6] Tikhomirov, G., Petersen, P. & Qian, L. Programmable disorder in random DNA tilings. *Nature Nanotechnology* **12**, 251–259 (2017).

[7] Tikhomirov, G., Petersen, P. & Qian, L. Fractal assembly of micrometre-scale DNA origami arrays with arbitrary patterns. *Nature* **552**, 67–71 (2017).

[8] Marras, A. E., Zhou, L., Su, H.-J. & Castro, C. E. Programmable motion of DNA origami mechanisms. *Proceedings of the National Academy of Sciences* **112**, 713–718 (2015).

[9] Gerling, T., Wagenbauer, K. F., Neuner, A. M. & Dietz, H. Dynamic DNA devices and assemblies formed by shape-complementary, non–base pairing 3D components. *Science* **347**, 1446–1452 (2015).

[10] Yan, H., Zhang, X., Shen, Z. & Seeman, N. C. A robust DNA mechanical device controlled by hybridization topology. *Nature* **415**, 62–65 (2002).

[11] Han, D., Pal, S., Liu, Y. & Yan, H. Folding and cutting DNA into reconfigurable topological nanostructures. *Nature Nanotechnology* **5**, 712–717 (2010).

[12] Goodman, R. P. *et al.* Reconfigurable, braced, three-dimensional DNA nanostructures. *Nature Nanotechnology* **3**, 93–96 (2008).

[13] Douglas, S. M., Bachelet, I. & Church, G. M. A logic-gated nanorobot for targeted transport of molecular payloads. *Science* **335**, 831–834 (2012).

[14] Song, J. *et al.* Reconfiguration of DNA molecular arrays driven by information relay. *Science* **357**, eaan3377 (2017).

[15] Maune, H. T. *et al.* Self-assembly of carbon nanotubes into two-dimensional geometries using DNA origami templates. *Nature Nanotechnology* **5**, 61–66 (2010).

[16] Knudsen, J. B. *et al.* Routing of individual polymers in designed patterns. *Nature Nanotechnology* **10**, 892–898 (2015).

[17] Pal, S., Deng, Z., Ding, B., Yan, H. & Liu, Y. DNA-origami-directed self-assembly of discrete silver-nanoparticle architectures. *Angewandte Chemie International Edition* **49**, 2700–2704 (2010).

[18] Pal, S. *et al.* DNA directed self-assembly of anisotropic plasmonic nanostructures. *Journal of the American Chemical Society* **133**, 17606–17609 (2011).

[19] Engheta, N. Circuits with light at nanoscales: optical nanocircuits inspired by metamaterials. *Science* **317**, 1698–1702 (2007).

[20] Deng, Z., Samanta, A., Nangreave, J., Yan, H. & Liu, Y. Robust DNA-functionalized core/shell quantum dots with fluorescent emission spanning from UV–vis to near-IR and compatible with DNA-directed self-assembly. *Journal of the American Chemical Society* **134**, 17424–17427 (2012).

[21] Lent, C. S., Isaksen, B. & Lieberman, M. Molecular quantum-dot cellular automata. *Journal of the American Chemical Society* **125**, 1056–1063 (2003).

[22] Imre, A. *et al.* Majority logic gate for magnetic quantum-dot cellular automata. *Science* **311**, 205–208 (2006).

[23] Wang, J., Zhou, Z., Yue, L., Wang, S. & Willner, I. Switchable triggered interconversion and reconfiguration of DNA origami dimers and their use for programmed catalysis. *Nano Letters* **18**, 2718–2724 (2018).

[24] Sherman, W. B. & Seeman, N. C. A precisely controlled DNA biped walking device. *Nano Letters* **4**, 1203–1207 (2004).

[25] Shin, J.-S. & Pierce, N. A. A synthetic DNA walker for molecular transport. *Journal of the American Chemical Society* **126**, 10834–10835 (2004).

[26] Yin, P., Yan, H., Daniell, X. G., Turberfield, A. J. & Reif, J. H. A unidirectional DNA walker that moves autonomously along a track. *Angewandte Chemie International Edition* **43**, 4906–4911 (2004).

[27] Bath, J., Green, S. J. & Turberfield, A. J. A free-running DNA motor powered by a nicking enzyme. *Angewandte Chemie International Edition* **117**, 4432–4435 (2005).

[28] Schiefer, N. & Winfree, E. Universal computation and optimal construction in the chemical reaction network-controlled tile assembly model. *Lecture Notes in Computer Science* **9211**, 34–54 (2015).

[29] Zhang, D. Y., Hariadi, R. F., Choi, H. M. & Winfree, E. Integrating DNA strand-displacement circuitry with DNA tile self-assembly. *Nature Communications* **4**, 1965 (2013).

[30] Stojanovic, M. N. & Stefanovic, D. A deoxyribozyme-based molecular automaton. *Nature Biotechnology* **21**, 1069–1074 (2003).

[31] Qian, L. & Winfree, E. Parallel and scalable computation and spatial dynamics with DNA-based chemical reaction networks on a surface. *Lecture Notes in Computer Science* **8727**, 114–131 (2014).

[32] Thubagere, A. J. *et al.* A cargo-sorting DNA robot. *Science* **357**, eaan6558 (2017).
