# Supplementary material for: Information-based autonomous reconfiguration in systems of interacting DNA nanostructures
Source: Nat Commun. 2018 Dec 18;9:5362. doi: 10.1038/s41467-018-07805-7 (PMC6299139; doi:10.1038/s41467-018-07805-7)
Supplement: Supplementary file 2 — Description of Additional Supplementary Files [file 41467_2018_7805_MOESM2_ESM.pdf]

Description of Additional Supplementary Files

File Name: Supplementary Movie 1
Description: Conceptual animation of tile displacement, in comparison to strand displacement, showing how DNA origami structures can interact with each other in a dynamic process and a possible scenario of how they can bend slightly to allow displacement.
